# TGF-β induces cholesterol accumulation to regulate the secretion of tumor-derived extracellular vesicles

**DOI:** 10.1186/s13046-025-03291-0

**Published:** 2025-02-06

**Authors:** Dorival Mendes Rodrigues-Junior, Chrysoula Tsirigoti, Konstantina Psatha, Dimitris Kletsas, Michalis Aivaliotis, Carl-Henrik Heldin, Aristidis Moustakas

**Affiliations:** 1 Department of Medical Biochemistry and Microbiology, Science for Life Laboratory, Biomedical Center, Uppsala University, Box 582, Uppsala, SE-751 23 Sweden; 2 Astra Zeneca, Pepparedsleden 1, Mölndal, SE-431 83 Sweden; 3 Laboratory of Biochemistry, School of Medicine, Faculty of Health Sciences, Aristotle University of Thessaloniki, Thessaloniki, GR-541 24 Greece; 4 Laboratory of Cell Proliferation & Ageing, Institute of Biosciences and Applications, National Centre for Scientific Research ‘Demokritos’, Athens, GR-153 10 Greece

**Keywords:** Cancer, Cholesterol, Extracellular, Vesicles, Matrix metalloproteinase, Transforming growth factor β

## Abstract

**Background:**

Cancer cells are avid extracellular vesicle (EV) producers. EVs transport transforming growth factor-β (TGF-β), which is commonly activated under late stages of cancer progression. Nevertheless, whether TGF-β signaling coordinates EV biogenesis is a relevant topic that remains minimally explored.

**Method:**

We sought after specific TGF-β pathway mediators that could regulate EV release. To this end, we used a large number of cancer cell models, coupled to EV cell biological assays, unbiased proteomic and transcriptomic screens, followed by signaling and cancer biology analyses, including drug resistance assays.

**Results:**

We report that TGF-β, by activating its type I receptor and MEK-ERK1/2 signaling, increased the numbers of EVs released by human cancer cells. Upon examining cholesterol as a mediator of EV biogenesis, we delineated a pathway whereby ERK1/2 acted by phosphorylating sterol regulatory element-binding protein-2 that transcriptionally induced 7-dehydrocholesterol reductase expression, thus raising cholesterol abundance at both cellular and EV levels. Notably, inhibition of MEK or cholesterol synthesis, which impaired TGF-β-induced EV secretion, sensitized cancer cells to chemotherapeutic drugs. Furthermore, proteomic profiling of two distinct EV populations revealed that EVs secreted by TGF-β-stimulated cells were either depleted or enriched for different sets of cargo proteins. Among these, latent-TGF-β1 present in the EVs was not affected by TGF-β signaling, while TGF-β pathway-related molecules (e.g., matrix metalloproteinases, including MMP9) were either uniquely enriched on EVs or strongly enhanced after TGF-β stimulation. EV-associated latent-TGF-β1 activated SMAD signaling, even when EV uptake was blocked by heparin, indicating competent signaling capacity from target cell surface receptors. MMP inhibitor or proteinase treatment blocked EV-mediated SMAD signaling, suggesting that EVs require MMP activity to release the active TGF-β from its latent complex, a function also linked to the EV-mediated transfer of pro-migratory potential and ability of cancer cells to survive in the presence of cytotoxic drugs.

**Conclusion:**

Hence, we delineated a novel signaling cascade that leads to high rates of EV generation by cancer cells in response to TGF-β, with cholesterol being a key intermediate step in this mechanism.

**Graphical Abstract:**

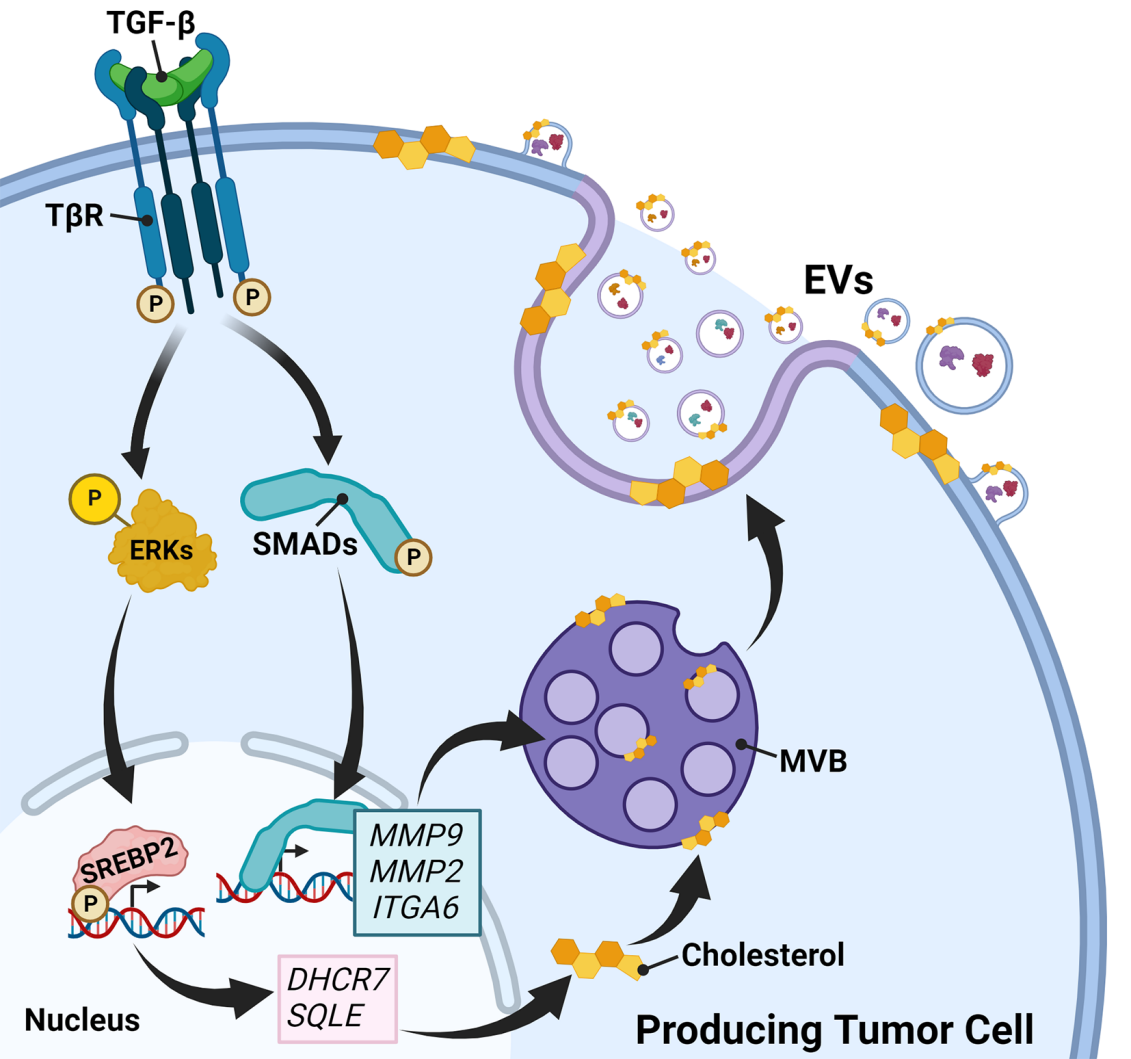

**•** TGF-β increases EV release by activating a MEK-ERK1/2-SREBP2-DHCR7 signaling and transcriptional pathway.
**•** TGF-β-induced DHCR7 expression raises cholesterol abundance that promotes EV release.
**•** EVs carry surface latent TGF-β and MMP9 that can activate TGF-β receptor signaling on the surface of recipient cells.

**Supplementary Information:**

The online version contains supplementary material available at 10.1186/s13046-025-03291-0.

## Blurb

The growth factor TGF-β orchestrates transcriptional signaling that enhances cholesterol biosynthesis and mediates release of extracellular vesicles. EV cargo further activate TGF-β signaling in recipient cells and promote tumor cell invasion and chemoresistance.

## Background

Extracellular vesicles (EVs) are cell-derived particles confined by a lipid bilayer that mediate intercellular communication by trafficking bioactive molecules (proteins, nucleic acids, lipids and carbohydrates) from secreting to recipient cells [1]. Major attention has been given to tumor-derived EVs due to the communication between cancer cells and cells in their microenvironment, as this is crucial for local tumor progression and the development of pre-metastatic niches at distant sites [2]. Whether blockage of EV-mediated tumorigenic processes may improve future treatment of cancer patients remains to be evaluated clinically [3]. Yet, multiple reports raise the utility of EVs and their molecular cargo as biomarkers with predictive and diagnostic potential that can guide cancer treatment decisions by molecular subclassification of tumors [4].

Among molecules carried by EVs, transforming growth factor β (TGF-β) has been steadily featured in cancer EV biology [5, 6]. TGF-β and its family members signal by binding to serine/threonine kinase receptors known as type I (TGFβRI) and type II (TGFβRII) receptors, which phosphorylate SMAD proteins (SMAD2 and SMAD3) and activate non-SMAD pathways, inducing MAP-kinases, Src and phosphatidylinositol-3´kinase/Akt, to mediate cytoplasmic signaling and regulate the expression of several genes [7]. The contextual responses leading to the dual role of TGF-β signaling in breast, colorectal, pancreatic, liver and lung adenocarcinoma is exemplified by the cell growth arrest and induction of cell death mediated by apoptosis in early-stage epithelial tumors, while the cancer cells that escape TGF-β suppression respond to TGF-β with tumorigenic immunosuppression, invasion and metastasis, thus promoting tumor progression [8]. TGF-β is implicated in epithelial-mesenchymal transition (EMT) and the maintenance of stem cell populations, which play an important role in anticancer therapy resistance [8]. Moreover, TGF-β enhances tumor angiogenesis and the development of cancer-associated fibroblasts and suppresses the immune system [8]. High numbers of circulating EVs carrying TGF-β were linked to the poor prognosis of cancer patients [6]. In different disease contexts, non-senescent fibroblasts stimulated with TGF-β1 secreted higher numbers of EVs [9], whereas, TGF-β1 stimulation did not affect the release of EVs from human pulmonary artery smooth muscle cells that are implicated in arterial hypertension [10]. Nevertheless, whether and how TGF-β can modulate tumor-derived EVs in order to assist tumorigenesis, remains poorly understood.

The term EVs collectively refers to a variety of secreted vesicles [11], such as exosomes, of endosomal origin, and microvesicles, originating from the plasma membrane [12]. Membrane budding followed by a fission process generates EVs secreted inside the lumen of multivesicular bodies (MVBs) or in the extracellular milieu [12]. EV biogenesis and sorting of their cargo is regulated by extracellular stimuli, which activate intracellular signaling, e.g. MEK/ERK1/2 kinases [13], and such signals act downstream via the endosomal sorting complexes required for transport (ESCRT) machinery, tetraspanins or synthesis of lipid/cholesterol constituents of EV membranes [12]. Hence, the present study aimed at examining whether TGF-β can regulate the secretion of tumor-derived EV subpopulations and their cargo. We elucidated mechanisms of TGF-β signaling contributing to EV release from breast cancer (BRCA) and lung adenocarcinoma (LUAD) cells, providing also insight into the protein composition of these EVs and their functional relevance as facilitators of oncogenic transformation.

## Methods

### Cells, reagents and treatments

Cell lines of human basal-TNBC (MDA-MB-231: HTB-26; Hs578T: HTB-126), luminal BRCA (ZR-75-1: CRL-1500; MCF7: HTB-22), LUAD (A549: CCL-185), non-small cell lung carcinoma (H1299: CRL-5803) and embryonic kidney epithelial-like (HEK293T: CRL-3216) were obtained from the American type culture collection (https://www.atcc.org/). Primary cultures of human breast fibroblasts (BNF2, BNF3) were previously described [14]. The cells were cultured in Dulbecco’s modified Eagle’s medium (DMEM; Sigma-Aldrich AB, Stockholm, Sweden) or Roswell Park Memorial Institute-1640 (ThermoFisher Scientific, Stockholm, Sweden), and supplemented with 10% fetal bovine serum (FBS; Biowest, Almeco A/S, Esbjerg, Denmark), 100 U/mL penicillin and 100 µg/mL streptomycin (Merck/Millipore, Stockholm, Sweden). The non-tumorigenic breast epithelial MCF10A cells were obtained from Dr Fred Miller (Barbara Ann Karmanos Cancer Institute, Detroit, USA) and cultured in DMEM/F12 (ThermoFisher Scientific, Stockholm, Sweden), supplemented with 5% horse serum (Biowest, Almeco A/S, Esbjerg, Denmark), 20 ng/mL epidermal growth factor (PeproTech EC Ltd/ThermoFisher Scientific, Stockholm, Sweden), 100 ng/mL cholera toxin, 0.5 µg/mL hydrocortisone, 10 µg/mL insulin (the last three from Sigma-Aldrich AB, Stockholm, Sweden). All cells were kept in a humidified incubator at 37 °C and 5% CO_2_. The cell lines were checked for mycoplasma presence and authenticated through STR profiling (Eurofins, Uppsala, Sweden). The cells were starved for 24 h in serum-free media prior to treatment for different time periods with 0.2-5.0 ng/mL recombinant human TGF-β1 (PreproTech EC Ltd/ThermoFisher Scientific, Stockholm, Sweden).

Cells were treated with inhibitors (i) LY2157299 (TβRi; #15409, Cayman Chemical Co, Ann Arbor, MI, USA), U0126 (MEKi; #19–147 Merck/Millipore, Stockholm, Sweden), AY9944 (DHCR7i; #14611, Cayman Chemical Co, Ann Arbor, MI, USA), simvastatin (HMGCRi; #S6196, Sigma-Aldrich AB, Stockholm, Sweden), heparin (#7980, STEMCELL Technologies UK Ltd, Cambridge, UK), GM6001 (MMPi; #CC1010, Sigma-Aldrich AB, Stockholm, Sweden), monoclonal anti-TGF-β1/2/3 antibody (TGF-β Ab; MAB1835, R&D Systems Inc./Biotechne, Abingdon, UK), gefitinib (EGFRi; #Y0001813, Sigma-Aldrich AB, Stockholm, Sweden), lapatinib (EGFRi# CDS022971, Sigma-Aldrich AB, Stockholm, Sweden), doxorubicin hydrochloride (Dox; #D1515, Sigma-Aldrich AB, Stockholm, Sweden) or paclitaxel (Taxol; #T7402, Sigma-Aldrich AB, Stockholm, Sweden). Drug toxicity was assessed after 48 h treatment by PrestoBlue HS Cell Viability Reagent (#P50200, ThermoFisher Scientific, Stockholm, Sweden), as previously described [15]. The fluorescence units from treated cells were normalized relative to those of vehicle-treated (0.1% dimethyl-sulfoxide (DMSO)) control cells. The 10 or 50% inhibitory concentration (IC_10_ or IC_50_) for the drugs was estimated based on dose-response curves. Experiments were performed in triplicates and data are expressed as mean ± SD.

### EV isolation and characterization

EV isolation and characterization was performed as previously described [15], following the MISEV2018 guidelines [11]. The cells were incubated for 48 h in DMEM supplemented with 10 µg/mL insulin (Sigma-Aldrich AB, Stockholm, Sweden), 1× MEM non-essential amino acids (#11140-050), 10 µg/mL fibroblast growth factor-basic (#13256-029), 1 mM sodium pyruvate (#11360-039) and 0.1 mM β-mercaptoethanol (#21985-023; all from ThermoFisher Scientific, Stockholm, Sweden), in the presence or absence of TGF-β1 or the respective drug treatment. Additionally, MCF10A cells were incubated in the culture media without horse serum. The serum-free medium was centrifuged at 1,200 × g for 5 min to clear cell debris while measuring the corresponding number of cells. The medium supernatant was filtered through a 0.2 μm filter, was further concentrated 20× by tangential flow filtration on a 50 kDa Ultra-15 Centrifugal Filter (Amicon; Merck/Millipore, Stockholm, Sweden) by centrifugation at 1,200 × g for 30 min, and defined as the vesicular secretome fraction (VSF). EVs smaller than 200 nm were sub-fractionated based on the presence of the tetraspanin CD81 protein [16] and on GM1-gangliosides, the latter having high binding affinity for cholerae toxin chain-B (CTB) [15]. VSF (150 µl) was incubated with 40 µL of anti-CD81-magnetic Dynabeads™ (#10616D, ThermoFisher Scientific, Stockholm, Sweden) or 0.5 µg of biotinylated CTB (#C34779; ThermoFisher Scientific, Stockholm, Sweden) in 100 µl of 0.2 μm filtered PBS, respectively, for 60 min at 37 °C. Pre-washed Dynabeads MyOne Streptavidin T1 (50 µl, #65602; ThermoFisher Scientific, Stockholm, Sweden) were added to the CTB-EV mixture and incubated for 30 min at 25 °C. The magnetic beads containing CD81-EVs or CTB-EVs were immobilized and the corresponding supernatant was collected as the respective specific EV-depleted fraction. The beads were washed with PBS and the isolated EVs or corresponding EV-depleted fractions were stored at -20 °C for up to 30 days.

Nanoparticle number and size distribution were measured through nanoparticle tracking analysis (NTA), (NanoSight system; Malvern Panalytical, Malvern, UK, equipped with a 532 nm laser and the NTA 3.4 analytic software). Modal diameter and per cell ratio of nanoparticles were analyzed statistically. To visualize EVs through transmission electron microscopy (TEM), VSF was mixed with an equal volume of 4% w/v formaldehyde and further incubated with uranyl oxalate, pH 7.0, for 5 min, then stained in a drop containing 4% w/v uranyl acetate and 2% w/v methylcellulose on ice for 10 min, as described [17]. Images were acquired on a Tecnai™ G2 Spirit BioTwin TEM (ThermoFisher Scientific/FEI, Stockholm, Sweden) at 80 kV with an ORIUS SC200 CCD camera and Gatan Digital Micrograph software (both from Gatan Inc./Blue Scientific, Pleasanton, CA, USA). In addition, cryogenic-transmission electron microscopy (cryo-TEM) imaging was performed to better characterize EVs. Briefly, the EVs (4.0 µL; 1 × 10^10^ nanoparticles/mL) were incubated twice for 30 s before blotting on a carbon QuantiFoil R 2/2 + 2 nm C (Jena Bioscience, Jena, Germany), followed by vitrification in liquid ethane using a Vitrobot Mark IV (ThermoFisher Scientific, Waltham, USA). The grids were loaded into a TFS Glacios (ThermoFisher Scientific, Waltham, USA) and the images were acquired at an acceleration voltage of 200 kV, total dose of 196 eÅ2 and defocus of -3 μm. The images were recorded by a Falcon 3EC direct-imaging camera. Moreover, CD81-EVs were suspended in 50 µl of PBS and fixed with 2% w/v formaldehyde, 2.5% w/v glutaraldehyde, 0.1 M sodium cacodylate, pH 7.2, before scanning on a FEI Quanta 250 FEG scanning electron microscope (SEM; ThermoFisher Scientific, Waltham, USA). For uptake assays, EVs present in the VSF were labelled using PKH26 red (#MIDI26-1KT, Sigma-Aldrich, Stockholm, Sweden). For functional assays on recipient cells, EVs present in the VSF or the corresponding EV-depleted fractions were used after particle (VSF) or volume equivalent (depleted fractions) directly, or after treatment with 25 µM MMPi or 40 µg/ml proteinase K for 30 min at 37 °C, the latter followed by incubation with complete protease inhibitors (#11697498001, Roche Diagnostics Scandinavia AB, Bromma, Sweden) for additional 30 min prior to their application into recipient cells.

### TEM of cell monolayers

Cells were fixed in 2.5% w/v glutaraldehyde, 1% w/v formaldehyde, 0.1 M PIPES, pH 7.4, and further incubated with 1% w/v osmium tetroxide (Analytical Standards AB, Landvetter, Sweden). The cells were placed in a 1:1 mixture of Epon Resin (Ted Pella Inc., Redding, CA, USA) and 99.9% v/v ethanol for 1 h, followed by two changes of 100% resin, the first for 2–4 h and the last overnight, until polymerization was achieved at 60 °C for 48 h. Ultrathin Sects. (50–70 nm) were cut in a Leica UC7 ultramicrotome (Leica Geosystems AB, Kista, Sweden) and placed on grids. The grids were contrasted in 5% w/v uranyl acetate and Reynold’s lead citrate for 10 and 2 min, respectively. Dried grids were examined in a Tecnai™ G2 Spirit BioTwin TEM (ThermoFisher Scientific/FEI, Stockholm, Sweden) at 80 kV with an ORIUS SC200 CCD camera and Gatan Digital Micrograph software (both from Gatan Inc./Blue Scientific, Pleasanton, CA, USA).

### Transferrin uptake

To assess endocytic uptake of transferrin (TRF), MDA-MB-231 and ZR-75-1 cells were placed on ice for 10 min and incubated for 1 h with 50 µg/mL Alexa Fluor-488-conjugated TRF (#T13342, ThermoFisher Scientific, Stockholm, Sweden). For flow cytometric analysis, 1 × 10^4^ live cells were counted as events according to the equipment’s setting (Acuri, BD Bioscences, Stockholm, Sweden). The samples were displayed in histograms and plotted according to the median of positive cells incorporating TRF. In addition, TRF uptake was evaluated by direct fluorescence in an inverted microscope (Nikon-Eclipse Ti-U, Nikon Europe B.V., Amstelveen, The Netherlands), equipped with a CCD camera (Andor multi pixel sCMOS camera, Oxford Instruments, Abingdon, UK). For this purpose, 50 × 10^4^ MDA-MB-231 cells were fixed with 4% w/v formaldehyde for 15 min and stained with 4′,6-diamidino-2-phenylindole (DAPI; Sigma-Aldrich AB, Stockholm, Sweden).

### Correlation analysis of gene expression

Comparisons of gene expression in tumor (BRCA, LUAD) versus normal samples were retrieved from the GEPIA2 server [18]. Overall survival in breast and lung cancer patients was predicted using the KM plotter database (KMplot) [19]. Samples were stratified and compared using auto-select cut-off with p-value calculated by log-Rank test. Using GEPIA2 RNA-seq cancer patient data, co-expression of *TGFB1* mRNA with GSEA hallmark signatures for TGF-β signaling, EMT, protein secretion and cholesterol homeostasis in LUAD and BRCA was analyzed (Supplementary Table S1) based on the Pearson correlation statistic (*R* > 0.2, p-value < 0.05). Correlation of gene expression and chemotherapy response in BRCA patients was conducted using the database from ROC Plotter (http://www.rocplot.org) [20]. Patients were categorized as non- or responders to treatment, according to the relapse-free survival status at 5 years post-surgery.

### RNA extraction and RT-qPCR

RNA extraction and real-time RT-qPCR were performed as previously described [21]. Gene expression levels were determined after normalization to *GAPDH* levels. Primer sequences of the genes are described in Supplementary Table S2.

### Immunoblotting

Immunoblotting was performed as previously described [21]. The lysis buffer contained complete protease inhibitors (#11697498001, Roche Diagnostics Scandinavia AB, Bromma, Sweden), phosphatase inhibitors (PhosSTOP; #4906837001, Sigma-Aldrich AB, Stockholm, Sweden), and protein content was determined using Bradford Reagent (#5000006, Bio-Rad Laboratories Inc., Sundbyberg, Sweden). In immunoblot analysis of EVs, equal volume of VSF or CD81- and CTB-enriched populations was analyzed in order to capture the difference in EV numbers contained in these preparations. The antibodies used in this study are described in Supplementary Table S3. ImageJ bundled with Java 1.8.0_172 (National Institutes of Health, Bethesda, MD, USA) was used to normalize intensity levels according to the expression of loading control proteins GAPDH, β-ACTIN or β-TUBULIN.

### siRNA transfection analysis

Once the cells reached 70% of confluence, transfections were performed with siLentFect (#170–3360, Bio-Rad Laboratories Inc., Sundbyberg, Sweden) and the respective siRNA (20 nM), according to the manufacturer. After 48 h, cells were collected for knockdown validation and seeded for further experiments. The human-specific siRNA oligonucleotides (Supplementary Table S4) were several distinct siRNAs against the target mRNA.

### Total cholesterol quantification

A cholesterol quantification Kit (#MAK043, Sigma-Aldrich AB, Stockholm, Sweden) was used to measure the amount of total cholesterol. Briefly, EVs or cells treated with TGF-β1 or inhibitors were extracted with 200 µL of chloroform: isopropanol: IGEPAL (7:11:0.1) in a micro-homogenizer for 5 min. The samples were centrifuged at 13,000 × *g* for 10 min and the organic phase was transferred to a new tube and dried at 50 °C to remove chloroform. The dried lipids were dissolved with 200 µL of the Cholesterol Assay Buffer and vortexed until the mixture was homogeneous. In a 96-well flat-bottom plate, 50 µL of the Reaction Mix was added to the respective wells with samples or blank. The cholesterol concentration was determined by colorimetry. The data were expressed as percentage of total cholesterol normalized to protein content, determined using Bradford Reagent (#5000006, Bio-Rad Laboratories Inc., Sundbyberg, Sweden).

### Liquid chromatography electrospray ionization tandem mass spectrometry (LC-ESI-MS/MS)

The protein profile of MDA-MB-231 CD81- and CTB-EVs was determined by LC-ESI-MS/MS and analyzed by label-free quantification, as previously described [22]. To approximately 70 µl of EV sample, 70 µl of lysis buffer was added (4% w/v SDS, 50 mM HEPES, pH 7.6, 1 mM DTT). Proteins were digested with trypsin (sequencing grade modified, Pierce/ThermoFisher Scientific, Stockholm, Sweden), using a modified protocol for SP3 protein clean-up [22], followed by SP3 peptide cleanup. Each sample was separated using a Thermo Scientific Dionex nano LC-system in a 3 h 5–40% ACN gradient coupled to Thermo Scientific High Field QExactive. The MS raw files were searched using Sequest-Percolator or Target Decoy PSM Validator under the software platform Proteome Discoverer 1.4 (ThermoFisher Scientific, Stockholm, Sweden) against the Uniprot *Homo sapiens* database and filtered to a 1% false discovery rate (FDR) cut-off. A precursor ion mass tolerance of 10 ppm was applied and product ion mass tolerances of 0.02 Da for HCD-FTMS and 0.8 Da for CID-ITMS. The algorithm considered tryptic peptides with maximum 2 missed cleavages, carbamidomethylation (C) as fixed modifications and oxidation (M) as variable modifications. All proteomics data have been deposited to PRIDE (https://www.ebi.ac.uk/pride/) under accession number PXD039591 and also presented in Supplementary Table S5.

Comparative label-free analysis of the proteomic profiles was performed using Perseus (version 1.6.14.0) by uploading the results obtained by the Proteome Discoverer, and following a previously established pipeline [23] with the following modifications. Prior to the analysis, the data were manually filtered in order to remove obsolete protein IDs from UniProt, duplicates and protein IDs corresponding to only one replicate. The data were normalized and transformed in logarithmic scale using log_2_ transformation and all the missing values were replaced by 0 using imputation in Perseus. Principal component analysis (PCA) in Perseus was used using as a cut-off method the Benjamini-Hochberg FDR with a cut-off value of 0.05. The volcano plot was made using -log(p-value) derived by t-test and the log_2_(Fold Change) of each comparison with an FDR value 0.05 and visualization with GraphPad. Protein-protein interaction networks for the unique or common proteins on CD81- or CTB-EVs (Ctrl versus TGF-β1-treatment) were constructed using STRING (version 11.5; http://string-db.org/), with the required high confidence score (> 0.7). Subsequent GO-biological process (BP), molecular function (MF), cellular component (CC) and Reactome enrichment analyses were performed.

### ELISA

Mature TGF-β1 (referred also as active TGF-β1) was measured using the human TGF-β1-Duoset ELISA sandwich kit according to the manufacturer’s instructions (#DB100C; R&D Systems, Oxon, UK). Acidification with 1 N HCl, followed by neutralization with 1.2 N NaOH/0.5 M HEPES at room temperature, was used to release the active TGF-β1 from its latent complex and measure the total TGF-β1 in the sample. Recombinant human latent TGF-β1 (L-TGF-β1: #SRP0300; Merck, Stockholm, Sweden) was used to monitor TGF-β1 activation induced by EVs.

### Gelatinase and collagenase proteolytic activity

The EnzChek™ Gelatinase/Collagenase Assay kit (ThermoFisher Scientific, Stockholm, Sweden) was used to evaluate gelatinase or collagenase activity associated to EVs. Briefly, 100 µg/mL of DQTM fluorescein-conjugate gelatin solution, was incubated for 1 h at room temperature with VSF, VSF depleted from CD81-positive EVs or in the presence of MMPi. Proteolytic activity, which is proportional to fluorescence intensity, was measured with a microplate reader (495 nm excitation/515 nm emission). For each condition, background fluorescence was corrected by subtracting the blank value. EV-free media was used as negative control and the relative normalized gelatin/collagen proteolytic activity is expressed as averages from biological triplicates.

### Luciferase assays

Luciferase assays in MDA-MB-231 and MCF10A cells transiently transfected with the CAGA_12_-luciferase promoter-reporter and treated with TGF-β1, EVs, EV-depleted fractions or inhibitors were performed using the firefly and renilla dual-luciferase Assay kit (Biotium, Fremont, CA, USA), as described [24]. Relative normalized luciferase activity is expressed as averages from triplicate determinations. Each experiment was repeated at least twice.

### Cell culture wound-healing assay

To evaluate migration, cells treated with TGF-β1 or EVs for 48 h were seeded (3 × 10^4^ cells/well) in complete medium into Culture-Insert 2 well 24 (#80242; Ibidi GmBh, Gräfelfing, Germany), as previously described [24]. The silicone insert was removed once the cells reached confluence and detached cells were removed by washing twice with PBS, followed by addition of fresh culture media. Wound closure was observed at different time points using a Zeiss Axioplan microscope (objective 10×) with MRC digital camera (Zeiss, Jena, Germany). Wound surface area was quantified by ImageJ as the percentage of open wound area per condition.

### Matrigel invasion assay

Transwell inserts (#351152) for 24-well plates (6.5 mm diameter, 8 μm pore) were coated with 300 µg/mL Matrigel matrix (#734 − 0269; both from Corning, New York City, NY, USA) diluted in coating buffer (10 mM Tris, pH 8.0, 0.7% w/v NaCl) and incubated at 37 °C for 1 h. Cells (5 × 10^4^) treated with VSF, VSF depleted from CD81-positive EVs or MMPi for 48 h were seeded in serum‐free DMEM in the upper chamber, and DMEM/10% FBS was placed in the lower chamber and incubated at 37 °C to allow the invasion through the Matrigel barrier for 18 h. After incubation, the inserts were fixed in methanol and stained with DAPI (Sigma-Aldrich AB, Stockholm, Sweden). Nuclei were counted in 10 pictures per insert, taken with the 20× objective, using ImageJ. Data were expressed as the percentage of invasion based on the ratio of the mean number of cells invading through Matrigel matrix per mean number of cells in the uncoated support.

### Immuno- and direct fluorescence microscopy

The cells were seeded in a 12-well plate followed by treatments with TGF-β1, VSF or VSF^+ PKH26^ for the indicated time periods. Immunofluorescence was performed as previously described [25]. Fixed cells were incubated with primary antibodies (Supplementary Table S3) in 1% BSA/PBS overnight at 4 °C, followed by incubation with Alexa-Fluor-488 or Alexa-Fluor-546 secondary antibodies (Supplementary Table S3) at a dilution of 1:500 in PBS for 1 h at room temperature. Cell nuclei were stained with DAPI (Sigma-Aldrich AB, Stockholm, Sweden) at a dilution 1:1,000 in PBS for 10 min at room temperature. The cells were examined by an inverted microscope (Nikon-Eclipse Ti-U, Nikon Europe B.V., Amstelveen, The Netherlands) equipped with a CCD camera (Andor multi pixel sCMOS camera, Oxford Instruments, Abingdon, UK). Ten to 15 random pictures were taken with 10× or 20× objectives at the same exposure time. When appropriate, the scores were given in a blind manner.

### Zebrafish extravasation assay

Staging and embryo production of Tg(Fli1:EGFP) zebrafish (*Danio rerio*), whose vasculature is marked in green, were conducted as described [25]. Empirical determination of sample size that provided power for discrimination between conditions, was used. For this reason, 200 embryos were injected per condition in order to reach a final number of viable embryos of more than 100. No randomization method was applied and the microinjector was blinded to the groups of injected cancer cells. MDA-MB-231 cells treated or not with VSF^Ctrl^ or VSF^+ TGF−β1^ were stained with 4 ng/µl CM-Dil Dye (ThermoFisher Scientific, Stockholm, Sweden) for 30 min at 37 °C. At 48 h post-fertilization, approximately 400 CM-Dil Dye-labelled cells in the presence or absence of doxorubicin (5 µM) were micro-injected as described [25]. Based on pre-established criteria, only microscopy-verified, correctly injected and viable zebrafish were retained at 34 °C and imaged automatically (ImageXpress Nano, Molecular Devices) 24 and 48 h post-implantation; cells extravasated from the circulation at the posterior part of the fish were counted (150 zebrafish per cell line and biological condition).

### Statistical analyses

All data (except when otherwise indicated) are presented as the mean ± SEM. Comparisons were performed using one-way or two-way ANOVA, followed by multiple paired comparisons conducted by means of the Tukey’s or Bonferroni’s post-test method when applicable. The Mann–Whitney test was used to evaluate associations between gene expression and chemotherapy treatment response. Additional statistical methods are explained in individual figure legends. The data were analyzed with GraphPad Prism 10.1 (GraphPad Software, San Diego, CA). A p-value < 0.05 was necessary to determine statistically significant differences.

## Results

### TGF-β induces EV release

**Fig. 1:**
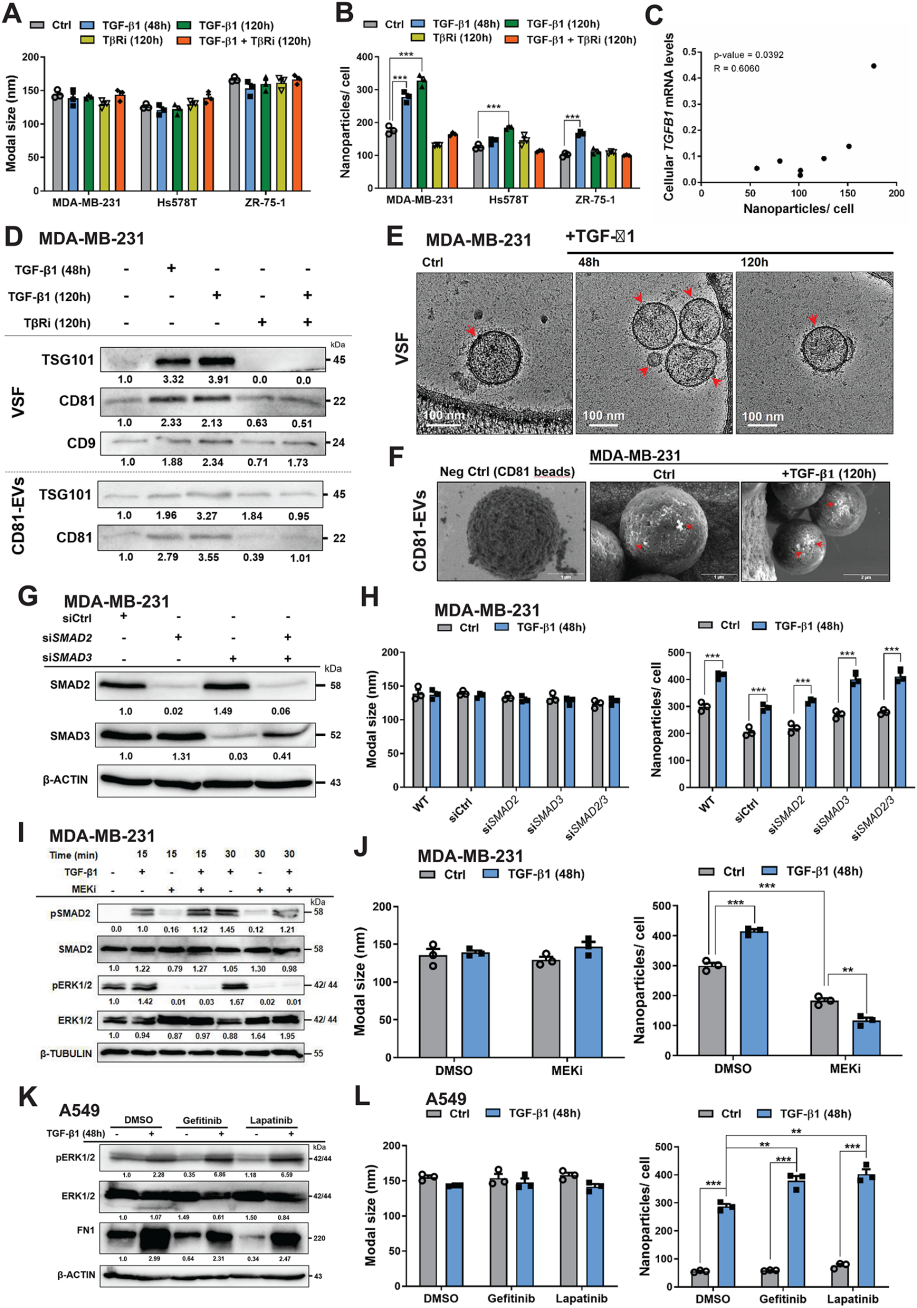
TGF-β induces EV secretion via activation of the MEK/ERK pathway. (**A**, **B**) EVs released by the three indicated cell models were quantified by NTA in terms of particle size (**A**) and particle number after normalization to the total cell number (**B**). The cells were stimulated with vehicle (Ctrl), 5 ng/mL TGF-β1 for 48–120 h, 5 µM LY2157299 TGF-β type I receptor inhibitor (TβRi) or combination of TGF-β with TβRi for 120 h. (**C**) Pearson correlation of nanoparticle numbers per cell with corresponding cellular *TGFB1* mRNA expression from the same cell. Each data point represents one of the seven cell lines analyzed. (**D**) Protein expression levels of the indicated EV-specific proteins in EV extracts (isolated as VSF or after CD81-specific enrichment) derived from MDA-MB-231 cells stimulated with 5 ng/mL TGF-β1 in the absence or presence of 5 µM TβRi for the indicated time periods, and densitometric values were normalized to the vehicle control. (**E**, **F**) Representative cryo-TEM (**E**) and SEM (**F**) pictures of EVs isolated as VSF (**E**) or CD81-enriched fraction (**F**) from MDA-MB-231 cells stimulated with 5 ng/mL TGF-β1 for 48–120 h or not (Ctrl). A negative control image of CD81-specific immunobeads alone is also shown (**F**). Scale bars are included and red arrows mark individual or clustered EVs. (**G**) Protein expression levels of SMAD2, SMAD3 and β-ACTIN (as loading control) in MDA-MB-231 protein extracts transiently transfected with the indicated siRNAs, and densitometric values were normalized to the control siRNA. (**H**) EVs released by MDA-MB-231 cells, which were first transiently transfected with SMAD-specific siRNAs, were quantified by NTA in terms of particle size (left) and particle number after normalization to the total cell number (right). The cells were stimulated with vehicle (Ctrl) or 5 ng/mL TGF-β1 for 48 h. (**I**) Protein expression levels of the indicated signaling proteins and β-TUBULIN (as loading control) in MDA-MB-231 cells stimulated with 5 ng/mL TGF-β1 in the absence or presence of 5 µM MEK inhibitor (MEKi; U0126) for the indicated time periods, and densitometric values were normalized to the vehicle control. (**J**) EVs released by MDA-MB-231 cells treated with vehicle (DMSO) or 5 µM MEKi and quantified by NTA in terms of particle size (left) and particle number after normalization to the total cell number (right). The cells were stimulated with vehicle (Ctrl) or 5 ng/mL TGF-β1 for 48 h. (**K**) Expression levels of the indicated proteins and β-ACTIN (as loading control) in A549 cells stimulated with 5 ng/mL TGF-β1 in the absence or presence of 5 µM gefitinib or 0.5 µM lapatinib for 48 h, and densitometric values normalized to the vehicle control. (**L**) EVs released by A549 cells treated with vehicle (DMSO) or 5 µM gefitinib or 0.5 µM lapatinib and quantified by NTA in terms of particle size (left) and particle number after normalization to the total cell number (right). The cells were stimulated with vehicle (Ctrl) or 5 ng/mL TGF-β1 for 48 h. Data in (**A**, **B**, **H**, **J** and **L**) are presented as mean values of three biological replicates ± SEM, each in technical duplicates and p-values are shown based on two-way ANOVA, followed by multiple paired comparisons conducted by means of Bonferroni’s post-test method. p-values: **p ≤ 0.01; ***p ≤ 0.001. The data in (**D**, **G**, **I** and **K**) show representative immunoblots of three independent biological replicates along with molecular mass markers in kDa

TGF-β preserves integrity of tissues and has different functions during cancer development depending on cancer type and stage [8]. KMplot analysis showed a correlation between high TGF-β1 protein levels and poor overall survival of BRCA patients (Supplementary Fig. S1A), consistent with the pro-tumorigenic role of TGF-β in BRCA. BRCAs are classified into several subtypes, including luminal-like BRCAs expressing estrogen receptor-α (ER) and/or progesterone receptor (PgR), and lacking expression of the human epidermal growth factor receptor 2 (HER2), HER2^+^ BRCAs and basal-like, HER2/ER/PgR-negative, known as triple-negative (TNBC) BRCAs [26]. We investigated the role of TGF-β signaling in the modulation of tumor-derived EVs using as model systems BRCA cell lines from distinct subtypes, the TNBC MDA-MB-231 and Hs578T, and the luminal-B ZR-75-1. Upon stimulation with TGF-β1 for 48 and 120 h, secreted EVs smaller than 200 nm were evaluated initially for their size and number through NTA (Supplementary Fig. S1B shows representative nanoparticle traces). Although no significant difference in the EV modal size was noted (Fig. 1A), TGF-β1 stimulation led to a significant increase in the number of EVs (EV^+ TGF−β1^) secreted by the three BRCA cells; concomitant treatment with a TGF-β receptor type I kinase inhibitor (TβRi, 2.0 µM) blocked the release of EV^+ TGF−β1^ (Fig. 1B). Similarly, TGF-β1 stimulation did not impact the modal size, but increased significantly the numbers of EV^+ TGF−β1^ released by A549 (LUAD), H1299 (non-small cell lung carcinoma), MCF7 (luminal-A BRCA), MCF10A (non-tumorigenic breast epithelial) and BNF3 (normal breast fibroblast) cells (Supplementary Fig. S1C), suggesting a general stimulatory effect of TGF-β on EV secretion by different tumor and non-tumorigenic cells. In line with this finding, there was a significant and positive correlation (*R* = 0.6060, *p* = 0.0392) between the mean expression of cellular *TGFB1* mRNA measured in each individual cell model (Supplementary Fig. S1D) and the mean number of nanoparticles released by each of the seven cell types examined (Fig. 1C), suggesting that autocrine TGF-β1 increases the titer of secreted EVs. It should be noted that the MCF7 cells were not included in this correlation analysis and the absolute number of nanoparticles monitored in each one and the same cell model could vary over experiments (Fig. 1, Supplementary Fig. S1). In contrast, the impact of TGF-β stimulation, biological or chemical perturbations (see below) always resulted in reproducible relative differences on nanoparticle numbers.

The EVs were isolated as three complementary pools, i.e. VSF covering many EV populations, CD81-EVs, enriched in the tetraspanin CD81, and cholerae toxin chain-B-selected EVs (CTB-EVs), enriched in GM1-gangliosides. Similar volume of collected MDA-MB-231 (MDA EVs), Hs578T, A549 and H1299 EV populations were characterized based on the presence of the EV markers TSG101, CD81, CD9, ALIX and β-ACTIN, whose increased levels upon TGF-β stimulation reflect the higher EV numbers contained in the same volume of EV preparation (Fig. 1D, Supplementary Fig. S1E-G). Additionally, MDA EVs were visualized by cryo-TEM demonstrating lipid bilayer continuity, particulate/filamentous luminal content, a rich surface decoration reminiscent of a glycocalyx and an apparent diameter of 60–140 nm, without obvious architectural or structural differences caused by TGF-β stimulation (Fig. 1E, Supplementary Fig. S1H). MDA CD81-EVs on immunobeads were characterized by SEM (Fig. 1F), whereas Hs579T, ZR-75-1, MCF7, MCF10A, A549 and H1299 EVs were visualized by TEM (Supplementary Fig. S1I), confirming relatively homogeneous populations of membrane-surrounded nanoparticles.

To identify endosomal compartments, MDA-MB-231 cells were incubated with bovine serum albumin (BSA)-Au particles, revealing that TGF-β stimulation did not alter the morphology of MVBs (Supplementary Fig. S2A). However, in the absence of 3D analysis of the ultrathin sectioned cells we were limited to accurately analyze MVB size or number of intraluminal vesicles. Moreover, we performed experiments of transferrin (TRF) uptake by MDA-MB-231 and ZR-75-1 cells, as measured by flow cytometry and direct fluorescence microscopy (Supplementary Fig. S2B-D). Since TGF-β1 stimulation did not affect TRF uptake it is unlikely that the higher release of EV^+ TGF−β1^ is due to higher rates of endosomal trafficking towards MVBs.

### MEK/ERK1/2 signaling mediates EV secretion in response to TGF-β

To better understand the molecular signaling driving EV^+ TGF−β1^ secretion, a time-course experiment was performed including 6, 24, 48 and 120 h of stimulation of MDA-MB-231 cells with TGF-β1. TGF-β1 stimulation for 24 h or longer was required to enhance release of EVs (Supplementary Fig. S2E), which had similar morphologies at all time points tested (Supplementary Fig. S2F). We scrutinized the kinetics of TGF-β-induced EV secretion by stimulating cells with TGF-β1 followed by addition of TβRi at different time points. TβRi addition 8 h post-TGF-β successfully abrogated EV^+ TGF−β1^ release, while blocking signaling 24 h after its onset was ineffective, as noted by NTA and ALIX, TSG101 and CD81 expression (Supplementary Fig. S2G, H). We conclude that enhanced secretion of EVs by TGF-β follows slow kinetics and is dependent on factors activated during the first 8 h of TGF-β signaling.

Silencing of SMAD2, SMAD3 or both in MDA-MB-231 cells (Fig. 1G, Supplementary Fig. S3A, B) followed by TGF-β1 stimulation for 48 h, did not block the number of EV^+ TGF−β1^ (Fig. 1H), suggesting that TGF-β-induced SMAD signaling may not be required to enhance EV release. The effectiveness of the SMAD2 and SMAD3 silencing was verified by analyzing the expression of established gene targets of TGF-β signaling, *MMP9* (*matrix metalloprotease 9*), whose induction by TGF-β1 was reduced after SMAD3 (but not SMAD2) silencing, and whose biological action will be scrutinized deeper later, and *TGFBI* (*TGF-β-induced*), whose induction by TGF-β was reduced after SMAD2 (but not SMAD3) silencing (Supplementary Fig. S3C, D). Thus, the absence of a clear SMAD2/3 impact on EV^+ TGF−β1^ secretion indicates that alternative non-SMAD signaling could be induced by TGF-β, such as the MEK/ERK1/2 pathway (see introduction). A MEK inhibitor (MEKi; U0126) that exhibited mild 0–20% toxicity in MDA-MB-231, ZR-75-1, A549 or embryonic HEK293T cells at 5 µM (Supplementary Fig. S4A), blocked basal and TGF-β1-stimulated ERK1/2 phosphorylation in MDA-MB-231 cells, without perturbing SMAD2 phosphorylation (Fig. 1I). MEKi decreased the number of MDA EVs maximally by 50%, whereas, TGF-β1 stimulation for 48 h was unable to induce any measurable EV secretion in the presence of MEKi (Fig. 1J). Similar findings were observed in ZR-75-1 and A549 cells treated with MEKi for 48 h (Supplementary Fig. S4B-E). It is worth noting that while the MEKi reduced basal levels of EV release in BRCA cells, it did not affect significantly the release of EVs by A549 cells (LUAD), whereas the negative impact of the MEKi on EV^+ TGF−β1^ secretion applied to all cell models tested. In order to monitor specificity of MEK/ERK1/2 activation downstream of TGF-β receptors, we asked whether EGF receptors that are well-established alternative activators of MEK/ERK1/2 signaling, could mediate the TGF-β effects on EV secretion. By employing two independent EGFR tyrosine kinase inhibitors, gefitinib or lapatinib, at concentrations that were not toxic to A549 and MDA-MB-231 cells (Supplementary Fig. S4F, G), we found that neither of the two chemicals inhibited ERK1/2 phosphorylation by TGF-β1 or TGF-β1-stimulated EV secretion (Fig. 1K, L, Supplementary Fig. S4H, I). On the other hand, fibronectin, whose protein expression in response to TGF-β is known to involve crosstalk between TGF-β and EGF signaling [27], was reduced partially when the two cell models were treated with the two different EGFR inhibitors (Fig. 1K, Supplementary Fig. S4H). Therefore, our data support the notion that TGF-β stimulation enhances the release of tumor-derived EVs, via sustained MEK/ERK1/2 and most likely additional late signaling mediators.

### TGF-β-induced EV release involves increased cholesterol accumulation

**Fig. 2:**
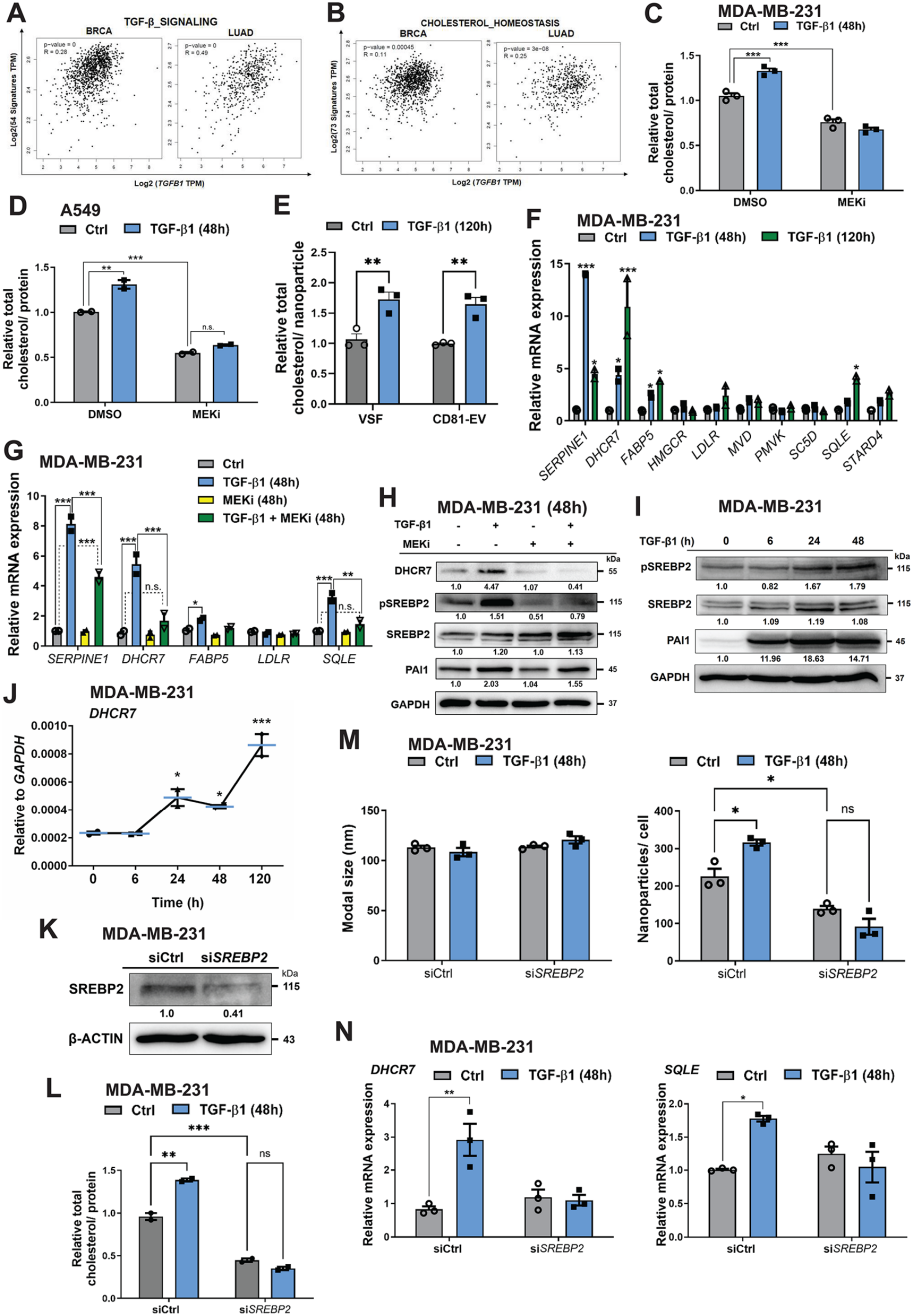
TGF-β induces cholesterol synthesis and ERK1/2-SREBP2 activation leading to DHCR7 expression. (**A**, **B**) Pearson correlation analysis of mRNA expression in BRCA and LUAD of gene signatures for TGF-β signaling (**A**) and cholesterol homeostasis (**B**) relative to the *TGFB1* mRNA expression, measured as transcripts per million (TPM) transformed by log_2_; data was obtained from TCGA. P- and R-values are listed. (**C**, **D**) Quantification of total cholesterol levels in MDA-MB-231 (**C**) and A549 (**D**) cells stimulated with 5 ng/mL TGF-β1 for 48 h in the absence or presence of 5 µM MEKi. (**E**) Quantification of total cholesterol levels in VSF and CD81-EVs enriched from MDA-MB-231 cells stimulated with 5 ng/mL TGF-β1 for 120 h. (**F**, **G**) RT-qPCR analysis of the indicated mRNA levels in MDA-MB-231 cells upon stimulation with 5 ng/mL TGF-β1 for 48–120 h (**F**) or in MDA-MB-231 cells stimulated with 5 ng/mL TGF-β1 in the absence or presence of 5 µM MEKi for 48 h (**G**). Values represent fold-change of mRNA expression normalized to *GAPDH* and expressed relative to the level at 0 h TGF-β1 (Ctrl). (**H**) Expression levels of the indicated cellular proteins and GAPDH serving as loading control from MDA-MB-231 cells treated as in panels C and G, and densitometric values were normalized to the vehicle control. (**I**) Expression levels of pSREBP2, SREBP2, PAI1 and GAPDH serving as loading control from MDA-MB-231 cells treated with TGF-β1 for 6, 24 and 48 h. Densitometric values were normalized to the vehicle control. (**J**) RT-qPCR analysis of *DHCR7* mRNA levels in MDA-MB-231 cells upon stimulation with 5 ng/mL TGF-β1 for the indicated time points. Values represent mRNA expression normalized to *GAPDH.* (**K**) Expression levels of SREBP2 protein with GAPDH serving as loading control in MDA-MB-231 cells transiently transfected with control (siCtrl) or specific siRNA targeting SREBP2 (siSREBP2). Densitometric values were normalized to the siCtrl. (**L**) Quantification of total cholesterol levels in MDA-MB-231 cells transiently transfected with control siRNA (siCtrl) or siSREBP2 and stimulated with 5 ng/mL TGF-β1 for 48 h. (**M**) EVs released by MDA-MB-231 cells, which were first transiently transfected with siCtrl or siSREBP2, were quantified by NTA in terms of particle size (left) and particle number after normalization to the total cell number (right). The cells were stimulated with vehicle (Ctrl) or 5 ng/mL TGF-β1 for 48 h. (**N**) RT-qPCR analysis of *DHCR7* and *SQLE* mRNA in MDA-MB-231 cells after transient transfection with siCtrl or siSREBP2 after stimulation of the transfected cells with 5 ng/mL TGF-β1 for 48 h. Values represent fold-change of mRNA expression normalized to *GAPDH* and expressed relative to the level at siCtrl. The cholesterol level data (**C**-**E**, **L**) and the RT-qPCR data (**F**, **G**, **K** and **N**) are presented as mean values of three biological replicates ± SEM, in technical triplicates and p-values are shown based on two-way ANOVA, followed by multiple paired comparisons conducted by means of Bonferroni’s post-test method: *p ≤ 0.05; **p ≤ 0.01; ***p ≤ 0.001. The protein data (**H**, **I** and **K**) show representative immunoblots of at least two independent biological replicates along with molecular mass markers in kDa

The human tumor datasets of TCGA were used to identify possible links between *TGFB1* mRNA expression and the signature of genes related to different biological pathways, including pathways that could orchestrate EV biogenesis, such as cholesterol homeostasis [12]. As expected, the gene set enrichment analysis (GSEA) of BRCA and LUAD datasets revealed positive and significant correlations between *TGFB1* mRNA expression and the signature of genes classified as hallmarks for TGF-β signaling and EMT (Fig. 2A, Supplementary Fig. S5A, Supplementary Table S1; note that the correlations were stronger in LUAD and reached levels of up to 0.54 but not higher). Using TGF-β signaling and EMT terms as positive controls helped us point our attention to the significant correlation between *TGFB1* levels and the gene signature of cholesterol homeostasis in both BRCA and LUAD datasets (Fig. 2B, Supplementary Table S1; note however the lower R values). Cholesterol is enriched in the membrane of microvesicles and exosomes and is one of the central contributors to EV biogenesis and release [12, 28]. This established knowledge stimulated further analysis in the direction of cholesterol biosynthesis. Furthermore, the significant correlation between *TGFB1* mRNA expression and the cholesterol homeostatic gene signature (Fig. 2B), is consistent with previous findings, outside the context of EV biogenesis, on regulation of expression of genes modulating cholesterol biosynthesis and distribution by TGF-β signaling [29, 30]. In line with these reports from non-cancerous cell types, we measured a significant increase of total cholesterol levels in MDA-MB-231, ZR-75-1 and A549 cells after 48 h of TGF-β1 stimulation (Fig. 2C, D; Supplementary Fig. S5B). Additionally, MEKi decreased the intracellular levels of cholesterol in MDA-MB-231 and A549 cells by 25–50% relative to the control, and abrogated completely the TGF-β1-induced increase in cholesterol level (Fig. 2C, D), indicating that the MEK/ERK1/2 pathway mediates both increase of cellular cholesterol levels and EV secretion by TGF-β1. Interestingly, TGF-β1 stimulation also enhanced significantly the total cholesterol content of EVs secreted by MDA-MB-231 and A549 cells (Fig. 2E; Supplementary Fig. S5C).

To uncover the mechanism of total cholesterol induction by TGF-β1, we assessed the mRNA expression of 9 out of the 73 genes from the GSEA cholesterol homeostasis gene signature, i.e. *DHCR7*, *FABP5*, *HMGCR*, *LDLR*, *MVD*, *PMVK*, *SC5D*, *SQLE* and *STARD4* (Supplementary Table S1, Supplementary Fig. S5D) in MDA-MB-231, ZR-75-1, MCF10A and A549 cells treated with TGF-β1 for 48 and 120 h (Fig. 2F; Supplementary Fig. S6A-C). *DHCR7* and *SQLE* were significantly induced by TGF-β1 in all breast epithelial cells, while *FABP5*, *HMGCR*, *LDLR*, *MVD*, *PMVK*, *SC5D* and *STARD4* were also increased after TGF-β1 stimulation, but in a cell type-dependent manner. Critically, TGF-β signaling required MEK/ERK1/2 activation in order to induce the expression of *DHCR7*, *FABP5* and *SQLE* in MDA-MB-231 and ZR-75-1 (Fig. 2G; Supplementary Fig. S6D) cells. DHCR7 protein analysis in MDA-MB-231 (Fig. 2H), ZR-75-1 and A549 (Supplementary Fig. S6E, F) cells validated the mRNA expression analysis.

The expression of genes involved in cholesterol synthesis, including *DHCR7* and *SQLE*, is regulated by the sterol regulatory element-binding protein-2 (SREBP2) [31], whose phosphorylation is induced by ERK1/2 [32]. Analysis of the time course of SREBP2 phosphorylation in response to TGF-β1 in MDA-MB-231 (Fig. 2I) and A549 (Supplementary Fig. S6F) cells, revealed accumulation of phospho-SREBP2 after TGF-β1 stimulation at 24 and 48 h, but not at 6 h. Importantly, the presence of MEKi successfully blocked the phosphorylation of SREBP2 in MDA-MB-231 (Fig. 2H) and A549 (Supplementary Fig. S6F) cells, suggesting that TGF-β induces the activation of SREBP2 via MEK/ERK1/2-mediated phosphorylation. Consistently, the time course of *DHCR7* mRNA expression in response to TGF-β stimulation showed late regulation (after 6 h; Fig. 2J), which matched well the induction of phosphorylation of SREBP2 and the kinetics of EV induction by TGF-β. Notably, SREBP2 silencing in MDA-MB-231 cells (Fig. 2K), decreased the total cholesterol levels and EV secretion (Fig. 2L, M), while in the absence of SREBP2, TGF-β1 stimulation failed to increase either cholesterol or EV secretion and to induce the expression of *DHCR7* and *SQLE* (Fig. 2L-N), without affecting *SERPINE1* induction (Supplementary Fig. S6G). Note that *SERPINE1*, also known as plasminogen activator inhibitor 1 (PAI1; Fig. 2F-I; Supplementary Fig. S6A-D, F-H), whose transcription is induced after formation of a p53/TFE3/Sp1/SMAD complex bound to the *SERPINE1* promoter in response to TGF-β [33–35], was used as a positive control for TGF-β signaling activation. Moreover, we silenced SMAD2, SMAD3 or both (efficiency of protein silencing shown in Supplementary Fig. S6I). SMAD2 or SMAD2/3 silencing did not perturb the TGF-β-induced expression of *DHCR7* and *SQLE* in MDA-MB-231 cells, whereas SMAD3 silencing led to a strong decrease in their basal expression (Supplementary Fig. S6J). It should not go unnoticed that TGF-β modulated *LDLR* mRNA expression, which appeared to be context-dependent, as its levels were enhanced only in MCF10A but not in the tumor cells (Fig. 2F, G; Supplementary Fig. S6A-D). SMAD-specific interference also failed to provide convincing evidence for a transcriptional input to *LDLR* gene regulation (Supplementary Fig. S6J). We conclude that a TGF-β receptor to MEK/ERK1/2 to SREBP2 signaling cascade leads to induction of cholesterol biosynthesis and secreted EV accumulation.

### DHCR7 contributes to EV release in response to TGF-β

The expression of the TGF-β-regulated genes (*DHCR7*, *FABP5*, *HMGCR*, *MVD*, *SQLE* and *STARD4*) was further correlated with clinical features using cancer patient datasets (Supplementary Fig. S7-S9). Among these genes, only *DHCR7* was highly expressed in both BRCA and LUAD patients in comparison to healthy controls (Supplementary Fig. S7), and its higher expression significantly correlated with (i) the poor overall survival of BRCA (Supplementary Fig. S8A) and lung cancer (Supplementary Fig. S9A) patients, and (ii) with the lack of response to chemotherapies in BRCA patients (Supplementary Fig. S8B). Therefore, the cumulative data pointed to the clinical relevance of DHCR7, the rate-limiting enzyme that catalyzes the conversion of 7-dehydrodesmosterol to desmosterol in the Bloch pathway and the conversion of 7-dehydrocholesterol to cholesterol in the Kandutsch-Russell pathway (Supplementary Fig. S5D) [36].

**Fig. 3:**
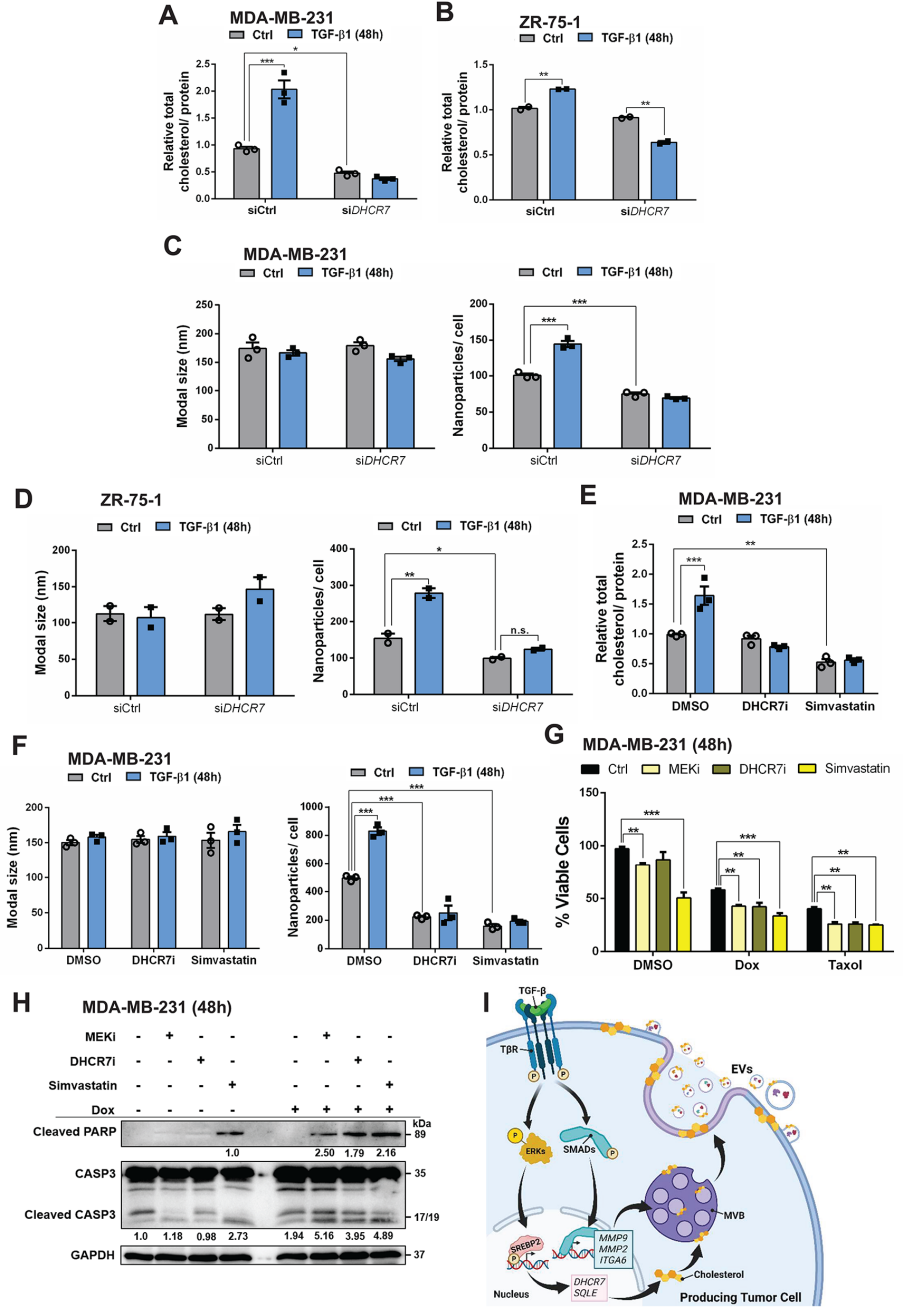
TGF-β induces EV release via DHCR7 and the cholesterol pathway. (**A**, **B**) Quantification of total cholesterol levels in MDA-MB-231 (**A**) and ZR-75-1 (**B**) cells stimulated with 5 ng/mL TGF-β1 for 48 h after transient transfection with control or siRNA targeting *DHCR7*. (**C**, **D**) EVs released by MDA-MB-231 (**C**) and ZR-75-1 (**D**) cells transiently transfected with control or siRNA targeting *DHCR7*, quantified by NTA in terms of particle size (left) and particle number after normalization to the total cell number (right). The cells were stimulated with vehicle (Ctrl) or 5 ng/mL TGF-β1 for 48 h. (**E**) Quantification of total cholesterol levels in MDA-MB-231 cells stimulated with 5 ng/mL TGF-β1 for 48 h in the absence or presence of 20 µM DHCR7i (AY9944) or 1.25 µM simvastatin. (**F**) EVs released by the MDA-MB-231 cells quantified by NTA in terms of particle size (left) and particle number after normalization to the total cell number (right). The cells were stimulated with vehicle (Ctrl) or 5 ng/mL TGF-β1 in the absence or presence of 20 µM DHCR7i or 1.25 µM simvastatin for 48 h. (**G**) Cell viability assay with MDA-MB-231 cells incubated with vehicle DMSO (Ctrl), 5 µM MEKi, 20 µM DHCR7i or 1.25 µM simvastatin for 48 h, in the presence of co-incubation with DMSO, 0.5 µM Dox or 0.25 µM Taxol. (**H**) Representative immunoblot of at least two independent biological replicates of cleaved PARP1 and Caspase-3 (CASP3) in MDA-MB-231 cells treated with 5 µM MEKi, 20 µM DHCR7i or 1.25 µM simvastatin for 48 h, in the presence of DMSO or 0.5 µM Dox. GAPDH was used as a loading control, and molecular mass (kDa) markers are indicated along with densitometric values of normalized band intensity only in the lanes where the relevant protein markers scored positively. (**I**) Schematic model created with Biorender.com, summarizing the effect of TGF-β signaling on EV secretion. TGF-β from the extracellular matrix binds to its receptors (TβR) on the plasma membrane activating phosphorylation-dependent (**p**) SMAD and ERK signaling. In the nucleus, ERK induces SREBP2 phosphorylation, which enhances expression of cholesterol biosynthesis genes (*DHCR7*, *SQLE*). SMAD signaling induces invasion and adhesion genes (*MMP2*, *MMP9*, *ITGA6*). The cholesterol synthesis genes lead to cholesterol increase, causing EV secretion as shown in this paper. In addition to EV secretion (invaginated cell membrane), microvesicles are shown to emanate from the cell surface. The cholesterol level data (**A**, **B** and **E**), EV/NTA data (**C**, **D** and **F**) and cell viability (**G**) are presented as mean values of three biological replicates ± SEM, in technical triplicates and p-values are shown based on two-way ANOVA, followed by multiple paired comparisons conducted by means of Bonferroni’s post-test method: *p ≤ 0.05; **p ≤ 0.01; ***p ≤ 0.001

We investigated whether DHCR7 regulation could be a central mechanism by which TGF-β signaling increases cholesterol levels and EV^+ TGF−β1^ secretion. Silencing of DHCR7 in MDA-MB-231 (Supplementary Fig. S10A, B; siDHCR7_C being the only siRNA decreasing DHCR7 expression effectively) and ZR-75-1 (Supplementary Fig. S10C) cells, led to a 50% decrease of total cholesterol levels in MDA-MB-231 in the absence of TGF-β (Fig. 3A), whereas no significant reduction in basal total cholesterol level was seen in ZR-75-1 cells (Fig. 3B). Upon stimulation with TGF-β1, total cholesterol levels remained low and suppressed in both cell models (Fig. 3A, B). Importantly, DHCR7 silencing decreased the number of EVs secreted from MDA-MB-231 and ZR-75-1 cells by 20% and 50% respectively (Fig. 3C, D), confirming the established role of cholesterol on EV biogenesis. Notably, after DHCR7 silencing, TGF-β1 stimulation did not increase the EV levels (Fig. 3C, D).

In order to validate independently the importance of DHCR7 silencing in BRCA cells, a DHCR7 chemical inhibitor (DHCR7i; AY9944) was employed at a non-toxic IC_10_ concentration (20 µM) (Supplementary Fig. S10D). In contrast to DHCR7 knockdown, DHCR7i did not reduce the total cholesterol levels, although it efficiently blocked the cholesterol induced by TGF-β in MDA-MB-231 cells (Fig. 3E). Furthermore, treatment with DHCR7i decreased the basal numbers of EVs released by 50% and TGF-β1 stimulation failed to enhance EV release in either of the BRCA cells (Fig. 3F, Supplementary Fig. S10E). Finally, we administered simvastatin, an inhibitor of HMG-CoA reductase (HMGCR), another rate-limiting enzyme that initiates cholesterol biosynthesis (Supplementary Fig. S5D; cytotoxic response shown in Supplementary Fig. S10F). Simvastatin at 2.5 µM effectively decreased the total amount of cholesterol (Supplementary Fig. S10G), and blocked completely the induction of total cholesterol by TGF-β1 in MDA-MB-231 cells (Fig. 3E). Moreover, simvastatin decreased the number of EVs secreted by MDA-MB-231 cells by 60% and prevented any TGF-β-mediated induction of EV^+ TGF−β1^ release, phenocopying the DHCR7i (Fig. 3F). Simvastatin had a similar effect on EV secretion in the LUAD A549 cells but did not neutralize the effect of TGF-β1 (Supplementary Fig. S10H).

Although the primary effect of TGF-β signaling characterized in this study is the enhanced production of EVs, we also sought to explore possible effects of the compounds that blocked the release of EV^+ TGF−β1^ from tumor cells in the context of doxorubicin (Dox) or paclitaxel (taxol) cytotoxicity. Of note, resistance to anticancer drugs is facilitated by complex mechanisms, including TGF-β, EMT and tumor-derived EVs [6, 8]. Thus, upon examining cytotoxicity to Dox and taxol via dose-response analysis (Supplementary Fig. S10I, J), MDA-MB-231, ZR-75-1 and A549 cells were treated for 48 h in the absence or presence of MEKi, DHCR7i or simvastatin (Fig. 3G, Supplementary Fig. S10K, L). The combination of inhibitors with the cytotoxic drugs led to a significant decrease in tumor cell viability when compared to the chemotherapeutic treatment in the absence of the MEK and DHCR7 inhibitors (Fig. 3G, Supplementary Fig. S10K, L). Cytotoxicity correlated to higher levels of cleaved PARP and caspase3 in MDA-MB-231 cells treated with Dox, suggesting that cell death was mediated by apoptosis (Fig. 3H). These data show that inhibitors of TGF-β-induced EV secretion potentiate the cytotoxic effect of chemotherapeutic drugs. Altogether, our data suggest that, mechanistically, TGF-β1 activates MEK/ERK1/2 signaling, which in turn phosphorylates SREBP2 and enhances expression of enzymes involved in cholesterol synthesis, including DHCR7, which positively regulates tumor-derived EV release (Fig. 3I). Thus, regulation of DHCR7 expression and cholesterol elevation provide the intermediate link for the long-term effect that TGF-β signaling exhibits in regulating EV release.

### TGF-β signaling modulates a subset of the EV cargo proteome

**Fig. 4:**
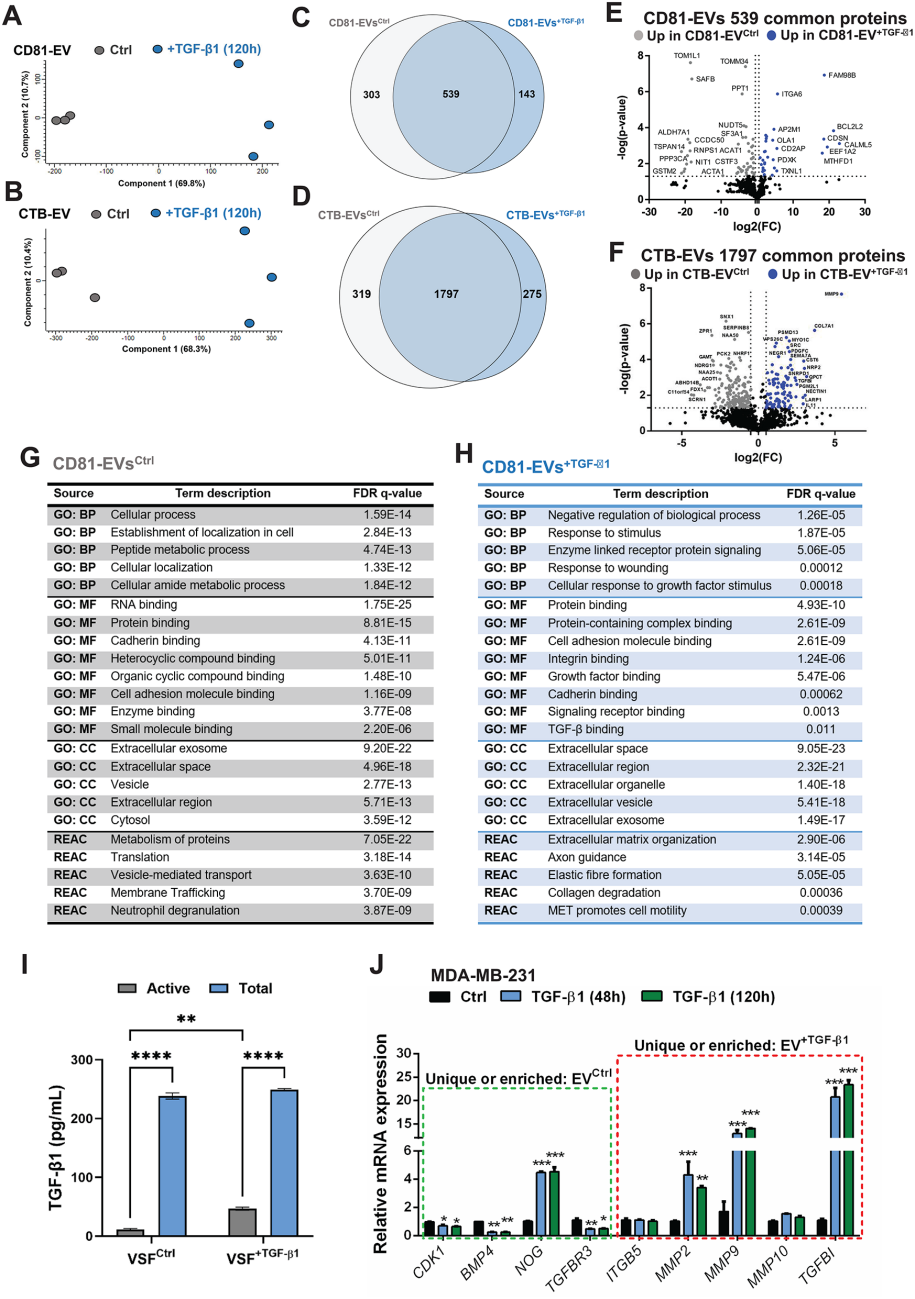
Analysis of the EV cargo proteome. (**A**, **B**) PCA of statistically significant protein expression differences between EVs enriched by the CD81-specific (**A**) or the CTB-specific (**B**) method in the supernatant of MDA-MB-231 cells stimulated with vehicle (Ctrl) or 5 ng/mL TGF-β1 for 120 h. Three independent biological repeats were analyzed per condition. (**C**, **D**) Venn diagram summarizing the total number of significant and differentially expressed proteins in at least two biological conditions shown in panel **A** and **B**. (**E**, **F**) Volcano plot of the significant (log_2_(p-value); y-axis) and differentially expressed (log_2_(fold-change/FC); x-axis) common proteins between the same two biological conditions shown in panels **A**-**D** respectively, expressed as proteins enriched (Up) in the EVs of control or TGF-β1-stimulated cells. Selected protein IDs are shown. Dotted lines indicate the filtering levels along each axis. (**G**, **H**) Tables of highly significant gene ontology (GO) and Reactome (REAC) terms represented in the two biological conditions analyzed using the CD81-specific EV isolation method, indicating the term name and associated false discovery rate (FDR) q-value. (**I**) RT-qPCR analysis of the indicated mRNAs, selected based on the corresponding proteins that scored significantly in the proteomic analysis of MDA-MB-231 cells after stimulation with 5 ng/mL TGF-β1 for 0 (Ctrl), 48 and 120 h. (**J**) Active and total TGF-β1 concentration was measured through ELISA-sandwich in EVs derived from control cells (VSF^Ctrl^) or cells stimulated with 5 ng/mL TGF-β1 for 120 h (VSF^+ TGF−β1^). TGF-β1 concentrations are presented based on two-way ANOVA, followed by multiple paired comparisons conducted by means of Bonferroni’s post-test method: **p ≤ 0.01; ****p ≤ 0.0001

We investigated whether TGF-β could also change constituents of the EV protein cargo. To this end, the total protein content of isolated CD81-EVs and CTB-EVs from MDA-MB-231 cells was subjected to LC-ESI-MS/MS. PCA revealed that both CD81-EV^+ TGF−β1^ and CTB-EV^+ TGF−β1^ acquired different profiles of protein content after 120 h of TGF-β1 stimulation, compared to EV^Ctrl^ (Fig. 4A, B). Moreover, the protein content differed among CD81-EV and CTB-EV subpopulations secreted by MDA-MB-231 cells under the same condition (Supplementary Fig. S11A, B). From the 985 proteins detected in the subpopulation of CD81-EVs, 303 were unique in EV^Ctrl^, while 143 proteins were specific in EV^+ TGF−β1^ (Fig. 4C). Additionally, 2,392 proteins were detected in the CTB-EV fraction, of which 319 proteins were exclusive to EV^Ctrl^ and 275 were unique to EV^+ TGF−β1^ (Fig. 4D). Lists of unique proteins in CD81-EVs and CTB-EVs are shown in Supplementary Tables S6 and S7, respectively. The common proteins carried by CD81-EV (*n* = 539, 54.72%) and CTB-EV (*n* = 1,797, 75.12%) fractions are shown as volcano plots (Fig. 4E, F). Besides the expected enrichment of “extracellular vesicle/exosome”-related terms, these proteins were significantly clustered into key pathways using the STRING algorithm, based on Gene Ontology (GO) of Cellular Component (CC), Biological Process (BP), Molecular Function (MF) and Reactome (REAC) terms. Associations to “cellular process”, “protein/RNA binding”, “cell adhesion molecule”, “cadherin binding”, “infectious disease” and “immune system” were found in both EV subpopulations (Supplementary Fig. S11C, D), indicating a possible redundant function transferred by different tumor-derived EVs to recipient cells.

The unique proteins identified in the CD81- and CTB-EV cargoes of the EV^Ctrl^ and EV^+ TGF−β1^ groups shared analogous CC terms relevant for EV networks (Fig. 4G, H, Supplementary Fig. S11E, F). However, despite the fact that identical MF terms were found in CD81-EVs, such as cadherin and cell adhesion molecule binding, it was evident that CD81-EV^+ TGF−β1^ were enriched in MF terms associated with TGF-β signaling (e.g., “growth factor binding”, “signaling receptor binding” and “TGF-β binding”), including the BP terms, “response to wounding and cellular response to growth factor stimulus”, when compared to CD81-EV^Ctrl^ (Fig. 4G, H). Similarly, CTB-EV^+ TGF−β1^ were enriched in BP terms associated with TGF-β signaling, such as “TGF-β receptor signaling pathway”, “cellular response to TGF-β stimulus”, “response to growth factor”, “cellular response to growth factor stimulus” and “transmembrane receptor serine/threonine kinase signaling” (Supplementary Fig. S11E, F). Both CD81-EV^+ TGF−β1^ and CTB-EV^+ TGF−β1^ protein profiles were enriched in REAC terms of “extracellular matrix organization”, “elastic fiber formation” and “collagen degradation” (Fig. 4H, Supplementary Fig. S11F). Furthermore, key proteins of the unique groups belonged to GO terms also shared by the common groups. The latter terms of “extracellular exosome”, “extracellular vesicle” and “extracellular region” included one of the highest top-hit proteins, MMP9 (Fig. 4F and see further analysis below).

Of note, TGF-β1 and TGF-β2 were detected in both CD81- and CTB-EV subpopulations, but without significant differences among the groups and below the cut-off thresholds of the volcano plots (Fig. 4E, F). Thus, we validated our MS analysis, confirming by ELISA that TGF-β1 protein is carried by EVs without significant differences among the total TGF-β1 carried by Ctrl or TGF-β-induced EVs (mean average of 243,74 pg/mL, equivalent to 1 × 10^9^ nanoparticles) (Fig. 4I). Nevertheless, it is important to mention that the levels of active TGF-β1 carried by EV^+ TGF−β1^ were significantly higher compared to EV^Ctrl^, even though the majority of TGF-β1 associated to EVs is present in the latent complex (Fig. 4I). Altogether, the cumulative proteomic data confirm that activation of TGF-β signaling can modify the sorting of protein cargo of tumor-derived EVs and that TGF-β signaling and extracellular matrix regulators, such as MMPs, are important constituents of such tumor-derived EVs.

Next, we sought to understand better the loading of protein cargo found uniquely or enriched into different EV fractions by comparing the presence of specific EV-associated proteins with the respective cellular mRNA expression upon TGF-β stimulation for 48 and 120 h in the MDA-MB-231 and ZR-75-1 cells (Fig. 4J, Supplementary Fig. S12A). Four genes *CDK1*, *BMP4*, *NOG* and *TGFBR3*, whose proteins were more abundantly enriched in EV^Ctrl^, and five genes, *ITGB5*, *MMP2*, *MMP9*, *MMP10* and *TGFBI*, whose proteins were more abundantly enriched in EV^+ TGF−β1^, were selected. Among these are genes encoding the TGF-β co-receptor TGFBR3/β-glycan, the latent TGF-β-activating enzymes MMP2 and MMP9 and TGFBI, the collagen-associated protein of the extracellular matrix that is a highly responsive gene to TGF-β signaling, as described above. MDA-MB-231 and ZR-75-1 cells stimulated with TGF-β1 presented a significant reduction of *CDK1*, *BMP4* and *TGFBR3* expression, and at the same time, *MMP2*, *MMP9* and *TGFBI* were significantly enhanced. Of importance, not all the EV protein cargo reflected the transcriptional effects induced by TGF-β, as noted for *NOG*, *ITGB5* and *MMP10*, suggesting that sorting of different cargo proteins into tumor-derived EVs does not strictly follow the abundance of the respective mRNA.

One of the common proteins between EV^Ctrl^ and EV^+ TGF−β1^, which was also highly enriched in EV^+ TGF−β1^ was MMP9 (Fig. 4C, F, Supplementary Table S6), a matrix metallopeptidase highly expressed in BRCA and LUAD compared to non-tumorigenic samples (Supplementary Fig. S12B). Moreover, *MMP9* mRNA expression correlated with poor overall survival of TNBC and LUAD patients (Supplementary Fig. S12C, D), but did not correlate with chemotherapy response in TNBC patients (Supplementary Fig. S12E). The relevance of *MMP9* mRNA expression in predicting TNBC and LUAD patient outcome, suggests that EV-associated MMP9 could possibly identify circulating EVs in cancer patients. In addition, the co-presence of MMP9 and latent TGF-β1 (Fig. 4I) as cargo of EVs may be linked to the mechanism of activation of latent TGF-β carried by EVs [7]. In general, the proteomic profile revealed that MDA-MB-231-derived EVs carry a complexity of signaling molecules which could deliver simultaneously actionable pathways in recipient cells.

### EV internalization is not required to activate TGF-β signaling

**Fig. 5:**
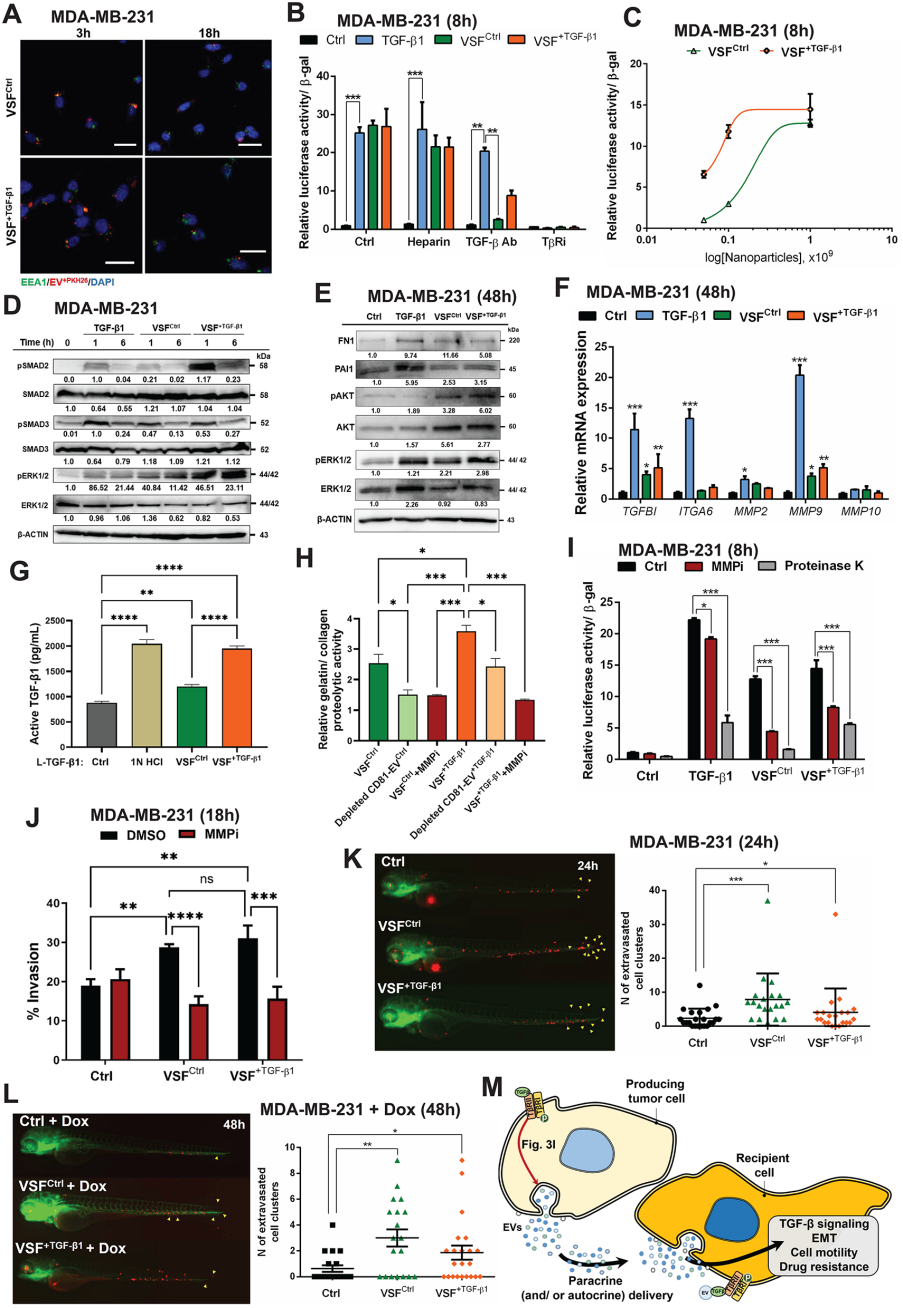
EV cargo MMPs and TGF-β activate TGF-β signaling in recipient cancer cells. (**A**) Representative immunofluorescence microscopy pictures of MDA-MB-231 cells incubated with EVs derived from control cells (VSF^Ctrl^) or cells stimulated with 5 ng/mL TGF-β1 for 120 h (VSF^+ TGF−β1^) for 3–18 h. The early endosome protein EEA1 (green), the EVs (red) and nuclei (blue) are labeled. (**B**) Relative luciferase activity generated in MDA-MB-231 cells by transfecting the TGF-β-inducible CAGA_12_-luc reporter, normalized to β-galactosidase activity generated by a co-transfected reporter, after stimulation of the transfected cells with vehicle (Ctrl), 5 ng/mL TGF-β1, or incubation with 1 × 10^9^ nanoparticles per mL of VSF^Ctrl^ or VSF^+ TGF−β1^ for 8 h. The cells were also treated with vehicle (Ctrl), 50 µg/ml heparin, 5 µg/mL neutralizing anti-TGF-β antibody or 5 µM TβRi. Data are presented as mean values of three biological replicates ± SEM, each in technical duplicates. (**C**) Relative CAGA_12_-luciferase activity generated in MDA-MB-231 cells after incubation of the transfected cells for 8 h with increasing nanoparticle numbers per mL (5 × 10^7^, 1 × 10^8^ and 1 × 10^9^) of VSF^Ctrl^ or VSF^+ TGF−β1^. Data are presented as mean values of three biological replicates ± SEM, each in technical duplicates. (**D**, **E**) Protein expression levels of the indicated signaling proteins in cellular extracts of MDA-MB-231 cells stimulated or not with 5 ng/mL TGF-β1 or incubated with 1 × 10^9^ nanoparticles per mL of VSF^Ctrl^ or VSF^+ TGF−β1^ for the indicated time periods, and densitometric values normalized to the control 0 h. Representative immunoblots of three independent biological replicates along with molecular mass markers in kDa are shown. (**F**) RT-qPCR analysis of the indicated mRNA levels in MDA-MB-231 cells stimulated with vehicle (Ctrl) or 5 ng/mL TGF-β1, or incubated with 1 × 10^9^ nanoparticles per mL of VSF^Ctrl^ or VSF^+ TGF−β1^ for 48 h. The data are presented as mean values of three biological replicates ± SEM, in technical triplicates. (**G**) Quantification of active TGF-β1 released from a constant amount (2.5 ng) latent-(**L**)-TGF-β1 was measured through ELISA-sandwich upon acidification with 1 N HCl for 10 min at room temperature or after incubation for 30 min at room temperature with 1 × 10^9^ nanoparticles per mL of VSF^Ctrl^ or VSF^+ TGF−β1^. Note that 2.5 ng L-TGF-β1 contained a portion of mature TGF-β1 (Ctrl). (**H**) MMP proteolytic activity was measured in 1 × 10^9^ nanoparticles per mL from VSF^Ctrl^ or VSF^+ TGF−β1^ using EnzChek Gelatinase/Collagenase Assay kit. The VSFs were pre-treated with vehicle DMSO (Ctrl), 25 µM MMP inhibitor (MMPi) or depleted from CD81-positive EVs. (**I**) Relative luciferase activity generated in MDA-MB-231 cells by transfecting the TGF-β-inducible CAGA_12_-luc reporter, normalized to β-galactosidase activity generated by a co-transfected reporter, after stimulation of the transfected cells with vehicle (Ctrl), 5 ng/mL TGF-β1, or incubation with 1 × 10^9^ nanoparticles per mL of VSF^Ctrl^ or VSF^+ TGF−β1^ for 8 h. The VSFs were pre-treated with vehicle DMSO (Ctrl), 25 µM MMPi or 40 µg/ml proteinase K for 1 h. (**J**) Matrigel invasion assay in trans-wells with MDA-MB-231 cells stimulated with vehicle (Ctrl) or incubated with 1 × 10^9^ nanoparticles per mL of EVs derived from control cells (VSF^Ctrl^) or cells stimulated with 5 ng/mL TGF-β1 (VSF^+ TGF−β1^) for 48 h in the presence of vehicle (DMSO) or 25 µM MMP inhibitor (MMPi). (**K**, **L**) In vivo extravasation and collagenous tail-fin invasion assay in zebrafish embryos injected with fluorescently labelled (red) MDA-MB-231 cells stimulated with vehicle (Ctrl) or incubated with 1 × 10^9^ nanoparticles per mL of VSF^Ctrl^ or VSF^+ TGF−β1^ for 48 h prior to their microinjection in the duct of Cuvier of transgenic zebrafish with GFP-tagged (green) vasculature. Microinjected embryos were then incubated in water in the absence (**K**) or presence (**L**) of 5 µM Dox. Images were captured 24 h after microinjection. The data in K and L represent the extravasated cells that invaded the collagenous tail-fin cells as numbers of red fluorescent cell clusters and are plotted as mean values of at least 20 biological replicates (individual embryos) ± SEM and p-values are shown based on Wilcoxon matched-pairs test. Comparisons in panels **B**, **C**, **F**-**J** are presented as mean values of at least two biological replicates ± SEM. The p-values in **B**, **F**, **I** and **J** are based on two-way ANOVA, followed by multiple paired comparisons conducted by means of Bonferroni’s post-test method, while in G and H, each in technical triplicates, are shown based on one-way ANOVA, followed by multiple paired comparisons conducted by means of Bonferroni’s post-test method: *p ≤ 0.05; **p ≤ 0.01; ***p ≤ 0.001; **** *p* ≤ 0.0001. (**M**) Schematic model summarizing the effect of TGF-β signaling on higher EV secretion, assisting tumorigenesis. TGF-β signaling activation enhances EV secretion as summarized in Fig. 3I. TGF-β signaling activated by EVs does not require heparan sulphate proteoglycan-mediated internalization, suggesting that the EV TGF-β signals directly at the cell surface. Secreted EVs act in an autocrine or paracrine manner on tumor cells or other cells in the tumor microenvironment, inducing multiple phenotypes: TGF-β signaling, EMT, cell motility and viability that contributes to resistance of cancer cells to cytotoxic drugs

To examine the potential for intercellular communication mediated by MDA EVs, we first characterized the routes of EV interaction with recipient cells by labelling the EV membranes with the lipid dye PKH26 [37]. No significant difference was found in the modal size of fluorescent MDA EVs, suggesting lack of aggregation after labelling (Supplementary Fig. S13A). We then monitored the internalization of fluorescent MDA EVs to recipient MDA-MB-231 cells (homotypic transfer) or to MCF7 and MCF10A (heterotypic transfer) immunostained for EEA1, a scaffold protein of early endosomes. At the earlier time point (1–3 h), only a fraction of fluorescent MDA EV^Ctrl^ and EV^+ TGF−β1^ co-localized with EEA1, suggesting endocytic uptake (Fig. 5A; Supplementary Fig. S13B, C), while additional cellular compartments appeared to interact with fluorescent MDA EVs, which suggests either membrane fusion, ligand-receptor interaction or endosomal escape. At the later time point of 18 h, a dramatic decrease of fluorescent MDA EVs was observed in the cells, suggesting that EVs were taken up at the early time points and were then processed intracellularly, leading to the loss of the lipophilic PKH26 label (Fig. 5A; Supplementary Fig. S13B, C). Additionally, the use of heparin (50 µg/mL), shown previously to prevent EV uptake by recipient cells [38], successfully decreased the uptake of fluorescent MDA EVs not only in recipient MDA-MB-231 cells, but also in ZR-75-1 and MCF10A cells (Supplementary Fig. S13D-F).

We monitored quantitatively whether similar numbers of MDA EV^Ctrl^ or EV^+ TGF−β1^ (1 × 10^9^ nanoparticles) could activate TGF-β signaling. This was determined by the CAGA_12_-luciferase reporter, which is highly specific and monitors only SMAD transcriptional activity, in other words, TGF-β family-specific signaling [24]. The EVs induced TGF-β signaling in recipient MDA-MB-231 cells after 8 h incubation, while depletion of the CD81-positive EV population reduced the effect of EVs on TGF-β signaling by approximately 30%, suggesting that approximately one third of the measured response depends on CD81-specific EV subpopulations (Fig. 5B, Supplementary Fig. S14A). Of note, although no significant differences were observed between EV^Ctrl^ and EV^+ TGF−β1^ when 10^9^ nanoparticles per mL were used, dose-dependent experiments revealed a higher potency of the EV^+ TGF−β1^ to activate the reporter under non-saturating conditions (0.05–0.5 × 10^9^ nanoparticles per mL; Fig. 5C). Furthermore, when EV uptake was blocked by heparin, MDA EVs still activated the CAGA_12_-luciferase reporter (Fig. 5B). On the other hand, TGF-β signaling induced by MDA EVs was significantly decreased when the EVs were pre-incubated with a neutralizing monoclonal antibody against TGF-β1/2/3 (Fig. 5B); the anti-TGF-β Ab concentration used (5 µg/mL) was sufficient to block effectively up to 1 ng/mL of recombinant mature TGF-β1 (Supplementary Fig. S14B) and almost completely 5 ng/mL (Fig. 5B). As expected, the presence of TβRi completely blocked CAGA_12_-luciferase reporter activity under all conditions (Fig. 5B). Moreover, upon 8 h treatment, MDA EVs also induced TGF-β signaling in MCF10A cells (Supplementary Fig. S14C). Additionally, the phosphorylation levels of SMAD2, SMAD3 and ERK1/2 were increased after 1 and 6 h of EV interaction with recipient cells (Fig. 5D). Monitoring at a later time point, 48 h, increased levels of FN1, PAI1, pERK1/2 and pAKT were determined by immunofluorescence microscopy (Supplementary Fig. S14D, E; FN1, pERK1/2) and immunoblotting (Fig. 5E, all markers, Supplementary Fig. S14F; FN1, PAI1, pERK1/2) in recipient MDA-MB-231 and ZR-75-1 cells treated with MDA EVs. Additionally, MDA EVs significantly enhanced *TGFBI* and *MMP9* mRNA expression in MDA-MB-231 (Fig. 5F). Depletion of the CD81-positive EV population reduced significantly the TGF-β signaling response of these two genes, demonstrating via this independent assay that CD81-specific EV subpopulations contributed to these gene responses (Supplementary Fig. S14G). Similarly, in MCF7, BNF3 and BNF2 cells, MDA EVs induced *MMP2*, *MMP9*, *MMP10* and *MMP14* expression (Supplementary Fig. S14H-J). As a word of caution, we emphasize that the original TGF-β stimulus provided to cancer cells in all experiments, which led to increased EV secretion, cannot be directly extrapolated to phenocopy the target cell signaling initiated by latent TGF-β carried as cargo by these EVs. As exemplified by out signaling analyses, comparison of pSMAD2 and pSMAD3 versus pERK1/2 and pAKT levels measured in recipient MDA-MB-231 and ZR-75-1 cells, indicated that pERK1/2 and pAKT were induced by MDA EVs to a much stronger degree than by TGF-β (Fig. 5D, E, Supplementary Fig. S14E, F). This suggests that other signals carried by MDA EVs promoted the activation of these pathways in parallel to the EV cargo of latent TGF-β.

The above data uncover that MDA EVs do activate TGF-β signaling and this signal does not require EV internalization by the recipient cells. Instead, EV interactions with cell surface TGF-β receptors might be sufficient to drive signaling, being accompanied by parallel pathways not described here in depth that are simultaneously activated upon interaction of EVs with the recipient cells.

### EV MMPs and surface proteins increase TGF-β signaling and invasive phenotype

We investigated further the requirement for EV cargo TGF-β, MMP and other proteins to mechanistically induce TGF-β signaling responses in recipient cells. As pointed out by our MS analysis (Fig. 4), EV^+ TGF−β1^ carried higher levels of MMP2, MMP9 and integrins, which are known molecules promoting the release of the active TGF-β dimer from its latent complex [39, 40]. This could explain partially the significantly higher levels of active TGF-β1 monitored in EV^+ TGF−β1^ (Fig. 4I). In order to demonstrate the endogenous capacity of EVs and associated proteins to activate the latent form of TGF-β, a constant amount (2.5 ng) of recombinant latent-TGF-β1 was either activated by acidification with 1 N HCl for 10 min or was treated with similar numbers of MDA EVs (1 × 10^9^ nanoparticles) for 30 min at room temperature. Notably, both VSF^Ctrl^ and VSF^+ TGF−β1^ induced a significant release of TGF-β1 from their respective recombinant latent complex pools, and EV^+ TGF−β1^ exerted such activation in a more efficient manner (2.23-fold increase in activation) than EV^Ctrl^ (1.47-fold increase in activation), and as efficiently as acidification (2.4-fold increase in activation) (Fig. 5G), presumably due to cargo or associated proteins enriched in EV^+ TGF−β1^.

To assess the functionality of MMPs associated with EVs, we measured the gelatin/collagen proteolytic activity of EVs before or after depletion of the CD81-positive EV populations, serving as specificity control. As shown in Fig. 5H, similar numbers of MDA EVs (1 × 10^9^ nanoparticles) enhanced matrix lysis in comparison to EV-free media (used as reference); depletion of the CD81-positive EV population decreased significantly the proteolytic activity of these EVs. In agreement with all previous observations, we also found significantly higher lytic activity associated with EV^+ TGF−β1^ compared to EV^Ctrl^, since these EVs carry higher levels of MMP2 and MMP9 (Fig. 5H). Furthermore, dose-dependent experiments more convincingly revealed the higher potency of the EV^+ TGF−β1^ to promote such proteolytic activity (Supplementary Fig. S15A).

We employed an MMP inhibitor (MMPi; GM600), targeting MMP1 (Ki: 0.4 nM), MMP2 (Ki: 0.5 nM), MMP3 (Ki: 27 nM), MMP8 (Ki: 0.1 nM) and MMP9 (Ki: 0.2 nM), used at 25 µM, a dose that was not toxic to cells (Supplementary Fig. S15B). Pre-treatment of EVs with MMPi for 30 min at 37 °C efficiently blocked the gelatin/collagen proteolytic activity promoted by MDA EVs (Fig. 5H). Finally, treatment of the EVs with MMPi or proteinase K, the latter stripping the vesicular surface from attached or embedded membrane proteins, significantly inhibited the MDA EV-induced CAGA_12_-luciferase activity in (homotypic) MDA-MB-231 and (heterotypic) BNF3 cells (Fig. 5I, Supplementary Fig. S15C). Since MMPi was equally effective as proteinase K in blocking EV-mediated TGF-β signaling, MMP9 (or another MMP) must play an important role in facilitating TGF-β signaling by tumor cell-derived EVs, most logically, by activating the predominant latent TGF-β form (Fig. 4I). Of note, similar concentration of MMPi was more efficient blocking VSF^Ctrl^ activity than VSF^+ TGF−β1^, which also corroborates the higher levels of MMPs on EVs stimulated with TGF-β1 (Fig. 5I, Supplementary Fig. S15C). As control for these experiments, bioactive, recombinant TGF-β1 was pre-treated with MMPi or proteinase K, and as expected, MMPi had minor (MDA-MB-231) or no (BNF3) effect (Fig. 5I, Supplementary Fig. S15C). Furthermore, proteinase K significantly blocked the signaling by recombinant TGF-β1 in both MDA-MB-231 and BNF3 cells, which attests to the efficiency of protein digestion under the employed conditions (Fig. 5I, Supplementary Fig. S15C). Hence, our data indicate that MMP activity carried by EVs (such as MMP9 and other MMPs) activates TGF-β ligand from its latent form carried by the EVs, thus inducing signaling in recipient cells.

Since EVs can activate TGF-β signaling in recipient cells, it was natural to assume that such intercellular crosstalk mediated by tumor-derived EVs could provide invasive potential. Thus, MDA-MB-231 and ZR-75-1 cells pre-stimulated for 48 h with TGF-β1 or with similar numbers of MDA EV^Ctrl^ and EV^+ TGF−β1^, demonstrated higher motility compared to untreated controls, without significant differences between EV^Ctrl^ and EV^+ TGF−β1^ (Supplementary Fig. S15D, E). In addition, MDA-MB-231 and BNF3 cells seeded across Matrigel, acquired a more invasive phenotype after the cells had been pre-treated with either MDA EV^Ctrl^ or EV^+ TGF−β1^ for 48 h (Fig. 5J, Supplementary Fig. S15F, G). Recipient cell pre-incubation with the MMPi for 30 min before EV delivery to them, showed that MMPi alone did not affect the motility of MDA-MB-231 and BNF3 cells, but abrogated the invasion promoted by MDA EVs (Fig. 5J, Supplementary Fig. S15G), indicating that MMPi did not act on cellular MMPs that support invasion. Depletion of the CD81-positive MDA EV population reduced the invasive response of the recipient cells significantly, demonstrating again a role for CD81-specific EV subpopulations to this biological response (Supplementary Fig. S15F).

Related to the migration and invasion experiments, the availability of breast fibroblasts allowed us to test the established actions of TGF-β/MMPs on fibrogenic responses. BNF2 and BNF3 fibroblasts treated with either MDA EV^Ctrl^ or EV^+ TGF−β1^ for 48 h, acquired an elongated morphology and showed increased FN1 protein levels intracellularly and in their extracellular matrix, an established fibrogenic response (Supplementary Fig. S16). Immunostaining for α-Smooth Muscle Actin (α-SMA), the hallmark protein of activated and contractile fibroblasts, revealed significant α-SMA expression by the fibroblasts, without further increase of α-SMA after MDA EV treatment (Supplementary Fig. S16). These fibroblast experiments confirm that the MDA EVs can transfer TGF-β and MMPs, explaining the spindle-like morphological transformation of these cells. Furthermore, invasiveness in vivo was assessed. CM-Dil Dye-labeled MDA-MB-231 cells treated for 48 h with either MDA EV^Ctrl^ or EV^+ TGF−β1^ were injected in the duct of Cuvier of transgenic Tg(*Fli1*:*EGFP*) zebrafish embryos, an established in vivo system for EV functionality [41]. After 24 h, there was a significant increase in the number of labeled cells that extravasated from the blood vessels and invaded the collagenous matrix of the tail in comparison to the non-treated cells (Fig. 5K). Hence, our data strengthen the relevance of EV-associated MMPs activating TGF-β signaling and enhancing invasive phenotype in the recipient cells.

### EVs transfer resistance to cytotoxic drugs

Resistance to anticancer drugs is facilitated by complex mechanisms, including TGF-β, EMT and tumor-derived EVs [6, 8]. To determine the effect of MDA EVs on cell viability and drug-resistance, equal numbers of MDA EVs were added to recipient MDA-MB-231 cultures (homotypic transfer) in the absence or presence of Dox or taxol co-treatment. In the absence of cytotoxic drugs, TGF-β1 stimulation suppressed the proliferation of MDA-MB-231 cells, while treatment with either MDA EV^Ctrl^ or EV^+ TGF−β1^ for 48 h significantly increased the percentage of Ki67-positive cells (Supplementary Fig. S17A). The proliferation response to EV treatment reflected the respective numbers of viable cells (Supplementary Fig. S17B), which was further validated in MCF10A, BNF3, MCF7 and ZR-75-1 cells (Supplementary Fig. S17C-F, respectively). Furthermore, EV-treated MDA-MB-231 and ZR-75-1 cells became more resistant to the cytotoxic drugs (Supplementary Fig. S17B). To confirm that the resistance to cytotoxicity was EV-mediated, MDA-MB-231 cells were treated with MDA EVs depleted of their CD81^+^ vesicular fraction. Drug response assays confirmed that CD81-EV depletion abrogated the proliferation and resistance-transfer phenotypes compared to non-depleted MDA EVs (Supplementary Fig. S17B). Notably, EVs collected from the non-tumorigenic MCF10A cells did not induce proliferation or drug resistance in MDA-MB-231 or ZR-75-1 cells (Supplementary Fig. S17G, H).

To validate the latter data in vivo, we treated MDA-MB-231 cells with or without EVs and injected the cells in zebrafish embryos that lived in water containing vehicle or 5 µM Dox (Fig. 5L). The number of extravasated cell clusters 48 h after injection in the presence of Dox was significantly lower relative to untreated cells (compare the quantification -scale difference- in Fig. 5K and L). Under the latter condition, a significant increase in the number of extravasated cell clusters from MDA-MB-231 cells treated with EV^Ctrl^ or EV^+ TGF−β1^ compared to the non-EV treated cells was observed (Fig. 5L), indicating that MDA EVs increased the proportion of drug-resistant MDA-MB-231 cells circulating in the vasculature of the fish.

In order to connect the transfer of chemoresistance to TGF-β signaling, the recipient cells were pre-incubated with the same as previously used TβRi. In contrast to recombinant TGF-β1 that reduced recipient cell viability, TβRi preserved the cell viability (Supplementary Fig. S17I). In the presence of taxol or Dox, TβRi significantly reduced the enhanced resistance (measured as viability) caused by either recombinant TGF-β1, VSF^Ctrl^ or VSF^+ TGF−β1^ (Supplementary Fig. S17J, K). Hence, MDA EVs enhance cell viability in tumor and non-tumorigenic cells and consequently increase the chemoresistance of cancer cells, at least in part due to the activation of TGF-β signaling. Taken together, our data show that an equal number of EV^Ctrl^ or EV^+ TGF−β1^ induced similar responses to cell motility and drug resistance in recipient cells, emphasizing that the primary effect of TGF-β signaling is the enhanced production of EVs.

## Discussion

We investigated EV biology by studying normal human breast epithelial cells and fibroblasts in addition to breast and lung cancer cells. The signaling mechanism initiated by TGF-β receptors, is followed by MEK/ERK1/2 kinase activation and SREBP2 phosphorylation, which regulates DHCR7 expression and cholesterol biosynthesis, thus providing inputs to one of the major routes of EV biogenesis (Fig. 3I). Furthermore, the analysis of the protein-based cargo of EVs led to the demonstration that TGF-β (primarily in its latent form) and MMP9 carried by EVs, the latter specially enriched in EVs generated after TGF-β stimulation, have biological activity. TGF-β carried as EV cargo signals on recipient cells through plasma membrane receptors and does not require EV uptake to elicit function. MMP9 (or other MMPs) in EVs seem to be involved in activation of latent TGF-β carried by the EVs and also in EV-mediated cell invasiveness (Fig. 5M).

Our finding that MEK/ERK1/2 primarily mediates TGF-β-induced EV secretion, agrees with a report, not related to TGF-β, that implicated MEK/ERK1/2 signaling in EV production by MVBs in colorectal and renal cancer cells [13]. One of the factors that control EV biogenesis is lipid/cholesterol synthesis, since the membranes of EVs are enriched in cholesterol, and microvesicles are specifically secreted from lipid rafts [28]. Accordingly, simvastatin, which inhibits HMGCR, limits the cholesterol pool, and inhibits EV secretion [42], as we also demonstrated in this study. Conversely, cholesterol can also modulate the rate of secretion and the cargo of EVs in BRCA cells [43]. We demonstrate that DHCR7 expression [36] is induced by TGF-β via activation of MEK/ERK1/2 leading to phosphorylation of SREBP2. Our observations are in line with other findings of SREBP2 activation by ERK1/2 [32], and of induction of *DHCR7* expression by SREBP2 [31]. Interestingly, the late response of SREBP2 phosphorylation and DHCR7 expression to TGF-β stimulation, beyond being novel, it is compatible with the slow signaling process observed regarding the secretion of EVs induced by TGF-β and the roles of cholesterol in EV biogenesis. Of note, TGF-β not only regulates *DHCR7* and *SQLE* expression, as in our models, but can also induce *HMGCR* expression in keratinocytes, leading to increased cholesterol synthesis [30]. Thus, the current study expands a critical aspect of EV biology related to the contribution of cholesterol synthesis. Additionally, we demonstrate that combinations of FDA-approved inhibitors of cholesterol biosynthesis with established cytotoxic drugs [44], sensitize BRCA and LUAD cells to undergo cell death. The interesting possibility that the effect of statins involves suppression of EV biogenesis, deserves to be examined.

The unbiased proteomic analysis of CD81- and GM1-enriched EVs showed that TGF-β stimulation modulated the cargo of small EVs (< 200 nm), for instance enriching EVs with MMP9 among a few hundred other proteins, but without altering dramatically their morphology or functionality. Furthermore, TGF-β led to the regulation of several EV-associated proteins. A study where the MDA-MB-231 EV proteome was compared to the EV proteome of non-malignant MCF10A cells (outside the context of growth factor signaling like TGF-β), concluded that specific protein cargo reflected the secretome of each cell type [45]. A similar conclusion was reached from BRCA EV proteomic studies that confirmed that the EV proteome can indicate the cell of origin and thus discriminate between epithelial and mesenchymal BRCA cells [46]. Proteomic analysis of EVs secreted from colorectal cancer cells that carry mutant, kinase deficient TGF-β type II receptors, revealed an EV cargo enriched in extracellular matrix and nucleosomal proteins [47], but it is not known whether such enrichment was due to altered TGF-β signaling or whether it reflected cell adaptation to the mutation. Our model proposes that TGF-β primarily influences the rate of EV secretion by elevating cholesterol levels, and regulates the sorting of a select number (a few hundred) of EV cargo content, including modulation of the levels of pre-existing EV cargo proteins. We studied deeper two cargo proteins present in both EV^Ctrl^ and EV^+ TGF−β1^ since they are functionally interlinked. TGF-β, whose levels were not further modulated in EV^+ TGF−β1^ relative to EV^Ctrl^, and MMP9 whose levels were selectively increased in EV^+ TGF−β1^. Signaling and invasiveness studies also served as functional correlates to the presence of latent TGF-β and MMP9 on EVs. Such signaling analyses should be interpreted with caution as several of the signaling proteins studied (e.g. pERK1/2, pAKT) are not unique to TGF-β signaling and can provide signaling readouts of several alternative EV cargo proteins. Since SMAD C-terminal phosphorylation is a unique signaling readout of TGF-β family ligands throughout animal evolution, we safely conclude that EVs activate downstream signaling that represents partially cargo TGF-β (or another member of its family) and partially cooperating cargo molecules.

TGF-β in association with its proteoglycan co-receptor β-glycan/TGFBR3, both carried by EVs, induces the transition of fibroblasts to myofibroblasts [5]. Similarly, mast cell-secreted EVs carry both latent and mature TGF-β on their surface, and upon interaction with recipient mesenchymal stem cells, the TGF-β associated with EVs can signal from endosomes after EV internalization [48]. We present a complementary mechanism, since heparin, an established inhibitor of EV uptake [38] did not block signaling in the cancer cell studied. Thus, TGF-β signaling mediated by EVs can also be exerted by ligand tethered to the EV surface. EVs also carry the α_v_β_6_ integrin that associates with and activates latent TGF-β [49], or the tetraspanins CD9 and CD81, which can form complexes with the signaling TGF-β receptors [50], possibly providing a supra-molecular machinery for TGF-β activation. Overexpression of MMP9 has been reported to proteolytically cleave latent TGF-β and activate this signaling pathway in BRCA cells, propagating malignant cell invasiveness [40, 51]. We propose that TGF-β signaling can lead to specific enrichment of MMP9 on EVs, which can activate latent TGF-β in the EV cargo, a mechanism compatible with the well-established auto-induction principle of TGF-β [7]. It will also be interesting to analyze the co-segregation of different TGF-β family ligands with MMP family members in EV populations generated by BRCA or LUAD tumors. However, it is worth presenting a critical view on the above model and more general on the validity of EV cargo proteins [2]. In the absence of detailed topological studies such as cryo-electron tomography, the EV cargo, and especially secreted proteins that are identified tethered to the EV membrane (e.g., latent TGF-β1, MMPs, etc.) may represent genuine proteins properly packaged during EV biogenesis; alternatively, such proteins can be coating the EV surface due to a protein corona effect or they may even represent fortuitously associated proteins due to the method of EV preparation. The previous rigorous demonstration of latent TGF-β1 on the surface of exosomes [48], generates optimism that the molecular mechanism described here may well take place on the EV surface under physiological conditions. Lastly, our evidence on transfer of chemoresistance by EVs, is consistent with previous reports whereby resistance to doxorubicin or paclitaxel was transferred via TNBC EVs to non-malignant MCF10A cells [52]. Furthermore, TGF-β as EV cargo has been linked to transfer of resistance to anti-cancer drugs in head-and-neck squamous cell carcinoma and BRCA [15, 53], a phenotype that here was partially inhibitable by a TGF-β type I receptor inhibitor.

## Conclusions

In summary, this study provides a fresh view on signaling mechanisms that regulate the secretion of EVs and highlights the role of cholesterol biosynthesis, TGF-β signaling and matrix metalloproteases as mechanisms of regulation of latent TGF-β cargo carried by EVs, leading to diverse pro-tumorigenic biological effects on recipient cancer cells.

## Electronic supplementary material

Below is the link to the electronic supplementary material.

Supplementary Material 1

Supplementary Material 2

## Data Availability

The proteomics data are deposited in to PRIDE (https://www.ebi.ac.uk/pride/) under Accession Number PXD039591. For reviewer use, the details are: https://www.ebi.ac.uk/pride/; Username: reviewer_pxd039591@ebi.ac.uk Password: gBpMY8J0. The additional experimental resources used and/or analyzed during the current study are available from the corresponding author on reasonable request.

## Abbreviations

BP: GO-biological process
BRCA: Breast cancer
CC: GO-cellular component
CTB: Cholerae toxin chain-B
Dox: Doxorubicin
EMT: Epithelial-mesenchymal transition
ER: Estrogen receptor-α
ESCRT: Endosomal sorting complexes required for transport
EV: Extracellular vesicle
FDR: False discovery rate
GO: Gene ontology
GSEA: Gene set enrichment analysis
HER2: Human epidermal growth factor receptor 2
HMGCR: HMG-CoA reductase
LC-ESI-MS/MS: Liquid Chromatography Electrospray Ionization Tandem Mass Spectrometry
L-TGF-β1: Latent TGF-β1
LUAD: Lung adenocarcinoma
MF: GO-molecular function
MMP: Matrix metalloprotease
MVB: Multi-vesicular body
NTA: Nanoparticle tracking analysis
PCA: Principal component analysis
PgR: Progesterone receptor
REAC: Reactome
SEM: Scanning electron microscopy
α-SMA: α-Smooth muscle actin
SREBP2: Sterol regulatory element-binding protein-2
TEM: Transmission electron microscopy
TGF-β: Transforming growth factorβ
TGFβRI: TGF-βreceptor type I
TGFβRII: TGF-βreceptor type II
TGFBI: TGF-β-induced
TNBC: Triple-negative breast cancer
TRF: Transferrin
VSF: Vesicular secretome fraction
Xi: Inhibitor of molecule X
