# Supplementary material for: TGF-β induces cholesterol accumulation to regulate the secretion of tumor-derived extracellular vesicles
Source: J Exp Clin Cancer Res. 2025 Feb 6;44:42. doi: 10.1186/s13046-025-03291-0 (PMC11800471; doi:10.1186/s13046-025-03291-0)
Supplement: Supplementary file 1 — Supplementary Material 1 [file 13046_2025_3291_MOESM1_ESM.pdf]

SUPPLEMENTARY FILES

TGF-β induces cholesterol accumulation to regulate the secretion of tumor-derived extracellular vesicles

Dorival Mendes Rodrigues-Junior[1], Chrysoula Tsirigoti[1,2], Konstantina Psatha[3], Dimitris Kletsas[4], Michalis Aivaliotis[3], Carl-Henrik Heldin[1], Aristidis Moustakas[1]*

[1]*Department of Medical Biochemistry and Microbiology, Science for Life Laboratory, Box 582, Biomedical Center, Uppsala University, SE-751 23 Uppsala, Sweden.*

[2]*Astra Zeneca, Pepparedsleden 1, SE-431 83 Mölndal, Sweden.*

[3]*Laboratory of Biochemistry, School of Medicine, Faculty of Health Sciences, Aristotle University of Thessaloniki, GR-541 24 Thessaloniki, Greece.*

[4]*Laboratory of Cell Proliferation & Ageing, Institute of Biosciences and Applications, National Centre for Scientific Research 'Demokritos', GR-153 10 Athens, Greece.*

TABLE OF CONTENT:

SUPPLEMENTARY FIGURES AND LEGENDS

SUPPLEMENTARY TABLES AND DATA SOURCE COMBINED

SUPPLEMENTARY FIGURES AND LEGENDS

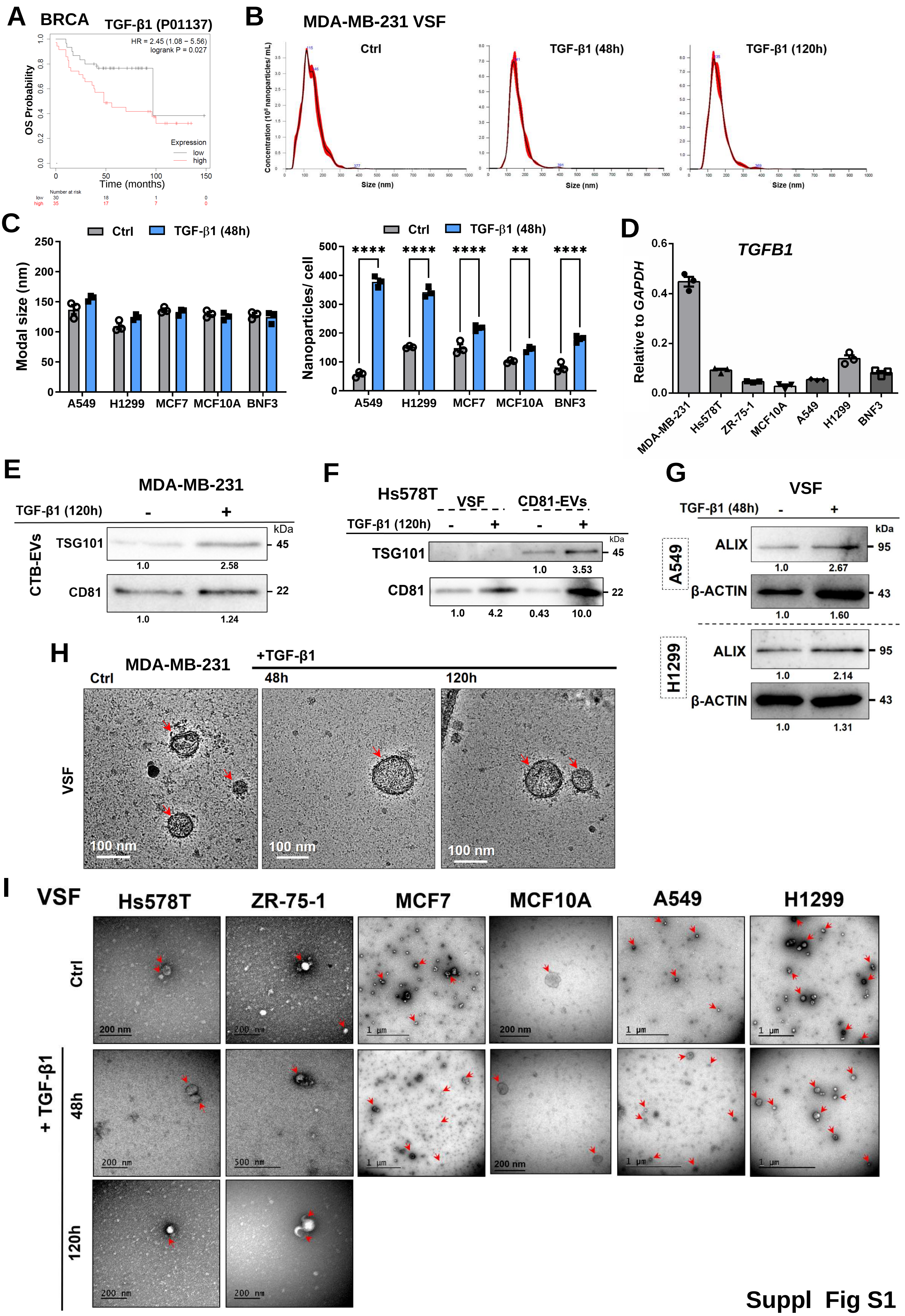

A
BRCA
TGF-β1 (P01137)
HR = 2.45 (1.08 − 5.56)
logrank P = 0.027
OS Probability
Time (months)
Expression
low
high
B
MDA-MB-231 VSF
Ctrl
TGF-β1 (48h)
TGF-β1 (120h)
Size (nm)
C
Ctrl
TGF-β1 (48h)
Modal size (nm)
Nanoparticles/ cell
A549
H1299
MCF7
MCF10A
BNF3
D
TGFB1
Relative to GAPDH
MDA-MB-231
Hs578T
ZR-75-1
MCF10A
A549
H1299
BNF3
E
MDA-MB-231
TGF-β1 (120h)
CTB-EVs
TSG101
CD81
kDa
F
Hs578T
VSF
CD81-EVs
TGF-β1 (120h)
TSG101
CD81
G
VSF
TGF-β1 (48h)
A549
H1299
ALIX
β-ACTIN
H
MDA-MB-231
+TGF-β1
Ctrl
48h
120h
VSF
100 nm
I
VSF
Hs578T
ZR-75-1
MCF7
MCF10A
A549
H1299
Ctrl
+ TGF-β1
48h
120h

Suppl Fig S1

Supplementary Fig S1. Related to Fig 1. Characterization of EVs secreted from human cancer and normal cells used in the study. (A) Higher TGF-β1 protein levels correlate with shorter overall survival (OS) in human basal BRCA patients. BRCA samples were stratified and compared in the Kaplan-Meier plotter database using auto select cut-off. **(B)** Representative nanoparticle traces. The VSF from MDA-MB-231 cells stimulated with 5 ng/mL TGF-β1 for 48 or 120 h or not (Ctrl) were analyzed by Nanoparticles Tracking Analysis (NTA) according to the manufacturer's protocol. **(C)** EVs released by the five indicated cell models quantified by NTA in terms of particle size (left) and particle number after normalization to the total cell number (right). The cells were stimulated with vehicle (Ctrl) or 5 ng/mL TGF-β1 for 48 h. **(D)** RT-qPCR was used for detection of *TGFB1* mRNA levels in the seven indicated cell models. All the relative expression levels were normalized to *GAPDH* expression and calculated using the $2^{-\Delta Ct}$ method. **(E-G)** Detection of the indicated EV-specific proteins and β-ACTIN in EV extracts (isolated after CTB-specific enrichment (E), as VSF or after CD81-specific enrichment (F), or as VSF (F, G) derived from Hs578T (F), A549 or H1299 (G) cells stimulated with 5 ng/mL TGF-β1 for 120 h (E, F) or 48 h (G); densitometric values were normalized to the vehicle control and molecular size markers are shown. **(H)** Representative cryo-EM pictures of EVs isolated as VSF from MDA-MB-231 cells stimulated with 5 ng/mL TGF-β1 for 48 or 120 h or not (Ctrl). **(I)** Representative TEM pictures of EVs isolated as VSF from the indicated cells stimulated with 5 ng/mL TGF-β1 for 48 or 120 h or not (Ctrl). Scale bars in H and I are included and red arrows mark specific EVs. Data in C and D are presented as mean values of three biological replicates ± SEM, each in technical duplicates and p-values in C are shown based on two-way ANOVA, followed by multiple paired comparisons conducted by means of Bonferroni's post-test method: *p ≤ 0.05; ****p ≤ 0.0001; n.s., not significant. The data in E, F and G show representative immunoblots of three independent biological replicates along with molecular mass markers in kDa.

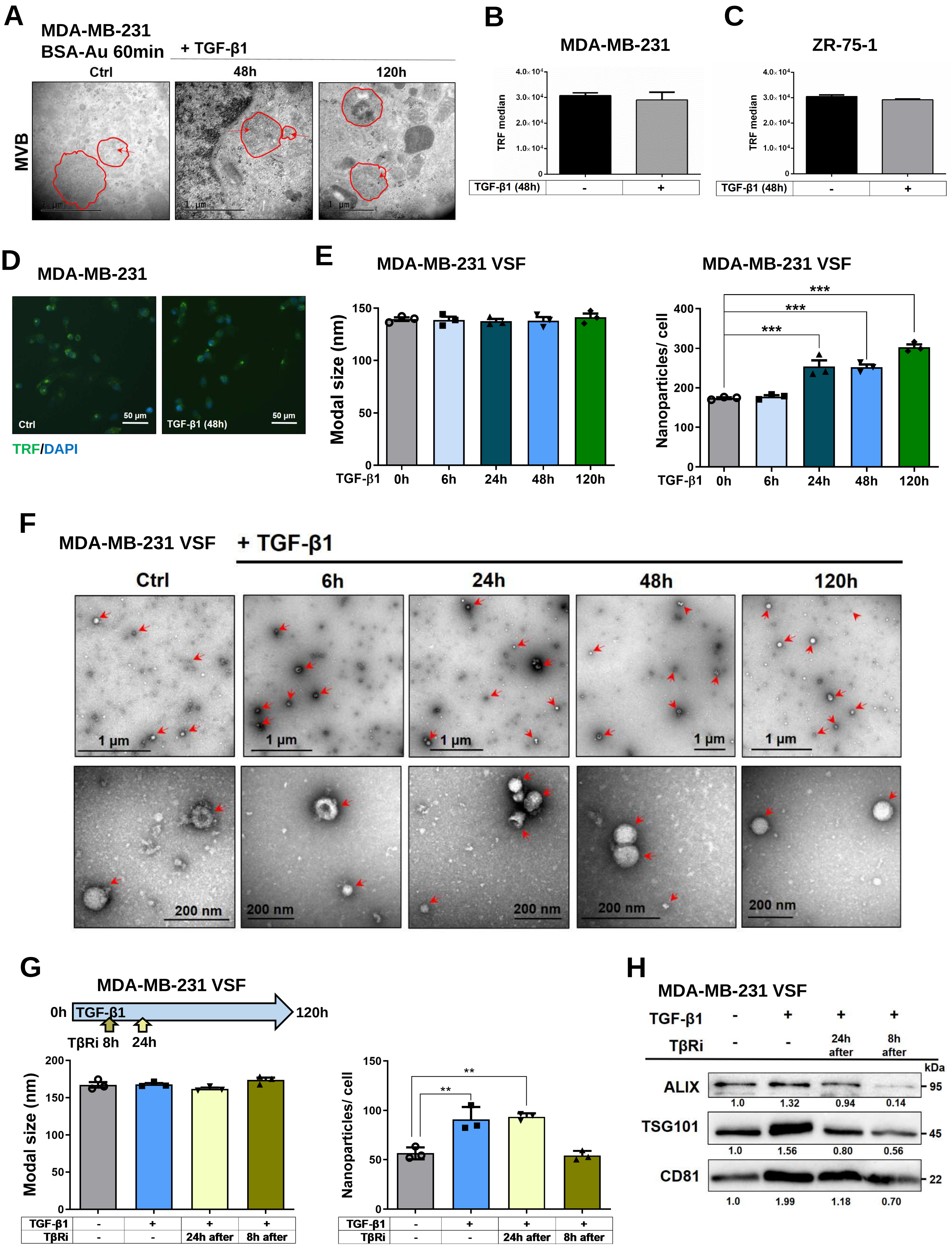
A
MDA-MB-231
BSA-Au 60min
+ TGF-β1
Ctrl
48h
120h
MVB
1 µm
B
MDA-MB-231
TRF median
TGF-β1 (48h)
C
ZR-75-1
D
MDA-MB-231
Ctrl
TGF-β1 (48h)
50 µm
TRF/DAPI
E
MDA-MB-231 VSF
Modal size (nm)
Nanoparticles/ cell
TGF-β1 0h 6h 24h 48h 120h
***
F
MDA-MB-231 VSF
+ TGF-β1
Ctrl
6h
24h
48h
120h
1 µm
200 nm
G
MDA-MB-231 VSF
0h
TGF-β1
120h
TβRi 8h
24h
Modal size (nm)
Nanoparticles/ cell
TGF-β1
TβRi
24h after
8h after
**
H
MDA-MB-231 VSF
TGF-β1
TβRi
24h after
8h after
kDa
ALIX
95
1.0 1.32 0.94 0.14
TSG101
45
1.0 1.56 0.80 0.56
CD81
22
1.0 1.99 1.18 0.70

Suppl Fig S2

Supplementary Fig S2. Related to Fig 1. TGF-β induces EV secretion as a late response and via a delayed receptor signaling activity without influencing EV ultrastructure. (A) Representative TEM pictures of MVBs in MDA-MB-231 cells stimulated with 5 ng/mL TGF-β1 for 48 or 120 h or not (Ctrl), prior to incubation with BSA-Au for 60 min. Scale bars are included and red lines indicate MVBs with red arrows marking BSA-Au internalized particles. **(B, C)** Quantification of fluorescent TRF internalized after 1 h by MDA-MB-231 (B) and ZR-75-1 (C) cells that were pre-stimulated with 5 ng/mL TGF-β1 for 48 h or not, using FACS analysis. Median values of fluorescent TRF of three biological replicates ± SEM, each in technical duplicates are plotted. **(D)** TRF uptake was visualized by direct fluorescence microscopy in MDA-MB-231 cells. Representative images are shown with scale bars. **(E)** EVs released by MDA-MB-231 cells were isolated as VSF and quantified by NTA in terms of particle size (left) and particle number after normalization to the total cell number (right). The cells were stimulated with vehicle (0 h) or 5 ng/mL TGF-β1 for the indicated time points. **(F)** Representative TEM pictures of EVs in VSF of MDA-MB-231 cells after stimulation with vehicle (0 h) or 5 ng/mL TGF-β1 for the indicated time points. Scale bars are included and red arrows indicate EVs. **(G)** EVs released by MDA-MB-231 cells were isolated as VSF and quantified by NTA in terms of particle size (left) and particle number after normalization to the total cell number (right). The cells were stimulated with vehicle (-) or 5 ng/mL TGF-β1 for 48 h. Vehicle DMSO (-) or the TβRi was added to the cells 8 or 24 h after TGF-β1 stimulation in order to monitor the time length required for signaling that leads to EV release. **(H)** Expression levels of the indicated EV-specific proteins secreted from MDA-MB-231 cells treated as in panel G. Data in E and G are presented as mean values of three biological replicates ± SEM, each in technical duplicates and p-values are shown based on one-way ANOVA, followed by multiple paired comparisons conducted by means of Bonferroni's post-test method: $**p \leq 0.01$; $***p \leq 0.001$. The data in H show representative immunoblots of three independent biological replicates along with densitometric values normalized to the vehicle control and molecular mass markers in kDa.

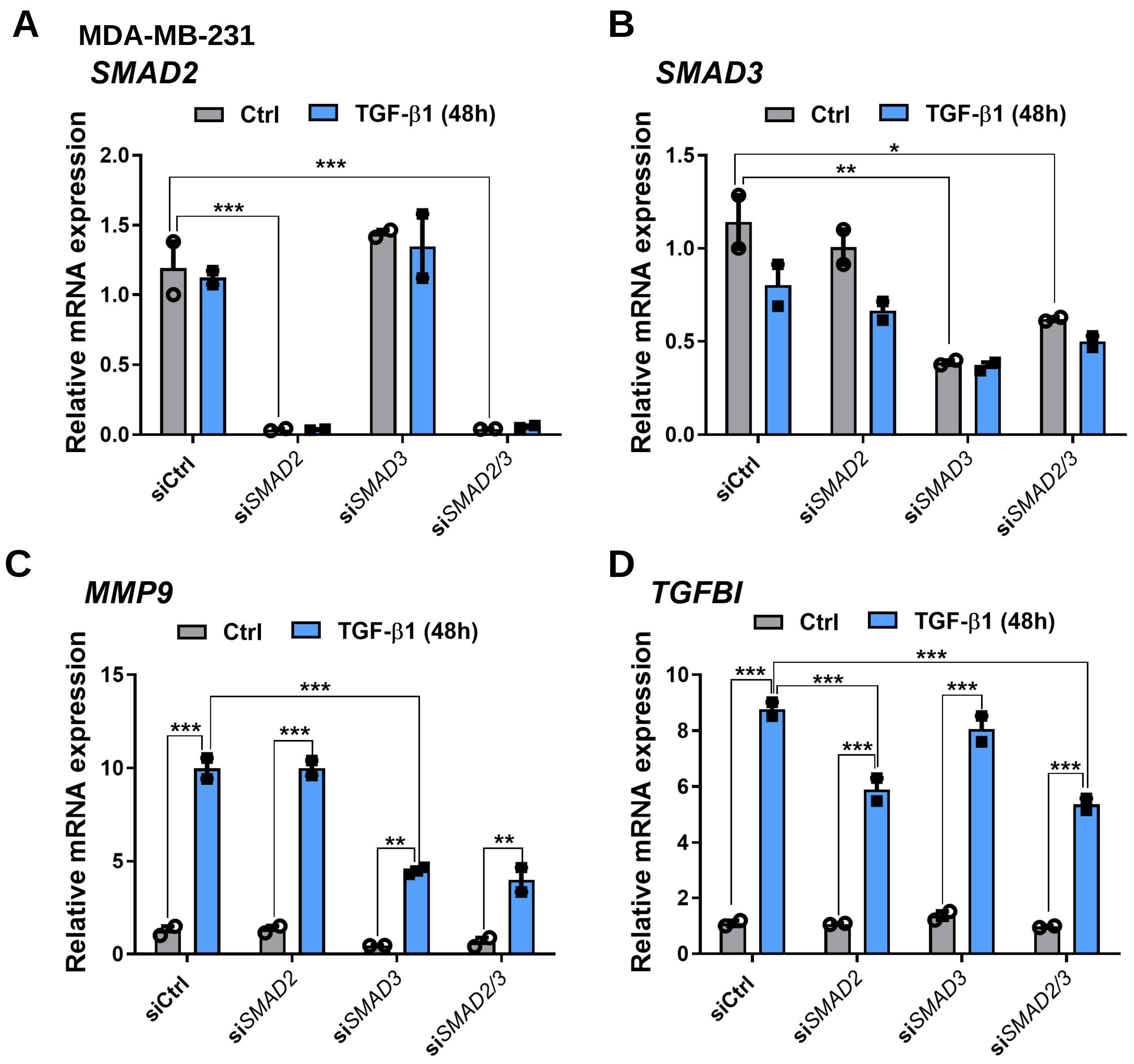
A
MDA-MB-231
SMAD2
Ctrl
TGF-β1 (48h)
Relative mRNA expression
siCtrl
siSMAD2
siSMAD3
siSMAD2/3
B
SMAD3
C
MMP9
D
TGFBI

Suppl Fig S3

Supplementary Fig S3. Related to Fig 1. SMAD2 and SMAD3 silencing efficiency and impact on *MMP9* and *TGFBI* expression*.* (A-D) RT-qPCR analysis of *SMAD2*, *SMAD3* and two TGF-β regulated genes (*MMP9* and *TGFBI*) mRNA levels in MDA-MB-231 cells transiently transfected with the indicated siRNAs and stimulated with 5 ng/mL TGF-β1 for 48 h or not (Ctrl). The data are presented as mean values of two biological replicates ± SEM, in technical triplicates and p-values are shown based on two-way ANOVA, followed by multiple paired comparisons conducted by means of Bonferroni's post-test method: p-values: *$p \leq 0.05$; **$p \leq 0.01$; ***$p \leq 0.001$.

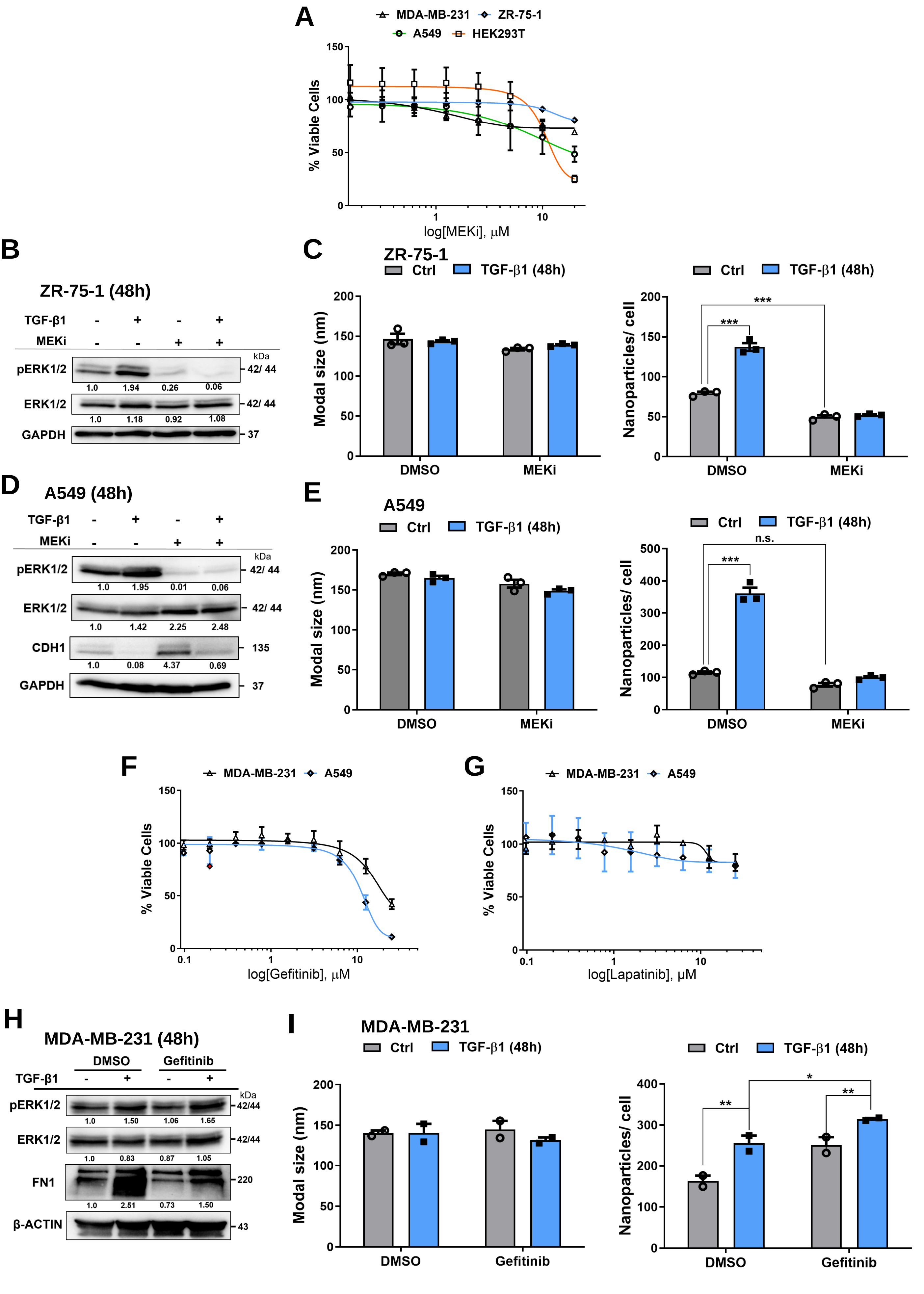

A
MDA-MB-231
ZR-75-1
A549
HEK293T
% Viable Cells
log[MEKi], µM
B
ZR-75-1 (48h)
TGF-β1
MEKi
pERK1/2
ERK1/2
GAPDH
kDa
42/ 44
37
1.0 1.94 0.26 0.06
1.0 1.18 0.92 1.08
C
ZR-75-1
Ctrl
TGF-β1 (48h)
Modal size (nm)
Nanoparticles/ cell
DMSO
MEKi
D
A549 (48h)
CDH1
135
1.0 1.95 0.01 0.06
1.0 1.42 2.25 2.48
1.0 0.08 4.37 0.69
E
A549
n.s.
F
log[Gefitinib], µM
G
log[Lapatinib], µM
H
MDA-MB-231 (48h)
DMSO
Gefitinib
FN1
β-ACTIN
42/44
220
43
1.0 1.50 1.06 1.65
1.0 0.83 0.87 1.05
1.0 2.51 0.73 1.50
I
MDA-MB-231

Suppl Fig S4

Supplementary Fig S4. Related to Fig 1. TGF-β induces EV secretion via MEK/ERK signaling in several cell models without implication of the EGFR. **(A)** Viability curves of the four indicated cell models in the presence of increasing concentrations of the MEKi (logarithmic scale). Note the lack of significant differences. **(B)** Expression levels of the indicated signaling proteins and GAPDH as loading control in ZR-75-1 cells stimulated or not with 5 ng/mL TGF-β1 for 48 h in the presence of DMSO vehicle (-) or 5 μM MEKi, and densitometric values normalized to the vehicle control. **(C)** EVs released by ZR-75-1 cells treated as in panel B and quantified by NTA in terms of particle size (left) and particle number after normalization to the total cell number (right). **(D)** Expression levels of the indicated proteins and GAPDH (as loading control) in A549 cells stimulated with 5 ng/mL TGF-β1 in the absence or presence of 5 μM MEKi for 48 h, and densitometric values normalized to the vehicle control. **(E)** EVs released by A549 cells treated with vehicle (DMSO) or 5 μM MEKi and quantified by NTA in terms of particle size (left) and particle number after normalization to the total cell number (right). **(F, G)** Viability curves of the two indicated cell models in the presence of increasing concentrations of the EGFR inhibitors Gefitinib (F) and Lapatinib (G) (logarithmic scale). **(H)** Expression levels of the indicated proteins and β-ACTIN (as loading control) in MDA-MB-231 cells stimulated with 5 ng/mL TGF-β1 in the absence or presence of 5 μM Gefitinib for 48 h, and densitometric values normalized to the vehicle control. **(I)** EVs released by MDA-MB-231 cells treated with vehicle (DMSO) or 5 μM Gefitinib and quantified by NTA in terms of particle size (left) and particle number after normalization to the total cell number (right). The data in B, D and H show representative immunoblots of three independent biological replicates along with molecular mass markers in kDa. Data in A, F and G are shown as mean values of three biological replicates ± SEM, each in technical triplicates. Data in C, E and I are indicated as mean values of three biological replicates ± SEM, each in technical duplicates and p-values are shown based on two-way ANOVA, followed by multiple paired comparisons conducted by means of Bonferroni's post-test method, p-values: $^{**}p \leq 0.01$; $^{***}p \leq 0.001$; n.s., not significant.

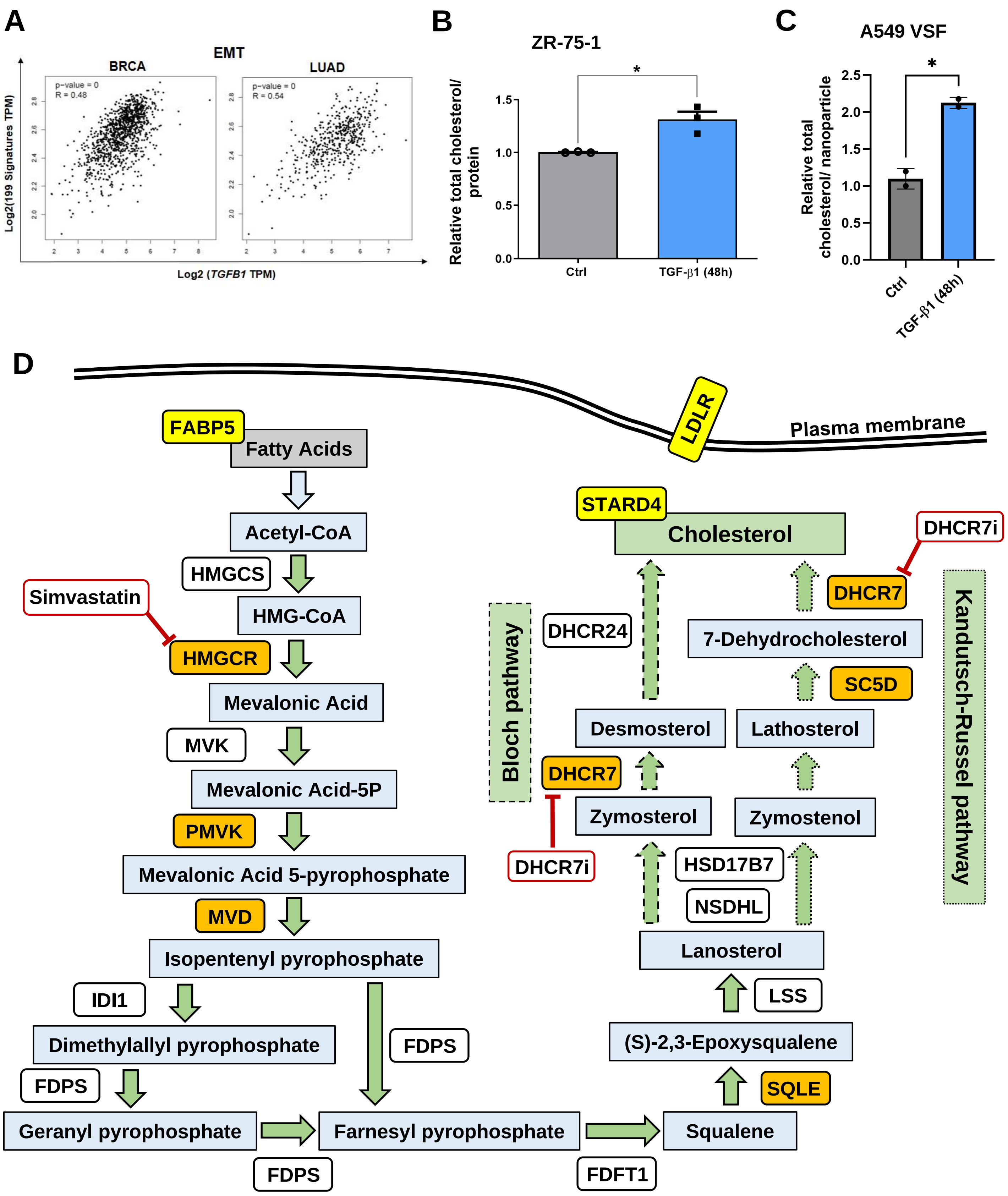
A
EMT
BRCA
LUAD
p-value = 0
R = 0.48
p-value = 0
R = 0.54
Log2(199 Signatures TPM)
Log2 (TGFB1 TPM)
B
ZR-75-1
Relative total cholesterol/ protein
*
1.5
1.0
0.5
0.0
Ctrl
TGF-β1 (48h)
C
A549 VSF
Relative total cholesterol/ nanoparticle
*
2.5
2.0
1.5
1.0
0.5
0.0
Ctrl
TGF-β1 (48h)
D
LDLR
Plasma membrane
FABP5
Fatty Acids
Acetyl-CoA
HMGCS
HMG-CoA
Simvastatin
HMGCR
Mevalonic Acid
MVK
Mevalonic Acid-5P
PMVK
Mevalonic Acid 5-pyrophosphate
MVD
Isopentenyl pyrophosphate
IDI1
Dimethylallyl pyrophosphate
FDPS
FDPS
Geranyl pyrophosphate
FDPS
Farnesyl pyrophosphate
FDFT1
Squalene
SQLE
(S)-2,3-Epoxysqualene
LSS
Lanosterol
HSD17B7
NSDHL
Zymosterol
Zymostenol
DHCR7
DHCR7i
Desmosterol
Lathosterol
SC5D
7-Dehydrocholesterol
DHCR24
DHCR7
DHCR7i
STARD4
Cholesterol
Bloch pathway
Kandutsch-Russel pathway

Suppl Fig S5

Supplementary Fig S5. Related to Fig 2. TGF-β induces cholesterol synthesis in cancer cells and diagram of the biosynthetic pathway. (A) Pearson correlation analysis of mRNA expression in BRCA and LUAD of gene signatures for EMT relative to the *TGFB1* mRNA expression, measured as transcripts per million (TPM) transformed by log_2; data was obtained from TCGA. P- and R-values are listed. **(B)** Quantification of total cholesterol levels in ZR-75-1 cells, stimulated with 5 ng/mL TGF-β1 for 48 h. **(C)** Quantification of total cholesterol levels in VSF enriched from A549 cells stimulated with 5 ng/mL TGF-β1 for 48 h. The data in B and C are presented as mean values of at least two biological replicates ± SEM, in technical triplicates and p-values are shown based on unpaired student's *t*-test: *p ≤ 0.05. **(D)** Simplified scheme of the cholesterol synthesis pathway. Upon β-oxidation of fatty acids (grey), acetyl-coenzyme A (Acetyl-CoA) in blue is converted through more than 20 enzymatic reactions to synthesize cholesterol (green). The pathway is divided into two branches after lanosterol synthesis. These two branches are known as the Bloch and the Kandutsch-Russel pathways and are indicated with differentially dotted arrows for distinction. Note that not all enzymes of the biosynthetic pathway are shown for reasons of brevity. In orange are the enzymes analyzed here (HMGCR, PMVK, MVD, SQLE, SC5D and DHCR7), while the fatty acids and cholesterol transporters FABP5 and STARTD4 and the low-density lipoprotein receptor (LDLR) are presented in yellow. The cholesterol reducing drugs (Simvastatin and DHCR7i) are presented inside boxes with red outlines.

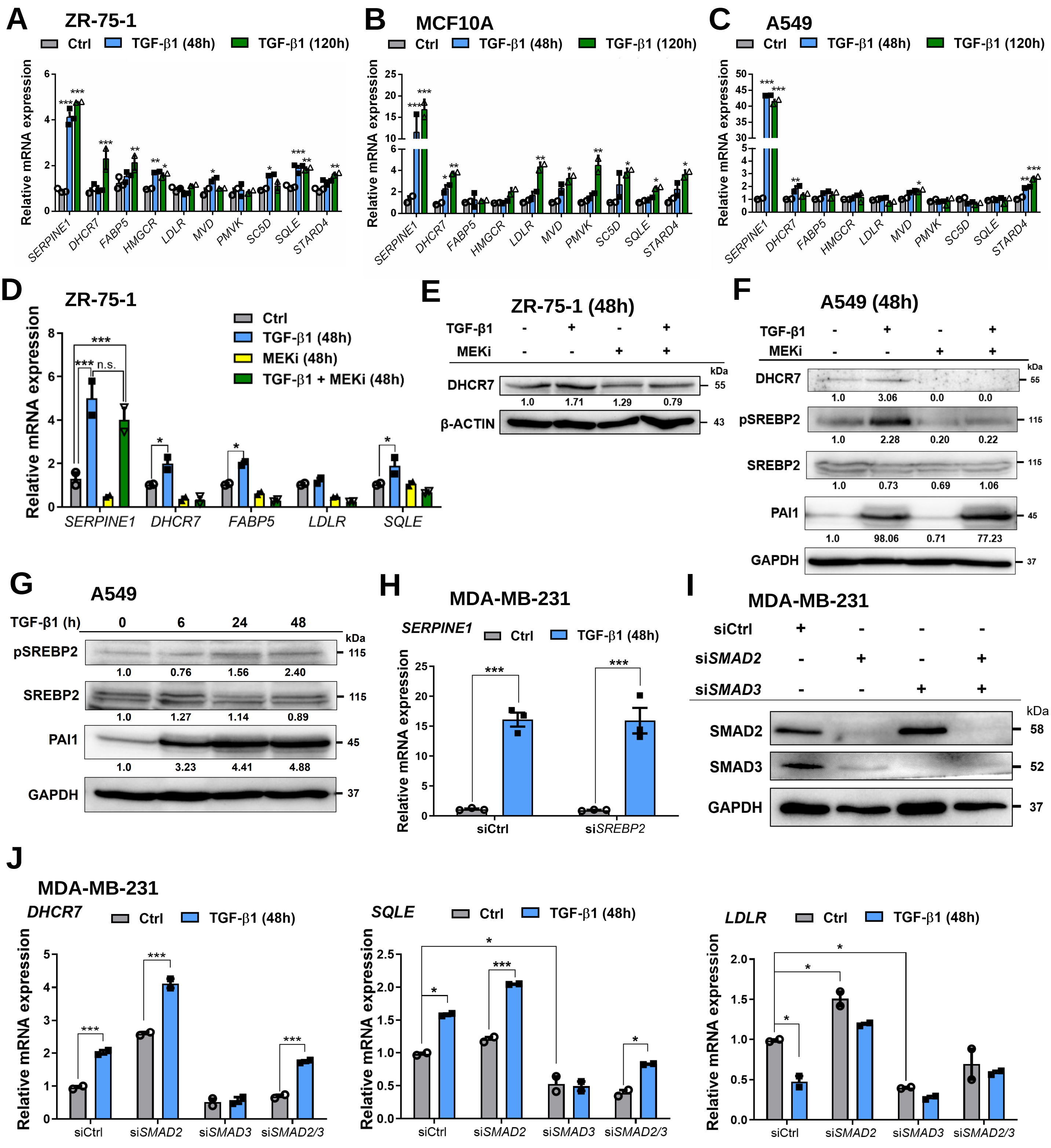
A
ZR-75-1
Ctrl
TGF-β1 (48h)
TGF-β1 (120h)
Relative mRNA expression
SERPINE1
DHCR7
FABP5
HMGCR
LDLR
MVD
PMVK
SC5D
SQLE
STARD4
B
MCF10A
C
A549
D
ZR-75-1
MEKi (48h)
TGF-β1 + MEKi (48h)
n.s.
E
ZR-75-1 (48h)
TGF-β1
MEKi
DHCR7
β-ACTIN
kDa
55
43
1.0 1.71 1.29 0.79
F
A549 (48h)
pSREBP2
SREBP2
PAI1
GAPDH
1.0 3.06 0.0 0.0
1.0 2.28 0.20 0.22
1.0 0.73 0.69 1.06
1.0 98.06 0.71 77.23
115
45
37
G
A549
TGF-β1 (h)
0 6 24 48
1.0 0.76 1.56 2.40
1.0 1.27 1.14 0.89
1.0 3.23 4.41 4.88
H
MDA-MB-231
SERPINE1
siCtrl
siSREBP2
I
MDA-MB-231
siCtrl
siSMAD2
siSMAD3
SMAD2
SMAD3
58
52
J
MDA-MB-231
siSMAD2/3

Suppl Fig S6

Supplementary Fig S6. Related to Fig 2. TGF-β modulates gene expression of cholesterol pathway genes. (A-C) RT-qPCR analysis of the indicated mRNA levels in ZR-75-1 (A), MCF10A (B) and A549 (C) cells upon stimulation with 5 ng/mL TGF-β1 for 48 or 120 h. Values represent the mean values of at least two biological replicates ± SEM, as the fold-change of mRNA expression normalized to *GAPDH* and relative to the level at 0 h TGF-β1 (Ctrl). **(D)** RT-qPCR analysis of the indicated mRNA levels in ZR-75-1 cells stimulated with 5 ng/mL TGF-β1 in the absence or presence of 5 µM MEKi for 48 h. Values represent fold-change of mRNA expression normalized to *GAPDH* and expressed relative to the level at 0 h TGF-β1 (Ctrl). **(E)** Expression levels of DHCR7 and GAPDH serving as loading control from ZR-75-1 cells upon stimulation with 5 ng/mL TGF-β1 in the absence or presence of 5 µM MEKi for 48 h, and densitometric values were normalized to the vehicle control. **(F)** Expression levels of DHCR7, pSREBP2, SREBP2, PAI1 and GAPDH serving as loading control from A549 cells treated as in panel D, and densitometric values were normalized to the vehicle control. **(G)** Expression levels of pSREBP2, SREBP2, PAI1 and GAPDH serving as loading control from A549 cells treated with TGF-β1 for 6, 24 and 48 h. Densitometric values were normalized to the vehicle control. **(H)** RT-qPCR analysis of the *SERPINE1* mRNA in MDA-MB-231 cells after transient transfection with siCtrl or si*SREBP2* after stimulation of the transfected cells with 5 ng/mL TGF-β1 for 48 h. Values represent fold-change of mRNA expression normalized to *GAPDH* and expressed relative to the level at siCtrl. **(I)** Expression levels of SMAD2 and SMAD3 proteins with GAPDH serving as loading control in MDA-MB-231 cells transiently transfected with siCtrl or specific siRNAs targeting SMAD2 or SMAD3. **(J)** RT-qPCR analysis of the three indicated mRNAs in MDA-MB-231 cells after transient transfection with siCtrl, *SMAD2*-specific, *SMAD3*-specific siRNAs or their combination after stimulation of the transfected cells with 5 ng/mL TGF-β1 for 48 h. Values represent fold-change of mRNA expression normalized to *GAPDH* and expressed relative to the level at siCtrl. The data in E-G and I show representative immunoblots of at least two independent biological replicates along with molecular mass markers in kDa. Data in A-D, H and J are presented as mean values of at least two biological replicates ± SEM, each in technical duplicates and p-values are shown based on two-way ANOVA, followed by multiple paired comparisons conducted by means of Tukey's post-test method: *p ≤ 0.05; **p ≤ 0.01; ***p ≤ 0.001.

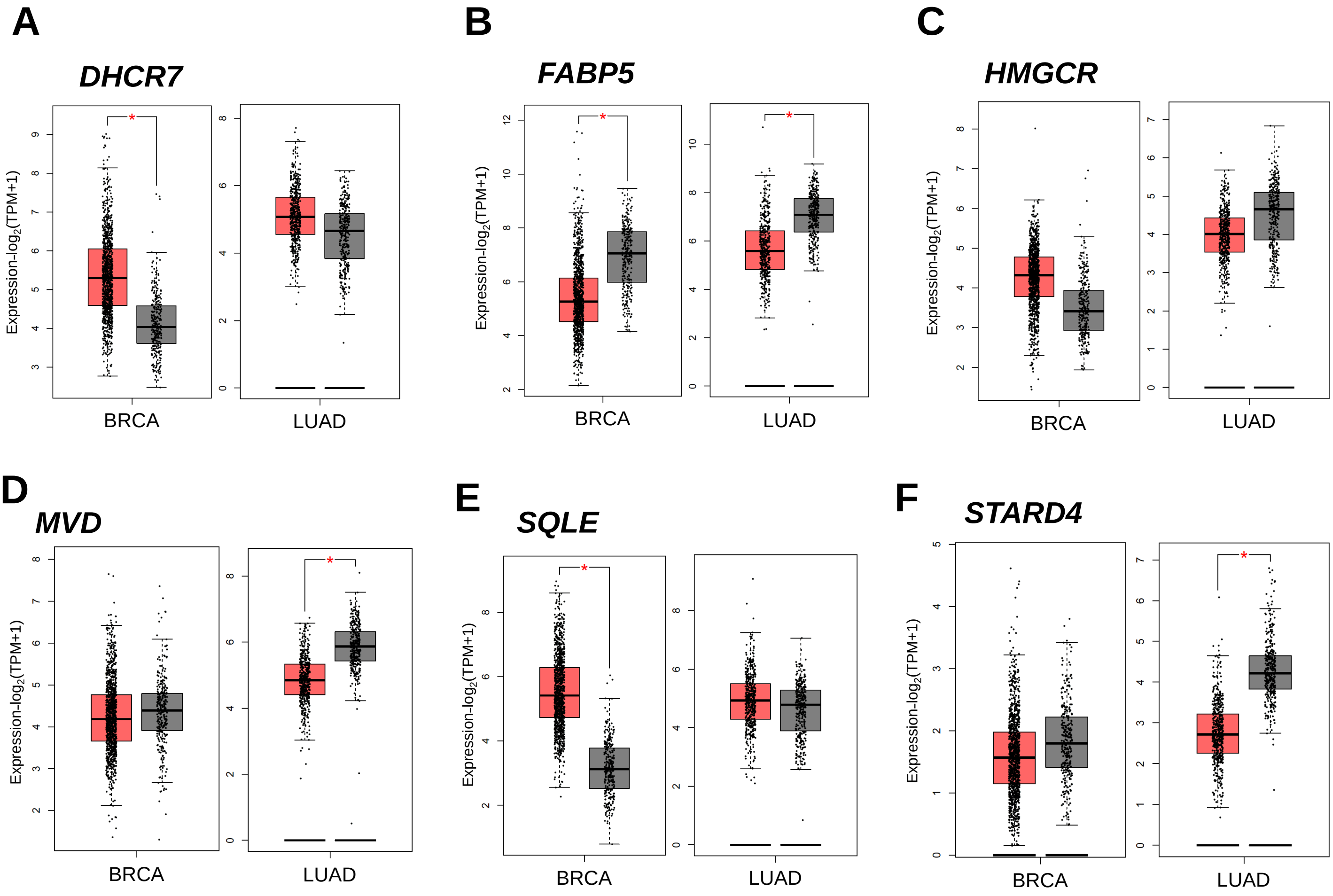

Tumor	N of Tumor samples	N of Normal samples
**BRCA**	1085	291
**LUAD**	483	347

Suppl Fig S7

Supplementary Fig S7. Related to Fig 3. Cholesterol pathway gene expression in BRCA and LUAD. (A-F) Expression levels of the indicated six mRNAs expressed as log_2-transformed transcripts per million (TPM) in tumor or corresponding normal tissue from TCGA datasets of BRCA and LUAD. The table in the bottom indicates the number (N) of tissue samples analyzed in each group. Statistically significant differences are shown based on unpaired student's *t*-test. Red $*p \leq 0.01$.

BRCA

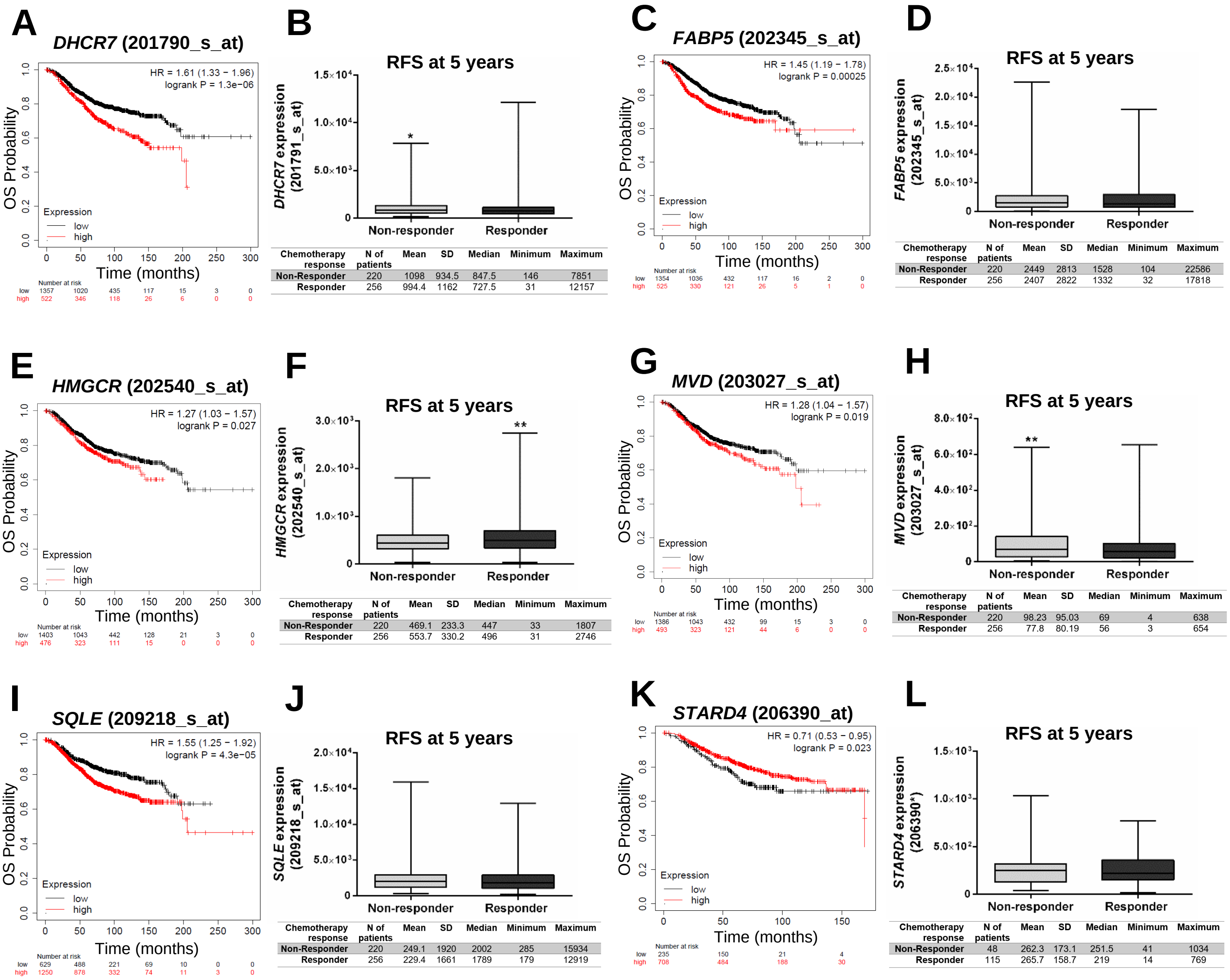

B (DHCR7)

Chemotherapy response	N of patients	Mean	SD	Median	Minimum	Maximum
Non-Responder	220	1098	934.5	847.5	146	7851
Responder	256	994.4	1162	727.5	31	12157

D (FABP5)

Chemotherapy response	N of patients	Mean	SD	Median	Minimum	Maximum
Non-Responder	220	2449	2813	1528	104	22586
Responder	256	2407	2822	1332	32	17818

F (HMGCR)

Chemotherapy response	N of patients	Mean	SD	Median	Minimum	Maximum
Non-Responder	220	469.1	233.3	447	33	1807
Responder	256	553.7	330.2	496	31	2746

H (MVD)

Chemotherapy response	N of patients	Mean	SD	Median	Minimum	Maximum
Non-Responder	220	98.23	95.03	69	4	638
Responder	256	77.8	80.19	56	3	654

J (SQLE)

Chemotherapy response	N of patients	Mean	SD	Median	Minimum	Maximum
Non-Responder	220	249.1	1920	2002	285	15934
Responder	256	229.4	1661	1789	179	12919

L (STARD4)

Chemotherapy response	N of patients	Mean	SD	Median	Minimum	Maximum
Non-Responder	48	262.3	173.1	251.5	41	1034
Responder	115	265.7	158.7	219	14	769

Suppl Fig S8

Supplementary Fig S8. Related to Fig 3. Expression of cholesterol pathway genes correlates with BRCA patient survival and chemotherapy response. (A, C, E, G, I and K) BRCA patient overall survival calculated after sample stratification and comparison in the Kaplan-Meier plotter database using auto select cut-off, based on the low and high expression level of the indicated mRNA. (**B, D, F, H, J and L)** Power of BRCA patient stratification as chemotherapy non-responders or responders based on the expression level of each indicated mRNA and based on the relapse-free survival at five years post-treatment. Each diagram presents median mRNA expression values, along with SD and minimal and maximal values, as explained in the table below the graph, which also presents the number (N) of analyzed patients. Comparisons were performed with Mann–Whitney U-test (*$p \leq 0.05$; **$p \leq 0.01$).

LUNG Cancer

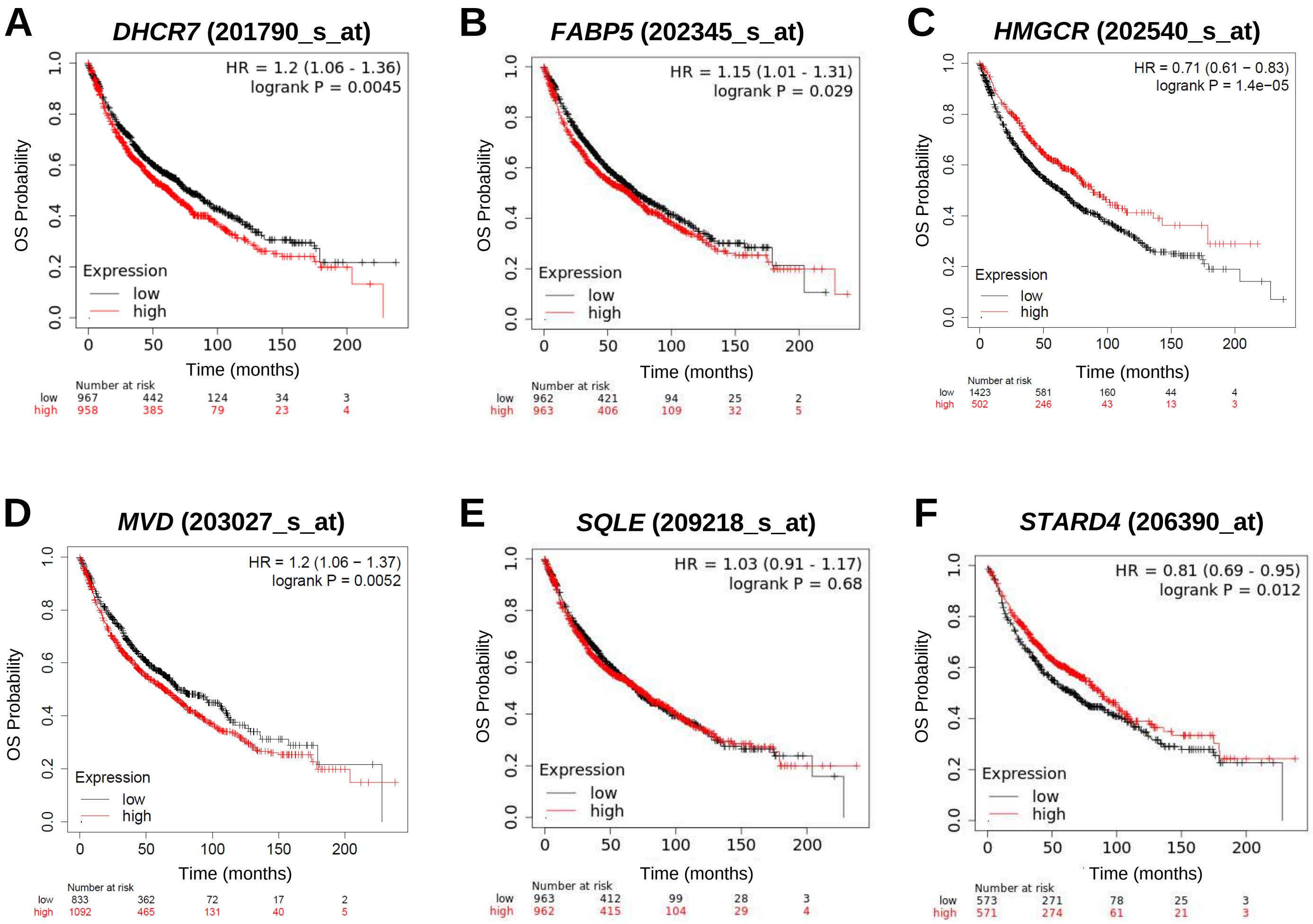

Suppl Fig S9

Supplementary Fig S9. Related to Fig 3. Expression of cholesterol pathway genes correlates with lung cancer patient survival. (A-F) Lung cancer overall survival calculated after sample stratification and comparison in the Kaplan-Meier plotter database using auto select cut-off, based on the low and high expression level of the indicated mRNAs.

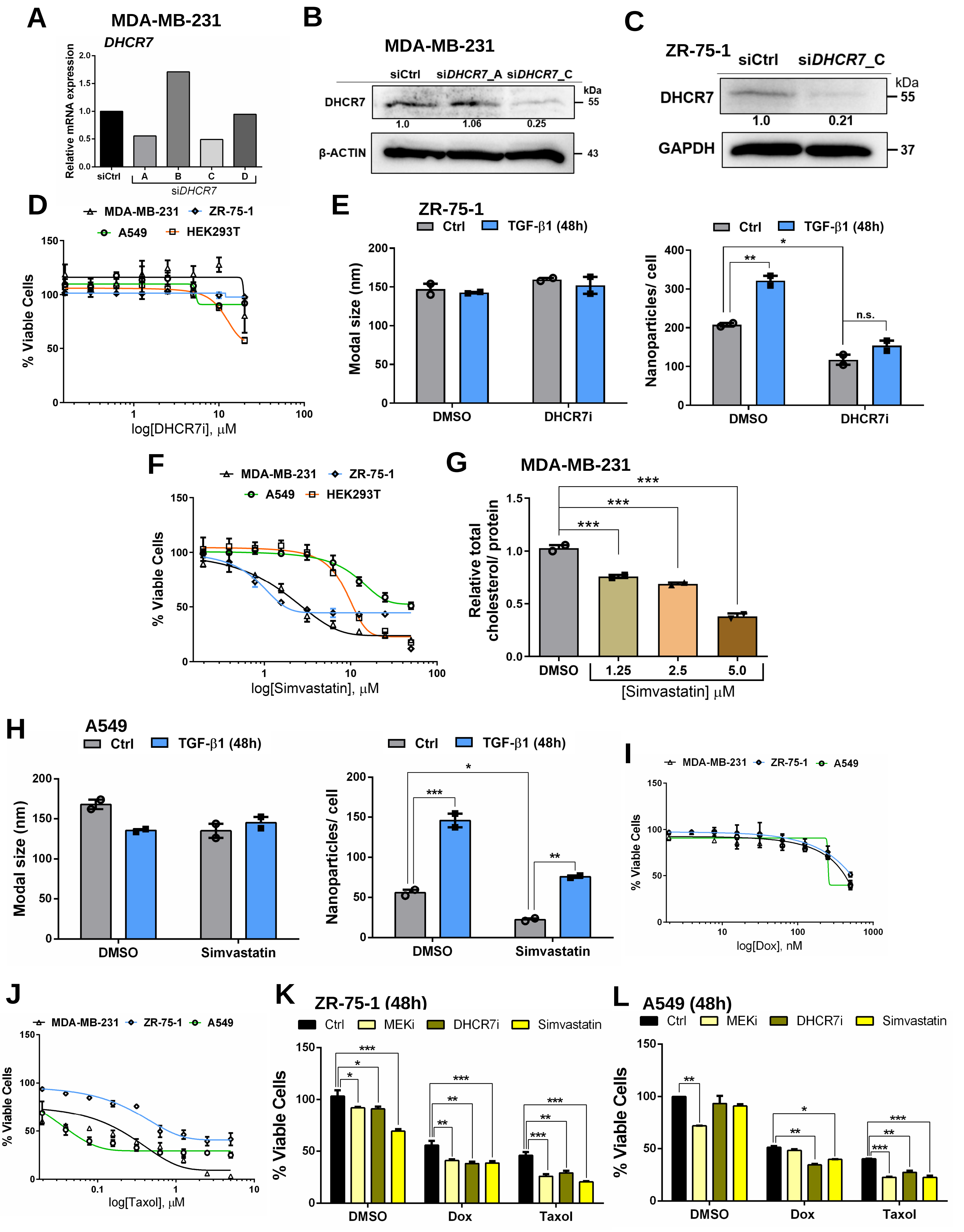

Suppl Fig S10

Supplementary Fig S10. Related to Fig 3. TGF-β induces EV release via DHCR7 and the cholesterol pathway. (A) RT-qPCR analysis of *DHCR7* mRNA levels in MDA-MB-231 cells after transient transfection with control (Ctrl) or four specific (*A-D*) siRNAs. Values represent fold-change of mRNA expression normalized to *GAPDH* and expressed relative to the level at siCtrl. **(B)** Expression levels of DHCR7 protein and β-ACTIN serving as loading control from MDA-MB-231 cells transiently transfected with two independent siRNAs for 48 h. **(C)** Expression levels of DHCR7 protein in ZR-75-1 cells transiently transfected with control (siCtrl) or specific siRNA targeting *DHCR7* (DHCR7_C). Densitometric values in B and C were normalized to the control siRNA. B and C present representative immunoblots of two independent biological replicates along with molecular mass markers in kDa. **(D)** Viability curves of the four indicated cell models in the presence of increasing concentrations of the DHCR7i (logarithmic scale). The data are presented as mean values of three biological replicates ± SEM, each in technical triplicates. Note the lack of statistically significant differences. **(E)** EVs released by ZR-75-1 cells quantified by NTA in terms of particle size (left) and particle number after normalization to the total cell number (right). The cells were stimulated with vehicle (Ctrl) or 5 ng/mL TGF-β1 in the absence or presence of 20 μM DHCR7i for 48 h. **(F)** Viability curves of the four indicated cell models in the presence of increasing concentrations of simvastatin (logarithmic scale). The data are presented as mean values of three biological replicates ± SEM, each in technical triplicates. **(G)** Quantification of total cholesterol levels in MDA-MB-231 cells in the presence of 1.25, 2.5 or 5 μM simvastatin for 48 h. **(H)** EVs released by A549 cells quantified by NTA in terms of particle size (left) and particle number after normalization to the total cell number (right). The cells were stimulated with vehicle (Ctrl) or 5 ng/mL TGF-β1 in the absence or presence of 5.0 μM simvastatin for 48 h. **(I, J)** Cell viability curves of the three indicated cell models in the presence of increasing concentrations of doxorubicin (Dox, I) or paclitaxel (taxol) (J) (logarithmic scale). The data are presented as mean values of three biological replicates ± SEM, each in technical triplicates. **(K, L)** Cell viability assay with ZR-75-1 (K) and A549 (L) cells incubated with vehicle (DMSO), 5 μM MEKi, 20 μM DHCR7i or 1.25 μM simvastatin for 48 h, in the presence of co-incubation with DMSO or 0.5 μM Dox or 0.25 μM Taxol. The cholesterol level data in G are presented as mean values of two biological replicates ± SEM, in technical triplicates and p-values are shown based on one-way ANOVA, followed by multiple paired comparisons conducted by means of Bonferroni's post-test method. The EV/NTA data (panels E and H) are presented as mean values of two biological replicates ± SEM, each in technical duplicates and p-values are shown based on two-way ANOVA, followed by multiple paired comparisons conducted by means of Bonferroni's post-test method. The cell viability data in K and L are presented as mean values of three biological replicates ± SEM, in technical triplicates and p-values are shown based on two-way ANOVA, followed by multiple paired comparisons conducted by means of Bonferroni's post-test method. P-values: *p ≤ 0.05; **p ≤ 0.01; ***p ≤ 0.001; n.s., not significant.

A

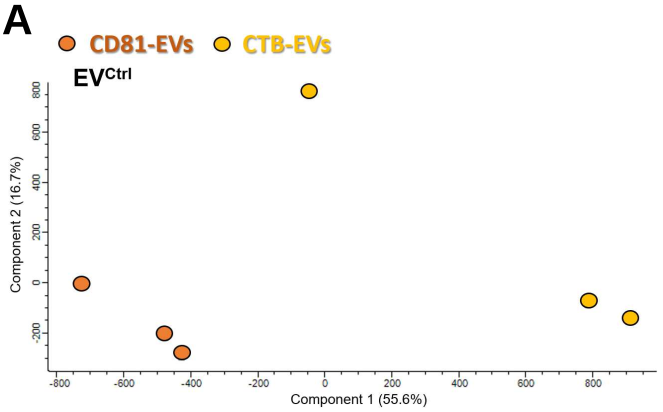

B

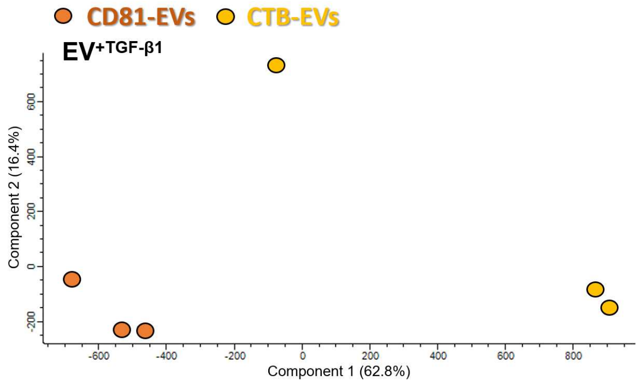

C

CD81-EVs: 539 common proteins

Source	Term description	FDR q-value
GO: BP	Cellular component organization	4.15E-25
GO: BP	Cellular process	4.41E-25
GO: BP	Cellular component organization or biogenesis	1.15E-24
GO: BP	Protein folding	5.53E-21
GO: BP	Cellular component assembly	5.53E-21
GO: MF	Protein binding	1.21E-57
GO: MF	RNA binding	6.70E-50
GO: MF	Cell adhesion molecule binding	1.02E-45
GO: MF	Binding	1.52E-43
GO: MF	Cadherin binding	1.24E-37
GO: CC	Extracellular exosome	3.95E-161
GO: CC	Extracellular vesicle	3.95E-161
GO: CC	Extracellular space	2.38E-143
GO: CC	Extracellular region	2.46E-123
GO: CC	Vesicle	3.65E-123
REAC	Infectious disease	5.28E-35
REAC	Metabolism of proteins	2.14E-32
REAC	Disease	2.14E-29
REAC	Axon guidance	2.39E-25
REAC	Immune system	5.61E-24

D

CTB-EVs: 1797 common proteins

Source	Term description	FDR q-value
GO: BP	Cellular process	2.15E-60
GO: BP	Organic substance metabolic process	1.20E-54
GO: BP	Metabolic process	1.03E-53
GO: BP	Nitrogen compound metabolic process	1.69E-53
GO: BP	Primary metabolic process	1.08E-49
GO: MF	Protein binding	7.81E-90
GO: MF	Binding	2.10E-74
GO: MF	Cell adhesion molecule binding	4.35E-73
GO: MF	RNA binding	7.04E-66
GO: MF	Cadherin binding	1.76E-63
GO: CC	Extracellular exosome	5.42E-283
GO: CC	Extracellular vesicle	1.39E-282
GO: CC	Extracellular space	6.50E-250
GO: CC	Extracellular region	1.17E-219
GO: CC	Vesicle	3.55E-211
REAC	Metabolism of proteins	7.01E-63
REAC	Disease	1.25E-54
REAC	Infectious disease	1.25E-54
REAC	Immune system	3.56E-51
REAC	Neutrophil degranulation	4.91E-51

E

CTB-EVs^{Ctrl}

Source	Term description	FDR q-value
GO: BP	Nitrogen compound metabolic process	4.08E-11
GO: BP	Metabolic process	4.08E-11
GO: BP	Primary metabolic process	4.08E-11
GO: BP	Cellular metabolic process	6.22E-11
GO: BP	Organic substance metabolic process	1.22E-10
GO: BP	Macromolecule metabolic process	3.14E-08
GO: BP	Cellular process	1.62E-07
GO: MF	RNA binding	6.72E-10
GO: MF	Protein binding	4.62E-05
GO: MF	Catalytic activity	0.0012
GO: MF	mRNA binding	0.0057
GO: MF	Heparin binding	0.0208
GO: CC	Cytosol	1.53E-10
GO: CC	Protein-containing complex	8.78E-09
GO: CC	Membrane-bounded organelle	3.71E-08
GO: CC	Extracellular exosome	3.71E-08
GO: CC	Extracellular vesicle	3.71E-08
REAC	Metabolism of RNA	1.07E-06
REAC	mRNA Splicing - Major Pathway	2.78E-06
REAC	Processing of Pre-mRNA	1.78E-05
REAC	Metabolism of proteins	0.00079
REAC	Cellular responses to stress	0.005

F

CTB-EVs$^{+TGF\text{-}\beta 1}$

Source	Term description	FDR q-value
GO: BP	Negative regulation of biological process	9.69E-05
GO: BP	TGF-β receptor signaling pathway	0.00023
GO: BP	Catabolic process	0.00046
GO: BP	Macromolecule catabolic process	0.00046
GO: BP	Cellular response to TGF-β stimulus	0.00079
GO: BP	RNA catabolic process	0.00086
GO: BP	Cellular response to growth factor stimulus	0.00086
GO: MF	Binding	1.00E-05
GO: MF	Protein binding	0.00017
GO: MF	Enzyme binding	0.0231
GO: MF	Protein-containing complex binding	0.0357
GO: MF	Protein phosphatase binding	0.0444
GO: CC	Cytoplasm	2.68E-08
GO: CC	Cytosol	5.98E-06
GO: CC	Extracellular region	9.23E-05
GO: CC	Anchoring junction	9.80E-05
GO: CC	Extracellular vesicle	0.0111
REAC	Disease	0.012
REAC	Extracellular matrix organization	0.0126
REAC	Metabolism of nucleotides	0.0126
REAC	Cellular responses to stress	0.0126
REAC	Deadenylation-dependent mRNA decay	0.0126

Suppl Fig S11

Supplementary Fig S11. Related to Fig 4. Comparison of two different EV subpopulations based on their protein cargo. (A, B) PCA of statistically significant protein expression differences between EVs enriched by the CD81-specific and the CTB-specific method in the VSF of MDA-MB-231 cells stimulated with vehicle (Ctrl) (A) or 5 ng/mL TGF-β1 for 120 h (B). Three independent biological repeats were analyzed per condition. **(C, D)** Tables of highly significant gene ontology (GO) and Reactome (REAC) terms represented in the two biological conditions analyzed using the proteins commonly present in CD81- (C) or CTB-EVs (D) isolated from MDA-MB-231 cells stimulated with vehicle (Ctrl) or 5 ng/mL TGF-β1 for 120 h, indicating the term name and associated false discovery rate (FDR) q-value. **(E, F)** Tables of highly significant gene ontology (GO) and Reactome (REAC) terms represented in the two biological conditions (Control (E) and TGF-β1 (F)) analyzed using the unique proteins carried by CTB-EV, indicating the term name and associated false discovery rate (FDR) q-value.

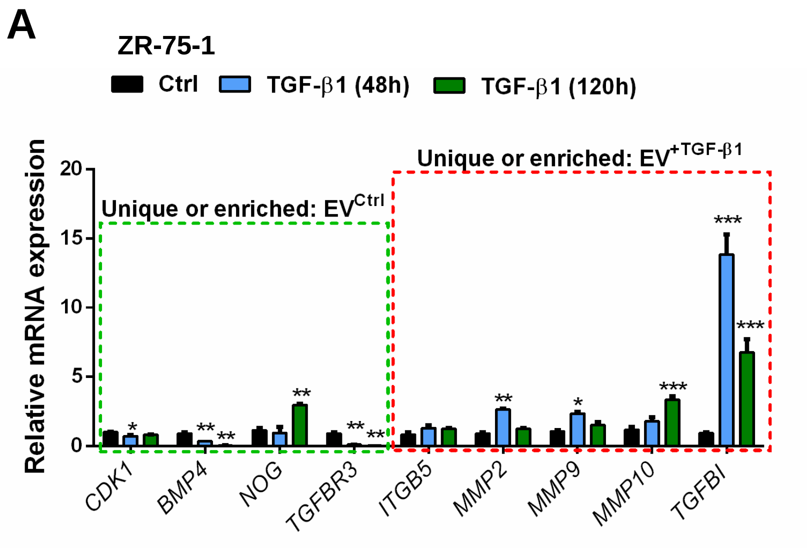

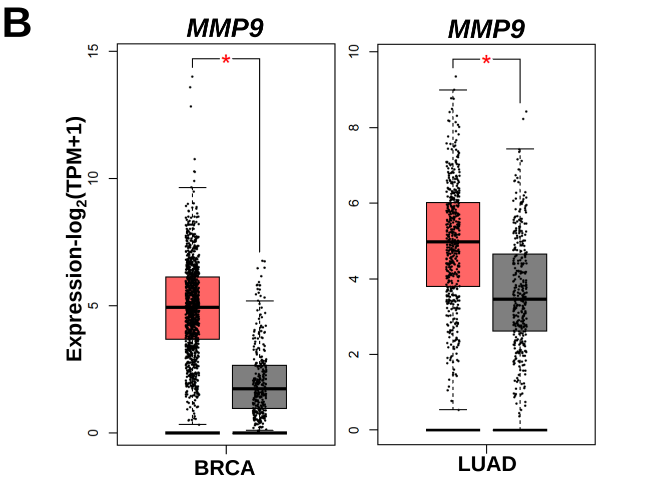

Tumor	N of Tumor samples	N of Normal samples
BRCA	1085	291
LUAD	483	347

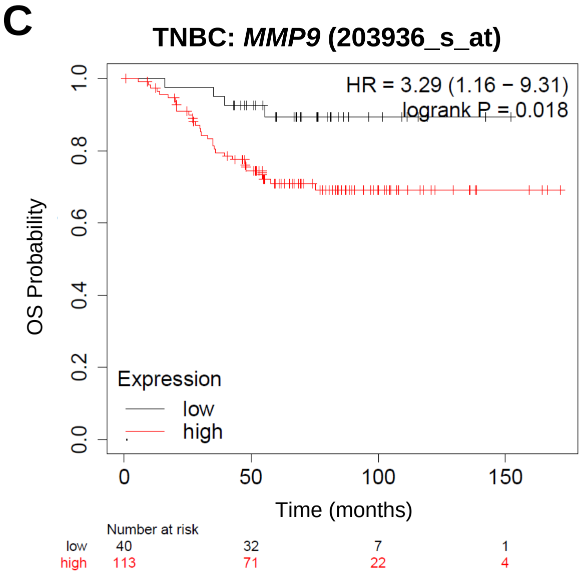

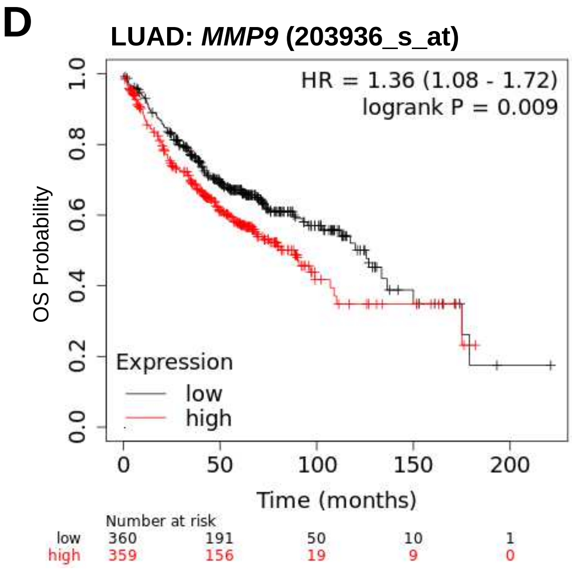

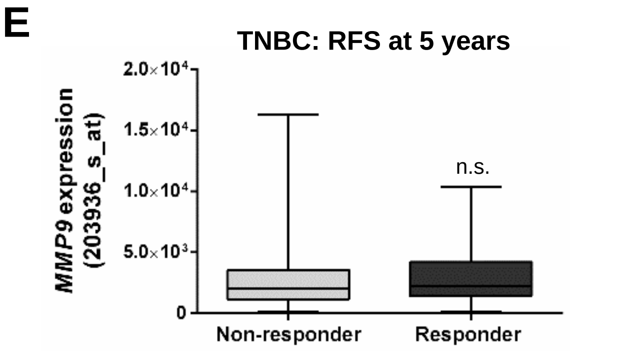

Chemotherapy response	N of patients	Mean	SD	Median	Minimum	Maximum
Non-Responder	84	2914	2842	2079	164	16342
Responder	80	3065	2338	2243	143	10408

Suppl Fig S12

Supplementary Fig S12. Related to Fig 4. The EV cargo proteome includes TGF-β signaling and metalloprotease proteins with clinical relevance. (A) RT-qPCR analysis of the indicated mRNAs, selected based on the corresponding proteins that scored significantly in the proteomic analysis of MDA-MB-231 cells, analyzed in ZR-75-1 cells after stimulation with 5 ng/mL TGF-β1 for 0 (Ctrl), 48 and 120 h. The dotted rectangles indicate groups of mRNAs, whose respective proteins were unique or enriched in control (Ctrl) or TGF-β1-stimulated EV populations. Values represent fold-change of mRNA expression normalized to *GAPDH* and expressed relative to the level at Ctrl. The data are presented as mean values of three biological replicates ± SEM, in technical triplicates and p-values are shown based on two-way ANOVA, followed by multiple paired comparisons conducted by means of Bonferroni's post-test method: *p ≤ 0.05; **p ≤ 0.01; ***p ≤ 0.001. **(B)** Levels of *MMP9* mRNA expressed as log_2-transformed transcripts per million (TPM) in tumor (red) or corresponding normal (grey) tissue from TCGA datasets of BRCA and LUAD. The numbers in the bottom indicate the number (N) of tissue samples analyzed in each group. Statistically significant differences are shown based on unpaired student's *t*-test. P-values: *p ≤ 0.01. **(C, D)** TNBC (C) and LUAD (D) patient overall survival calculated after sample stratification and comparison in the Kaplan-Meier plotter database using auto select cut-off, based on the low and high expression level of the *MMP9* mRNA. **(E)** Power of TNBC patient stratification as chemotherapy non-responders or responders based on the expression level of each indicated mRNA and based on the relapse-free survival at five years post-treatment. Each diagram presents median mRNA expression values, along with SD and minimal and maximal values, as explained in the table below the graph, which also presents the number (N) of analyzed patients. Comparisons were performed with Mann–Whitney U-test (n.s., not significant).

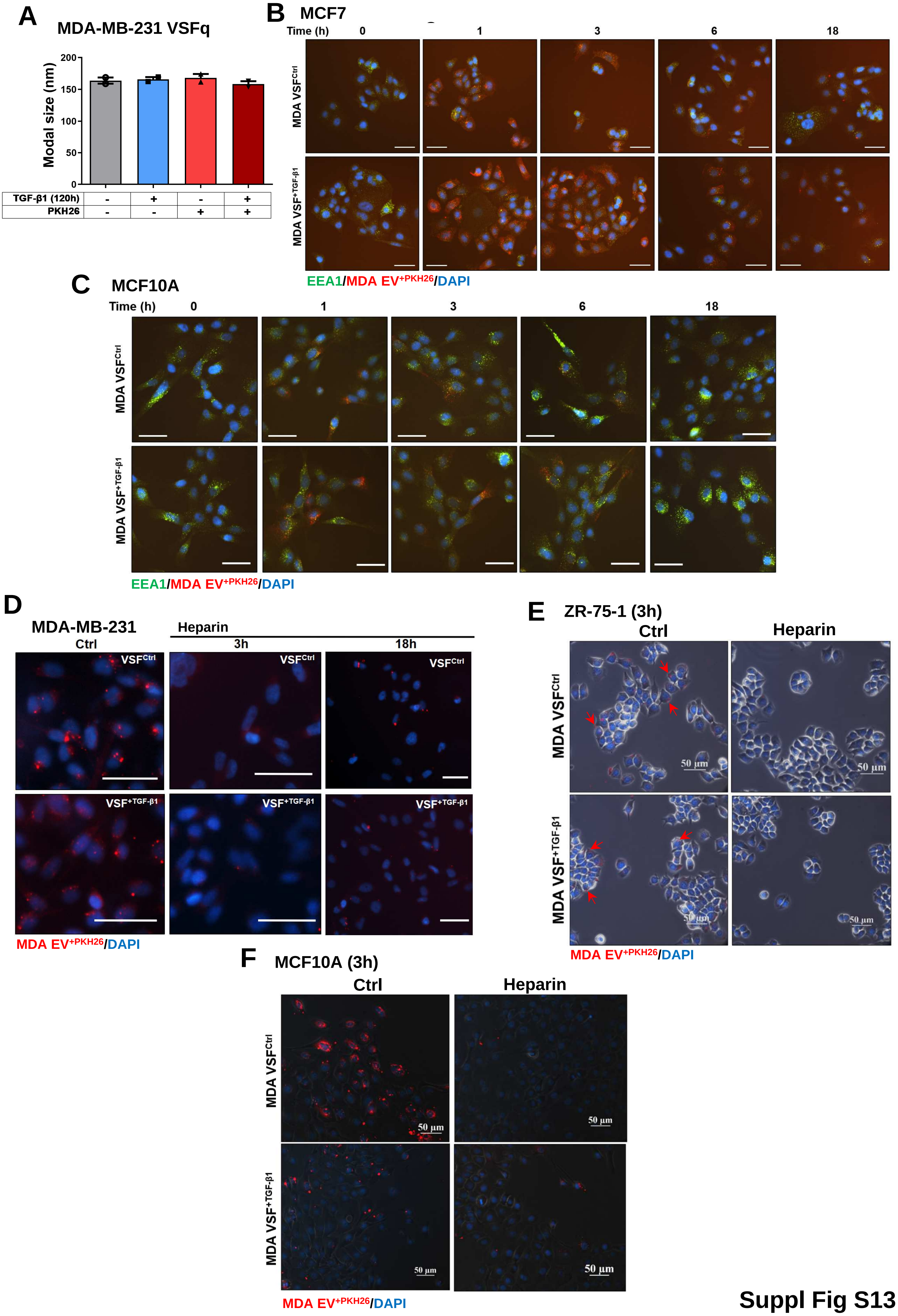

Suppl Fig S13

Supplementary Fig S13. Related to Fig 5. Characterization of EV and recipient cell interaction. (A) EVs released by MDA-MB-231 cells (VSF) and fluorescently labelled with PKH26 retain their biophysical properties. NTA of particle size in EVs isolated from cells that were stimulated with vehicle (-) or 5 ng/mL TGF-β1 for 120 h and then remained intact or fluorescently labeled with PKH26. The data are presented as mean values of three biological replicates ± SEM, each in technical duplicates. Note the lack of significant differences. **(B, C)** Representative immunofluorescence microscopy pictures of MCF7 (B) and MCF10A (C) cells incubated for 1, 3, 6, 18 h or not (0 h) with EVs derived from control MDA-MB-231 cells (MDA VSF^{Ctrl}) or cells stimulated with 5 ng/mL TGF-β1 for 120 h (MDA $VSF^{+TGF\text{-}\beta1}$). The early endosome protein EEA1 (green), the EVs (red) and nuclei (blue) are labeled. **(D)** Representative immunofluorescence microscopy pictures of MDA-MB-231 cells incubated with EVs for 3 or 18 h in the presence of vehicle (Ctrl) or 50 μg/mL heparin. The EVs (red) and nuclei (DAPI: blue) are labeled. Scale bars, 50 μm. **(E, F)** Representative immunofluorescence microscopy pictures of ZR-75-1 (E) and MCF10A (F) cells incubated with EVs derived from MDA-MB-231 (MDA) cells for 3 h, in the presence of vehicle (Ctrl) or 50 μg/mL heparin. The EVs (red) and nuclei (blue) are labeled. Scale bars, 50 μm.

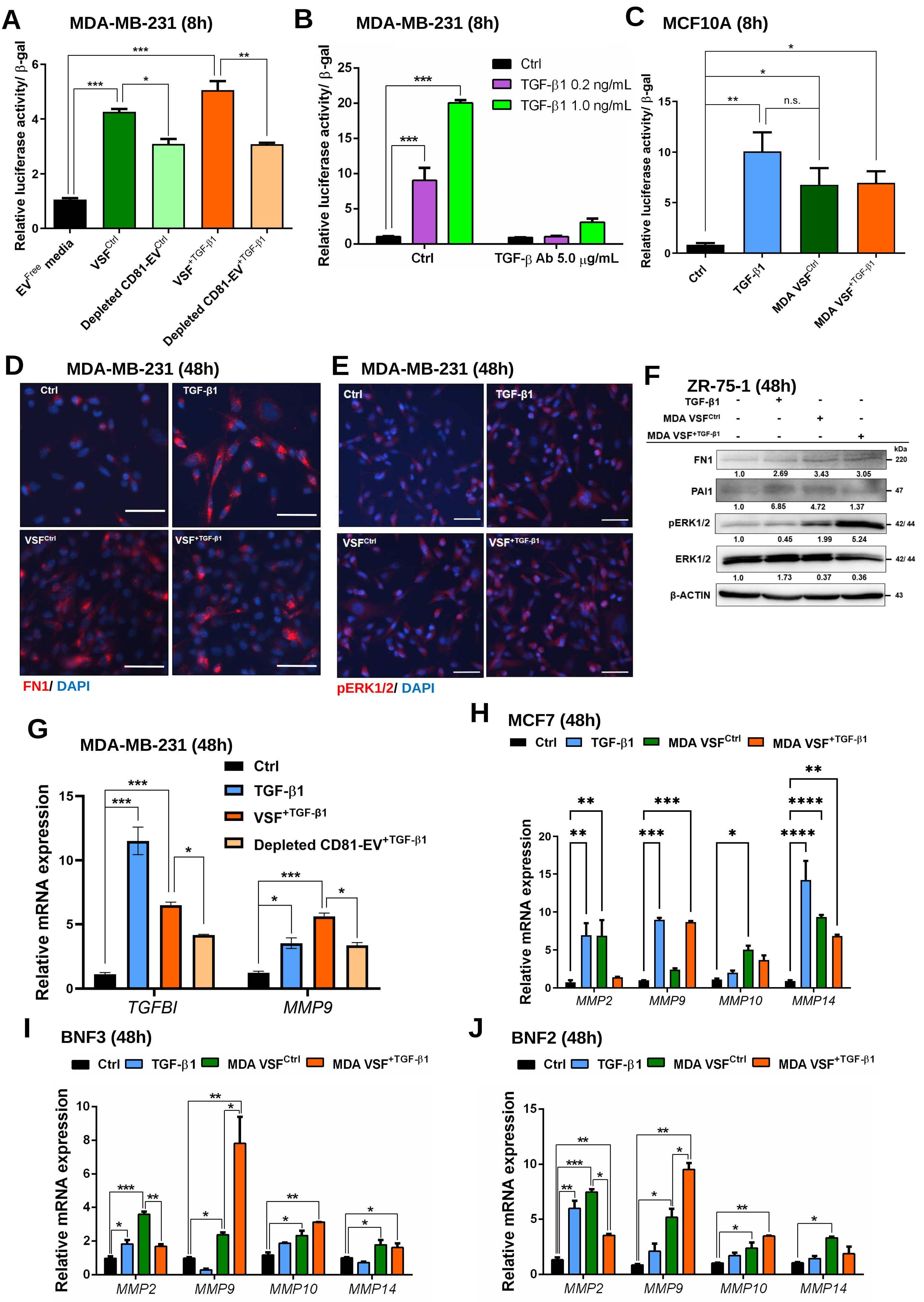

Suppl Fig S14

Supplementary Figure S14. Related to Figure 5. EVs activate TGF-β signaling and *MMP* gene family expression using surface-associated proteins and MMP activity-dependent function. (A) Relative luciferase activity generated in MDA-MB-231 cells by transfecting the TGF-β-inducible $CAGA_{12}$-luc reporter, normalized to β-galactosidase activity generated by a co-transfected reporter, incubated for 8 h with EV-free media, 1×10^9 nanoparticles of EVs derived from control cells (VSF^{Ctrl}), supernatant depleted from CD81-positive EV^{Ctrl}, TGF-β1-induced EVs ($VSF^{+TGF\text{-}\beta1}$) or supernatant depleted from CD81-positive $EV^{+TGF\text{-}\beta1}$. **(B)** Relative $CAGA_{12}$-luciferase activity generated in MDA-MB-231 cells normalized to β-galactosidase activity, after stimulation of the transfected cells with the indicated amounts of TGF-β1, in the presence of control IgG (Ctrl) or 5 μg/mL anti-TGF-β neutralizing antibody for 8 h. Data are presented as mean values of three biological replicates ± SEM, each in technical duplicates and p-values are shown based on two-way ANOVA, followed by multiple paired comparisons conducted by means of Bonferroni's post-test method. **(C)** Relative luciferase activity generated in MCF10A cells by transfecting the TGF-β-inducible $CAGA_{12}$-luc reporter, normalized to β-galactosidase activity generated by a co-transfected reporter, after stimulation of the transfected cells with 5 ng/mL TGF-β1, or incubation with 1×10^9 nanoparticles of VSF^{Ctrl} or $VSF^{+TGF\text{-}\beta1}$ from MDA cells for 8 h. **(D, E)** Representative immunofluorescence microscopy pictures of MDA-MB-231 (MDA) cells stimulated with vehicle (Ctrl), 5 ng/mL TGF-β1, or 1×10^9 nanoparticles of EVs from control cells (VSF^{Ctrl}) or stimulated with TGF-β1 for 120 h ($VSF^{+TGF\text{-}\beta1}$) for 48 h. The ECM protein fibronectin (FN1, red) (A), the phospho-ERK1/2 proteins (pERK1/2, red) (B) and nuclei (blue) are labeled, whereas the EVs were not labeled. Scale bars, 50 μm. **(F)** Expression levels of the indicated proteins in cellular extracts of ZR-75-1 cells stimulated or not with 5 ng/mL TGF-β1 or incubated with 1×10^9 nanoparticles of MDA VSF^{Ctrl} or $VSF^{+TGF\text{-}\beta1}$, for 48 h; densitometric values were normalized to the control (-) condition. Representative immunoblots of two independent biological replicates along with molecular mass markers in kDa are shown. **(G)** RT-qPCR analysis of the indicated mRNAs in MDA cells after stimulation or not (Ctrl) with 5 ng/mL TGF-β1 for 48 h or incubation with 1×10^9 nanoparticles of MDA $VSF^{+TGF\text{-}\beta1}$ or supernatant depleted from CD81-positive $EV^{+TGF\text{-}\beta1}$ for 48 h. Values represent fold-change of mRNA expression normalized to *GAPDH* and expressed relative to the level of Ctrl. **(H-J)** RT-qPCR analysis of the indicated mRNAs in MCF7 (H) and in the normal breast fibroblasts BNF3 (I) and BNF2 (J) after stimulation or not (Ctrl) with 5 ng/mL TGF-β1 for 48 h or incubation with equal number of MDA VSF^{Ctrl} or $VSF^{+TGF\text{-}\beta1}$ for 48 h. Values represent fold-change of mRNA expression normalized to *GAPDH* and expressed relative to the level of Ctrl. Data in A and C are presented as mean values of three biological replicates ± SEM, each in technical duplicates and p-values are shown based on one-way ANOVA, followed by multiple paired comparisons conducted by means of Bonferroni's post-test method. Data in B, G-J are presented as mean values of three biological replicates ± SEM, each in technical duplicates and p-values are shown based on two-way ANOVA, followed by multiple paired comparisons conducted by means of Bonferroni's post-test method. P-values: *p ≤ 0.05; **p ≤ 0.01; ***p ≤ 0.001; **** p ≤ 0.0001; n.s., not significant.

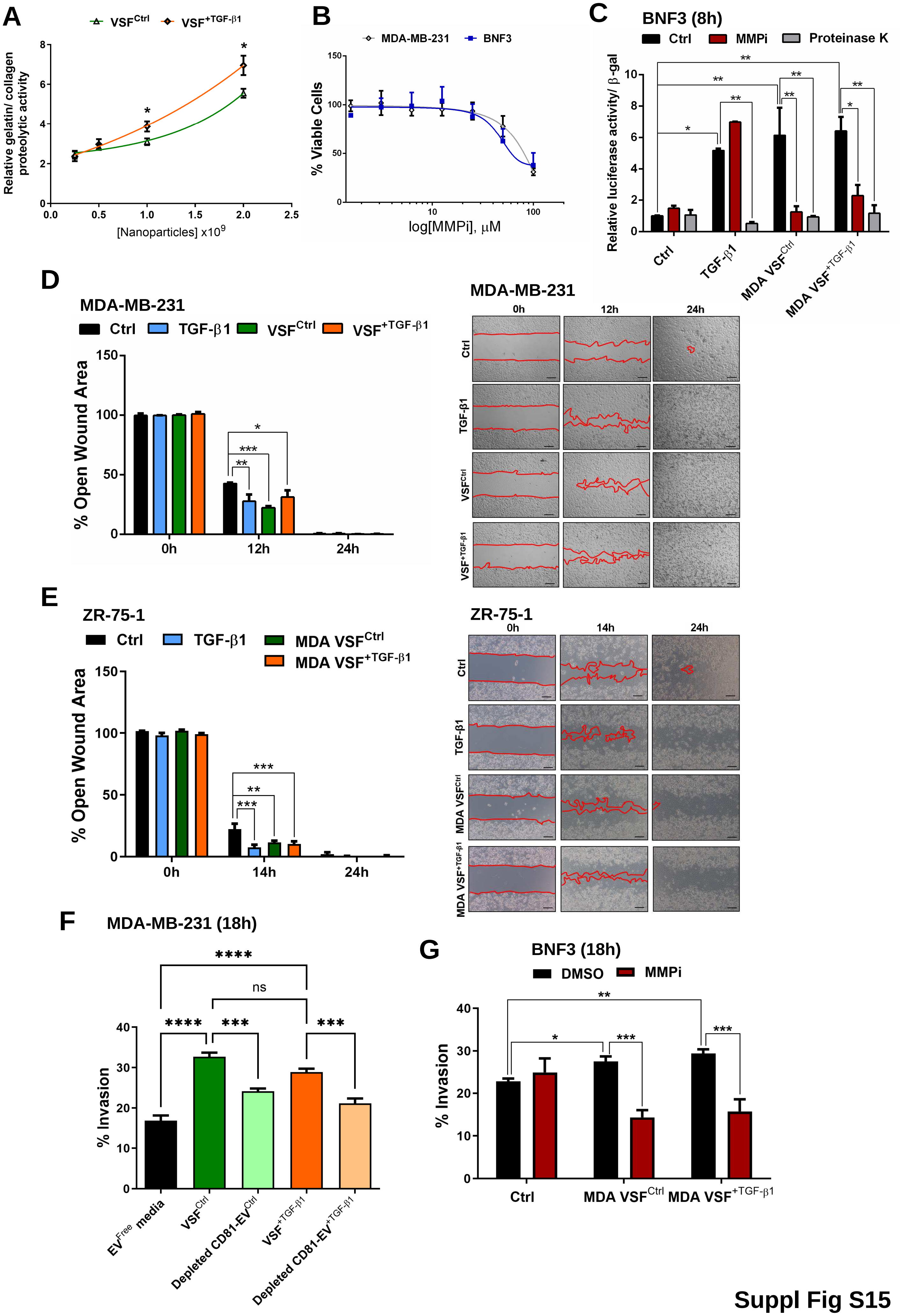

Suppl Fig S15

Supplementary Fig S15. Related to Fig 5. EVs induce an invasive phenotype in breast carcinoma, non-tumorigenic breast epithelial or normal breast fibroblastic cells. (A) Relative MMP proteolytic activity was measured using the EnzChek Gelatinase/Collagenase Assay kit with increasing numbers of nanoparticles (5×10^7, 1×10^8 and 1×10^9) from VSF^{Ctrl} or $VSF^{+TGF\text{-}\beta1}$. The data are presented as mean values of three biological replicates ± SEM, in technical triplicates and p-values are shown based on unpaired student's *t*-test *p ≤ 0.05. **(B)** Cell viability curves of MDA-MB-231 (MDA) and BNF3 cells in the presence of increasing concentrations of the MMP inhibitor (MMPi) (logarithmic scale). The data are presented as mean values of three biological replicates ± SEM, each in technical duplicates. Note the lack of statistically significant differences. **(C)** Relative luciferase activity generated in BNF3 cells by transfecting the TGF-β-inducible $CAGA_{12}$-luc reporter, normalized to β-galactosidase activity generated by a co-transfected reporter, after stimulation of the transfected cells with vehicle (Ctrl), 5 ng/mL TGF-β1, or incubation with 1×10^9 nanoparticles of MDA VSF^{Ctrl} or $VSF^{+TGF\text{-}\beta1}$ for 8 h. The EVs were pre-treated with vehicle DMSO (Ctrl), 25 μM MMPi or 40 μg/ml proteinase K for 30 min. **(D, E)** Cell culture wound healing assay with MDA (D) and ZR-75-1 (E) cells incubated with vehicle (Ctrl), 5 ng/mL TGF-β1 or with 1×10^9 nanoparticles of control EVs (VSF^{Ctrl}) or TGF-β1-induced EVs ($VSF^{+TGF\text{-}\beta1}$) from MDA cells for the indicated time periods. Representative microscopy pictures of MDA-MB-231 (D) and ZR-75-1 (E) are shown. Scale bars, 200 μm. The data are presented as percent open wound area and plotted as mean values of three biological replicates ± SEM, in technical triplicates and p-values are shown based on two-way ANOVA, followed by multiple paired comparisons conducted by means of Bonferroni's post-test method: *p ≤ 0.05; **p ≤ 0.01; ***p ≤ 0.001. **(F)** Matrigel invasion assay in trans-wells with MDA-MB-231 cells stimulated with EV-free media, 1×10^9 nanoparticles of VSF^{Ctrl}, supernatant depleted from CD81-positive EV^{Ctrl}, $VSF^{+TGF\text{-}\beta1}$ or supernatant depleted from CD81-positive $EV^{+TGF\text{-}\beta1}$ for 48 h. **(G)** Matrigel invasion assay in trans-wells with normal breast BNF3 cells incubated with vehicle (Ctrl) and equal number of MDA EVs (VSF^{Ctrl} or $VSF^{+TGF\text{-}\beta1}$) for 48 h, in the presence of vehicle (DMSO) or 25 μM MMP inhibitor (MMPi). The data in F and G represent the invaded cells as percent of the total cell number and are plotted as mean values of three biological replicates ± SEM, in technical triplicates. P-values in F are shown based on one-way ANOVA, while in C-E and G are shown as two-way ANOVA, followed by multiple paired comparisons conducted by means of Bonferroni's post-test method: *p ≤ 0.05; **p ≤ 0.01; ***p ≤ 0.001; ****p ≤ 0.0001; ns., not significant.

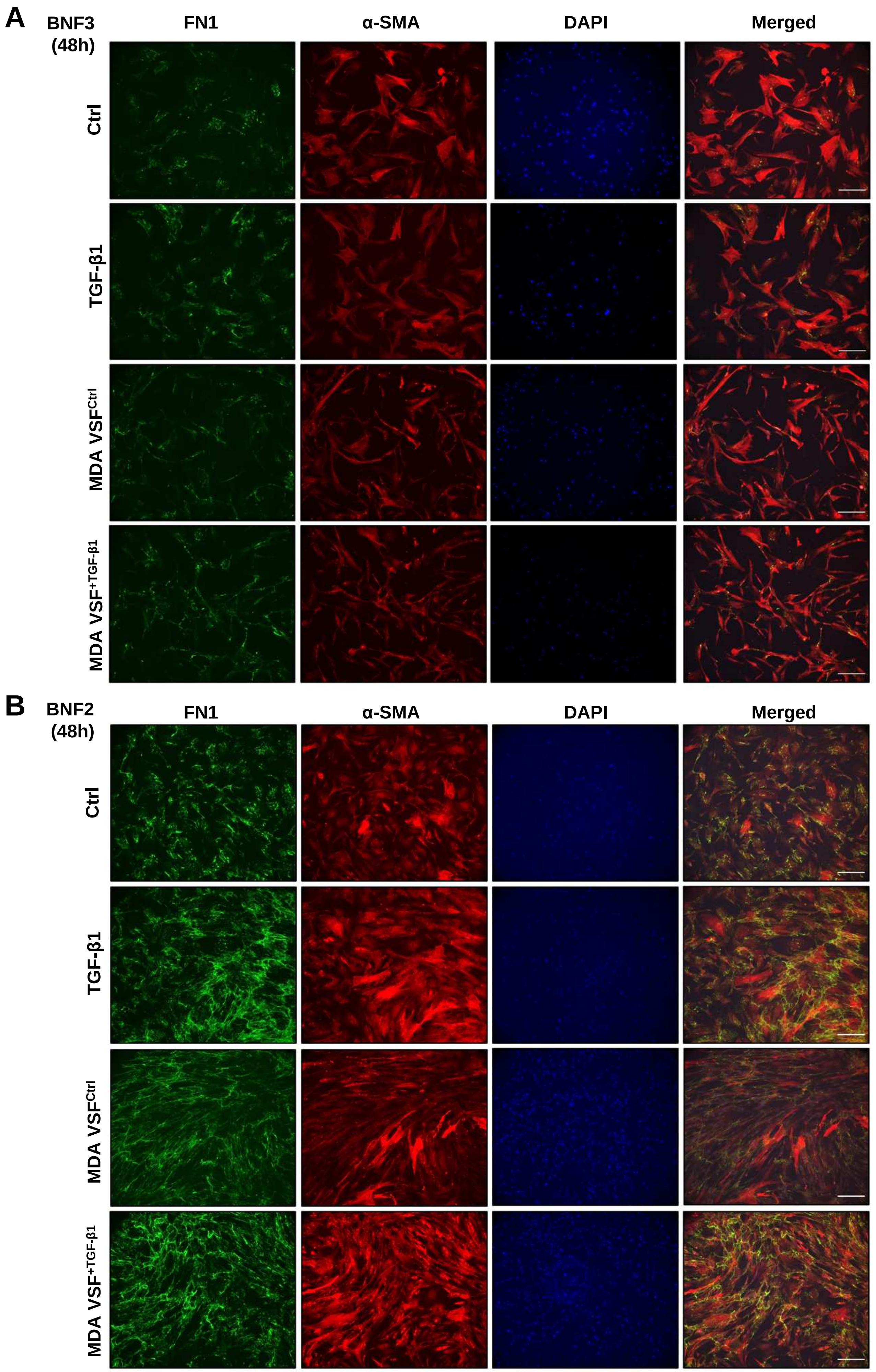

Suppl Fig S16

Supplementary Fig S16. Related to Fig 5. EVs induce an invasive phenotype in normal breast fibroblastic cells. (A, B) Representative immunofluorescence microscopy pictures of BNF3 (A) and BNF2 (B) normal breast fibroblasts cells stimulated with vehicle (Ctrl) or 5 ng/mL TGF-β1, or incubated with equal number of MDA VSF^{Ctrl} or $VSF^{+TGF\text{-}\beta1}$ for 48 h. The ECM protein fibronectin (FN1, green) and the cytoskeletal protein α-SMA (red) are labeled, whereas the EVs were not labeled. Scale bars, 100 μm.

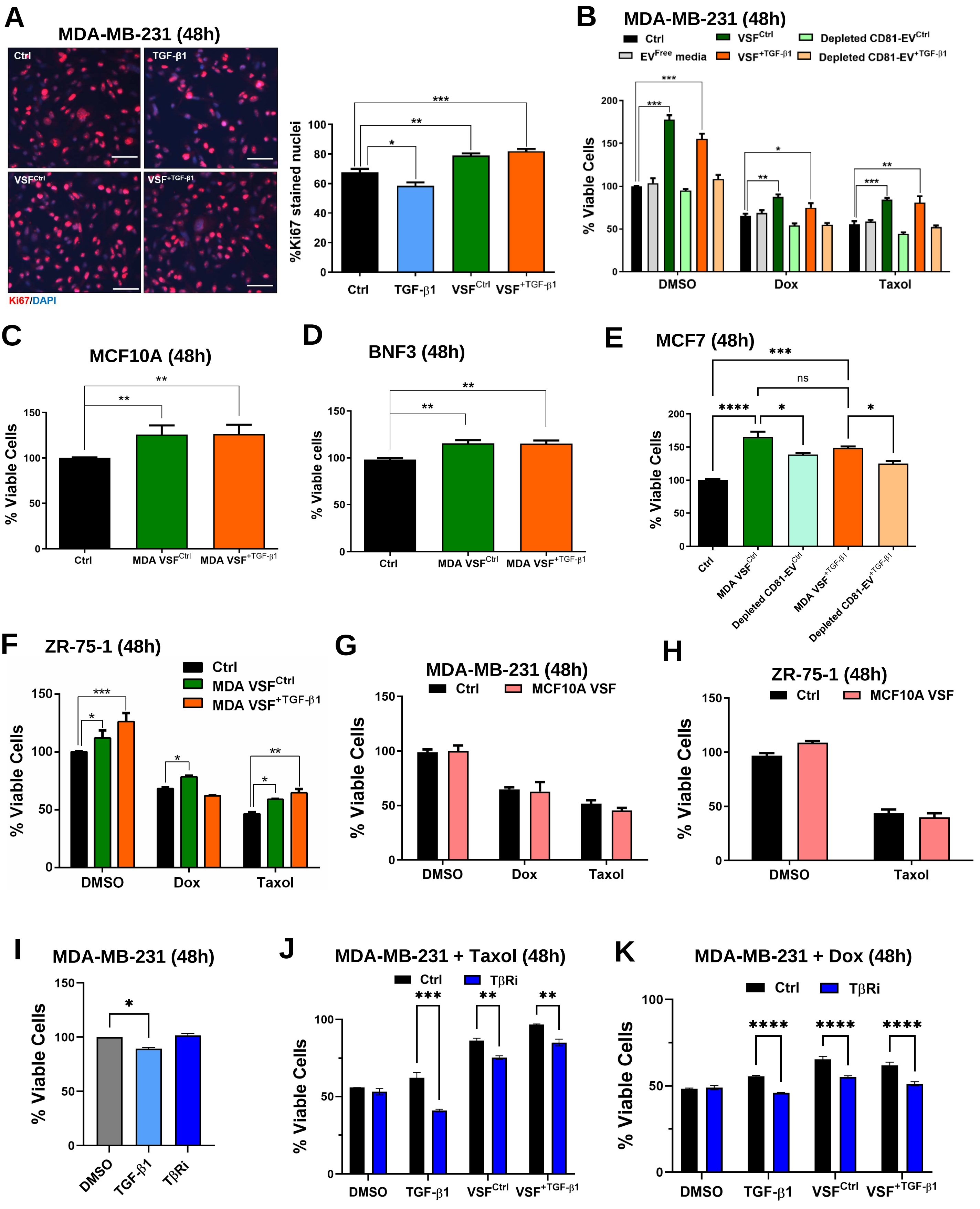

Suppl Fig S17

Supplementary Fig S17. Related to Fig 5. Viability assays in breast cancer cells exposed to EVs and clinically used cytotoxic drugs. (A) Representative immunofluorescence microscopy pictures of MDA-MB-231 cells stimulated with vehicle (Ctrl), 5 ng/mL TGF-β1, or incubated with 1×10^9 nanoparticles of VSF^{Ctrl} or $VSF^{+TGF\text{-}\beta1}$ for 48 h. The cell proliferation marker Ki67 (red) and nuclei (DAPI: blue) are labeled. Scale bars, 50 μm. Quantification of Ki67-positive cells expressed as percent of positive cells relative to the total number of cells under each condition. **(B)** Cell viability assay with MDA-MB-231 cells incubated for 48 h with vehicle (DMSO), EV-free media, VSF^{Ctrl}, supernatant depleted from CD81-positive EV^{Ctrl}, $VSF^{+TGF\text{-}\beta1}$ or supernatant depleted from CD81-positive $EV^{+TGF\text{-}\beta1}$, followed by the presence or absence of DMSO (Ctrl), 0.5 μM doxorubicin (Dox) or 0.25 μM paclitaxel (Taxol). **(C-E)** Cell viability assay with non-tumorigenic breast epithelial MCF10A (C), normal breast fibroblastic BNF3 (D) and the BRCA MCF7 cells incubated with vehicle (Ctrl) and 1×10^9 nanoparticles of control EVs (VSF^{Ctrl}) or TGF-β1-induced EVs ($VSF^{+TGF\text{-}\beta1}$) from MDA-MB-231 (MDA) cells for 48 h. **(F)** Cell viability assay with ZR-75-1 cells incubated with vehicle (Ctrl) and 1×10^9 nanoparticles of VSF^{Ctrl} or $VSF^{+TGF\text{-}\beta1}$ from MDA cells in the absence or presence of 0.5 μM Dox or 0.5 μM taxol for 48 h. **(G, H)** Cell viability assay with MDA-MB-231 (G) and ZR-75-1 (H) cells incubated with buffer (CTRL) or EVs (VSF) from non-tumorigenic breast epithelial MCF10A cells for 48 h. The cells were also co-incubated in vehicle (DMSO), Dox or taxol. **(I)** Cell viability assay with MDA-MB-231 cells incubated with vehicle (DMSO), 5 ng/ml TGF-β1 or 5 μM LY2157299 TGF-β type I receptor inhibitor (TβRi) for 48 h. **(J, K)** Cell viability assay with MDA-MB-231 cells treated with 0.25 μM Taxol (J) or 0.5 μM Dox (K) in all conditions and co-incubated with vehicle (DMSO) or 5 μM TβRi, during simultaneous co-incubations with vehicle (DMSO), 5 ng/ml TGF-β1, with equal number of VSF^{Ctrl} or $VSF^{+TGF\text{-}\beta1}$ from MDA-MB-231 cells for 48 h. The data in A, C-E and I are presented as mean values of three biological replicates ± SEM, in technical triplicates and p-values are shown based on one-way ANOVA, followed by multiple paired comparisons conducted by means of Bonferroni's post-test method. The data in B, F-H, J and K are presented as mean values of three biological replicates ± SEM, in technical triplicates and p-values are shown based on two-way ANOVA, followed by multiple paired comparisons conducted by means of Bonferroni's post-test method: $^*p \leq 0.05$; $^{**}p \leq 0.01$; $^{***}p \leq 0.001$; $^{****}p \leq 0.0001$.

SUPPLEMENTARY TABLES

Supplementary Table S1. Gene Set Enrichment Analysis (GSEA): pathways signature database.

**EMT**							
ABI3BP	ACTA2	ADAM12	ANPEP	APLP1	AREG	BASP1	BDNF
BGN	BMP1	CADM1	CALD1	CALU	CAP2	CAPG	CCN2
CD44	CD59	CDH2	CDH6	CDH11	COL11A1	COL12A1	COL16A1
COL1A1	COL1A2	COL3A1	COL4A1	COL4A2	COL5A1	COL5A2	COL5A3
COL6A2	COL6A3	COL7A1	COL8A2	COLGALT1	COMP	COPA	CRLF1
CTHRC1	CXCL1	CXCL12	CXCL6	CXCL8	DAB2	DCN	DKK1
DPYSL3	DST	ECM1	ECM2	EDIL3	EFEMP2	ELN	EMP3
ENO2	FAP	FAS	FBLN1	FBLN2	FBLN5	FBN1	FBN2
FERMT2	FGF2	FLNA	FMOD	FN1	FOXC2	FSTL1	FSTL3
FUCA1	FZD8	GADD45A	GADD45B	GAS1	GEM	GJA1	GLIPR1
GPC1	GPX7	GREM1	HTRA1	ID2	IGFBP2	IGFBP3	IGFBP4
IL15	IL32	IL6	INHBA	ITGA2	ITGA5	ITGAV	ITGB1
ITGB3	ITGB5	JUN	LAMA1	LAMA2	LAMA3	LAMC1	LAMC2
LGALS1	LOX	LOXL1	LOXL2	LRP1	LRRC15	LUM	MAGEE1
MATN2	MATN3	MCM7	MEST	MFAP5	MGP	MMP1	MMP2
MMP3	MMP14	MSX1	MXRA5	MYL9	MYLK	NID2	NNMT
NOTCH2	NT5E	NTM	OXTR	P3H1	PCOLCE	PCOLCE2	PDGFRB
PDLIM4	PFN2	PLAUR	PLOD1	PLOD2	PLOD3	PMEPA1	PMP22
POSTN	PPIB	PRRX1	PRSS2	PTHLH	PTX3	PVR	QSOX1
RGS4	RHOB	SAT1	SCG2	SDC1	SDC4	SERPINE1	SERPINE2
SERPINH1	SFRP1	SFRP4	SGCB	SGCD	SGCG	SLC6A8	SLIT2
SLIT3	SNAI2	SNTB1	SPARC	SPOCK1	SPP1	TAGLN	TFPI2
TGFB1	TGFBI	TGFBR3	TGM2	THBS1	THBS2	THY1	TIMP1
TIMP3	TNC	TNFAIP3	TNFRSF11B	TNFRSF12A	TPM1	TPM2	TPM4
VCAM1	VCAN	VEGFA	VEGFC	VIM	WIPF1	WNT5A	

**TGF-β signaling**							
ACVR1	APC	ARID4B	BCAR3	BMP2	BMPR1A	BMPR2	CDH1
CDK9	CDKN1C	CTNNB1	ENG	FKBP1A	FNTA	FURIN	HDAC1
HIPK2	ID1	ID2	ID3	IFNGR2	JUNB	KLF10	LEFTY2
LTBP2	MAP3K7	NCOR2	NOG	PMEPA1	PPM1A	PPP1CA	PPP1R15A
RAB31	RHOA	SERPINE1	SKI	SKIL	SLC20A1	SMAD1	SMAD3
SMAD6	SMAD7	SMURF1	SMURF2	SPTBN1	TGFB1	TGFBR1	TGIF1
THBS1	TJP1	TRIM33	UBE2D3	WWTR1	XIAP		

**Cholesterol homeostasis**							
ABCA2	ACAT2	ACSS2	ACTG1	ADH4	ALCAM	ALDOC	ANTXR2
ANXA5	ANXA13	ATF3	ATF5	ATXN2	AVPR1A	CBS	CD9
CHKA	CLU	CPEB2	CTNNB1	CXCL16	CYP51A1	DHCR7	EBP
ECH1	ERRFI1	ETHE1	FABP5	FADS2	FASN	FBXO6	FDFT1
FDPS	GLDC	GNAI1	GPX8	GSTM2	GUSB	HMGCR	HMGCS1
HSD17B7	IDI1	JAG1	LDLR	LGALS3	LGMN	LPL	LSS
MAL2	MVD	MVK	NFIL3	NSDHL	PCYT2	PDK3	PLAUR
PLSCR1	PMVK	PNRC1	PPARG	S100A11	SC5D	SCD	SEMA3B
SQLE	SREBF2	STARD4	STX5	TM7SF2	TMEM97	TNFRSF12A	TP53INP1
TRIB3							

Supplementary Table S2. Primer sequences used for RT-qPCR assays.

Gene Symbol	**Forward** (F) **and Reverse** (R) **Primers Sequences (5'- 3')**
BMP4	F - GCACTGGTCTTGAGTATCCTG<br>R - TGCTGAGGTTAAAGAGGAAACG
CDK1	F - AGGAACCCCTTCCTCTTCACT<br>R – CGCCGCGGAATAATAAGCC
DHCR7	F - GCGGAGGTAGGTCTTTCACA<br>R – CCATTTCGCCATAGAACCAT
DHCR24	F - ATGGCAGCTTTGTGCGAT<br>R – ACGCAGCTTGACGTACTTCT
FABP5	F - CCTGTCCAAAGTGATGATGG<br>R – CAGCATCAGGAGTGGGATG
GAPDH	F - GGAGTCAACGGATTTGGTCGTA<br>R - GGCAACAATATCCACTTTACCA
HMGCR	F - AGTCACAAGCACGTGGAAGACG<br>R - TTGGTGATGGGAGCTTGCTGTG
ITGA6	F - TCTGCGGAGGTAACGGCGACT<br>R - TCCGTGCTGGTGGTGCAGTTG
ITGFB5	F - GCCTTTCTGTGAGTGCGACAAC<br>R - CCGATGTAACCTGCATGGCACT
LDLR	F - GACTGGTCAGATGAACCCATCAAAG<br>R - AGGTCATTGCAGACGTGGGAAC
MMP2	F - AGATGCCTGGAATGCCAT<br>R – GGTTCTCCAGCTTCAGGTAAT
MMP9	F - TACTGTGCCTTTGAGTCC<br>R – TTGCGGCGATAAGGAAG
MMP10	F - CCCCTGGTGCCCACAAA<br>R – TCACACTTGGCTGGCATCTC
MMP14	F - TCCAGCAACTTTATGGGGGT<br>R – TTCCCGTCACAGATGTTGGG
MVD	F - GTGTCGAGGCAGGTGGCGTGG<br>R - CCTGAACTCCGCGTGCTCATCC
NOG	F - CAGCACTATCTCCACATCCG<br>R - TCGTTCAGATCCTTTTCCTTGG
PMVK	F - CTTCGTGACCGAGGCGCTGC<br>R - GAGATGCCCTCCACAATCTTCCTGC
SC5D	F - CGTGTATCCAGCCACATGGCC<br>R - GAATACTTATCCATGGCAATGCCTGG
SERPINE1	F - GAGACAGGCAGCTCGGATTC<br>R – GGCCTCCCAAAGTGCATTAC
SQLE	F - TGACAATTCTCATCTGAGGTCCA<br>R - CAGGGATACCCTTTAGCAGTTTT
STARD4	F - CTCTACAAAGCCCAAGGTG<br>R – TCATCAAGCTGTCCCAATC
TGFB1	F - GCCTTTCCTGCTTCTCATGG<br>R – TCCTTGCGGAAGTCAATGTAC
TGFBI	F - AATCGCTTTAGCATGCTGGT<br>R – CAAGAGTCTGCTCCGTTCTC
TGFBR3	F - CTGTTCACCCGACCTGAAAT<br>R – CGTCAGGAGGCACACACTTA

Supplementary Table S3. List of antibodies used with applications, dilution factors and commercial IDs.

Antibody	Application	Manufacturer and Cat. #
Anti-SMAD2	Immunoblotting (1:1000)	Cell Signaling #5339S
Anti-SMAD3	Immunoblotting (1:1000)	Cell Signaling #9523S
Anti-Phospho-SMAD3 (pSMAD3)	Immunoblotting (1:1000)	Cell Signaling #9520S
Anti-p44/42 MAPK (ERK1/2)	Immunoblotting (1:1000)	Cell Signaling #4695
Anti-Phospho-ERK1/2 (pERK1/2)	Immunoblotting (1:1000) Immunofluorescence (1:200)	Cell Signaling #4370S
Anti-E-Cadherin (CDH1)	Immunoblotting (1:1000)	Cell Signaling #3195S
Anti-Caspase3	Immunoblotting (1:1000)	Cell Signaling #9662
Anti-Cleaved PARP1	Immunoblotting (1:1000)	Cell Signaling #5625
Anti-AKT	Immunoblotting (1:1000)	Cell Signaling #4691
Anti-Phospho-AKT (pAKT)	Immunoblotting (1:1000)	Cell Signaling #2965
Anti-Phospho-SMAD2 (pSMAD2)	Immunoblotting (1:1000)	Millipore #AB3849
Anti-FIBRONECTIN (FN1)	Immunoblotting (1:1000) Immunofluorescence (1:150)	Millipore #F3648
Anti-PAI1	Immunoblotting (1:1000)	BD Biosciences #612025
Anti-β-TUBULIN	Immunoblotting (1:1000)	BD Biosciences #556321
Anti-DHCR7	Immunoblotting (1:500)	Abcam #ab103296
Anti-ALIX	Immunoblotting (1:250) Immunoprecipitation 2.0 µg	SantaCruz Biotechnology #sc-53540
Anti-CD9	Immunoblotting (1:250)	SantaCruz Biotechnology #sc-59140
Anti-CD81	Immunoblotting (1:250)	SantaCruz Biotechnology #sc-166029
Anti-TSG101	Immunoblotting (1:250)	SantaCruz Biotechnology #sc-136111
Anti-β-ACTIN	Immunoblotting (1:1000)	SantaCruz Biotechnology #sc-69879
Anti-α-SMOOTH MUSCLE ACTIN (α-SMA)	Immunofluorescence (1:200)	Sigma-Aldrich #A2547
Anti-GAPDH	Immunoblotting (1:1000)	ThermoFisher Scientific #AM4300
Anti-SREPB2	Immunoblotting (1:500)	ThermoFisher Scientific #PA1-338
Anti-Phospho-SREPB2	Immunoblotting (1:500)	ThermoFisher Scientific #PA5-106042
Anti-Ki67	Immunofluorescence (1:200)	Abcam #ab15580
Goat anti-Mouse IgG (H+L) Secondary Ab	Immunoblotting (1:10000)	ThermoFisher Scientific #62-6520
Goat anti-Rabbit IgG (H+L) Secondary Ab	Immunoblotting (1:10000)	ThermoFisher Scientific #65-6120
Goat anti-Rabbit IgG (H+L) Highly Cross-Adsorbed Secondary Ab, Alexa Fluor-594	Immunofluorescence (1:1000)	ThermoFisher Scientific #A-11037
Goat anti-Mouse IgG (H+L) Highly Cross-Adsorbed Secondary Ab, Alexa Fluor-546	Immunofluorescence (1:1000)	ThermoFisher Scientific #A-11030

Donkey anti-Mouse IgG (H+L) Cross-Adsorbed Secondary Ab, Alexa Fluor-488	Immunofluorescence (1:1000)	ThermoFisher Scientific #A-21202
Donkey anti-Rabbit IgG (H+L) Highly Cross-Adsorbed Secondary Ab, Alexa Fluor-488	Immunofluorescence (1:1000)	ThermoFisher Scientific #A-21206

Supplementary Table S4. List of siRNAs used in this study.

SiRNA	Product ID
SMARTpool: ON-TARGETplus Human CD81	Dharmacon; L-017257-00-0005
SMARTpool: ON-TARGETplus SMAD2	Dharmacon; L-003561-00-0005
SMARTpool: ON-TARGETplus SMAD3	Dharmacon; L-020067-00-0005
ON-TARGETplus non-targeting pool	Dharmacon; D-001810-10-20
FlexiTube GeneSolution for DHCR7	Qiagen; 1027416 ID:1717
FlexiTube GeneSolution for SREBP2	Qiagen; 1027416 ID:6721
Negative Control siRNA	Qiagen; 1022076

Supplementary Table S5. Unprocessed mass spectrometry data.
Worksheet with the quantification values for all the proteins identified by mass-spectrometry in each sample of this study. Protein abundance is expressed as peak areas (signal intensity) and expresses the relative abundance of each protein in each sample. The worksheet includes three sheets: All data; Quantified data; Legends.

Supplementary Table S6. List of unique proteins in CD81-EVs isolated from MDA-MB-231 cells.

**List of 303 unique proteins in CD81-EV^{Ctrl}**							
6PGD	A16A1	AATM	ACOT1	ACTBL	ACTN2	ACTZ	ADHX
AIMP1	AIP	AL7A1	ANM1	ANXA3	AP1S1	AP2A1	ARF5
ARI1	ARPC2	ASNS	ATS1	B2MG	BACH	BASP1	BZW1
BZW2	CALR	CAND1	CAPZB	CATZ	CAVN1	CAZA2	CBX3
CC124	CCD50	CD166	CFAB	CHM1B	CHM2A	CHM4A	CIRBP
CK068	CLH1	CNPY2	CO040	CO1A2	CO6A1	COMD9	COPG1
CPSF5	CSRP1	CSTF1	CSTF3	CUL4B	DC1L1	DC1L2	DCD
DCPS	DCTN2	DDAH2	DDX1	DEOC	DHX15	DIAP1	DKK3
DNJB4	DRG1	DSG2	DUS3	DUT	DYN2	EF1D	EF1G
EIF3C	EIF3D	EIF3G	EIF3I	EIF3L	ELOC	EMAL4	EP15R
ERO1A	ESYT1	ETFA	ETFB	ETHE1	FKBP3	G6PD	GARS
GBB1	GBB2	GDS1	GFPT1	GLU2B	GSTM2	GSTM3	GUAA
HARS1	HAT1	HBA	HEBP1	HINT1	HLAB	HMCS1	HNRDL
HSPB1	IBP1	ICAL	IF2G	IF2P	IF4E	IF5	ILEU
ILRL1	IMA1	IMDH2	IMPA1	IPO5	IPO7	IPYR2	IST1
ITIH2	KAP2	KCRB	KIF23	KINH	LA	LAMA5	LASP1
LAT1	LFA3	LIS1	LSM3	LSR	M4K4	MACF1	MAP4
MARE1	MATN2	MATR3	MCA3	MCM6	MET	MGAT1	MGP
MOB1B	MPRI	MSLN	MTPN	MUC18	MVP	NAA50	NASP
NDRG1	NECP2	NEUR1	NH2L1	NIBA1	NIT1	NNMT	NOGG
NPL4	NUDT4	ODP2	OLM2A	OS9	PBDC1	PCKGM	PCNP
PDCD5	PDLI1	PFD3	PFD5	PFKAP	PGM2	PHOCN	PIN4
PLIN3	PLOD2	PLOD3	PLSI	PMM2	PP1B	PP1R8	PP2BA
PPAC	PPCS	PPID	PPM1G	PPME1	PRDX4	PRS7	PSA
PSA5	PSMD2	PTBP1	PTN11	PTX3	PUR2	PYRG1	RAB10
RAB13	RAB14	RAB5C	RAB6A	RAB8A	RAB8B	RACK1	RADI
RALA	RANG	RB11B	RBBP4	RBBP7	RBMX	RHEB	RL29
RMD1	RMXL1	RNPS1	ROA3	RRBP1	RS14	RS15A	RS18
RS21	RS25	RS8	RTCB	RU2B	RUVB2	RUXGL	S10A6
S10AB	SAE2	SAFB1	SBDS	SDC1	SEPTIN11	SEPTIN9	SERC
SERC1	SF01	SF3A1	SF3B2	SF3B3	SMU1	SPB6	SPB9
SPEE	SPRE	SPSY	SPTN1	SRP14	SRP19	SRP68	SRP72
SRSF1	SRSF9	SSBP	STAU1	STC2	STX7	SUMO2	SYCC
SYDC	SYEP	SYK	SYLC	T4S1	TCPA	TCPB	TCPE
TCTP	TFPI1	TFR1	THOP1	TIM44	TINAL	TM1L1	TOP1
TPIS	TPM1	TR112	TSN14	TSN5	TXND5	UBA3	UBC12
UBP10	UBP2L	UBXN1	UFD1	ULA1	VAMP3	VATA	VCAM1
VIGLN	VWA1	WASH6	XPP1	XRN2	YKT6		

**List of 143 unique proteins in CD81-EV$^{+TGF-\beta1}$**							
ACBP	ACO2	ADAM17	AIDA	ALB	AMIGO2	ANXA7	AP2M1
ARPC5L	ARRDC1	B4GALT4	BAF	BCL2L2	BLVRB	BMP6	BROX
C1GALT1C1	CACYBP	CALB2	CALD1	CALML5	CBR3	CCN2	CD2AP
CD9	CDH4	CDSN	CHID1	CHST14	CLDN1	COL1A1	COL3A1
COL7A1	COPE	CST1	CSTN3	CYRIB	CYTS	DNAJC9	EEF1A2
EGFR	EHD2	EIF3J	EIF3K	EPHB2	EPS8L2	EVA1A	FAM98B
FBN1	FEN1	GAS6	GBP2	GDI2	GELS	GET3	GIPC1
GNAI3	GPI	GRHPR	HHIP	HMGB2	HNRPR	HSPA13	IGBP1
IL11	ITGA2	ITGA5	ITGAV	KIRREL1	LAMB2	LDLR	LFNG
LMAN1	LMCD1	LOX	LSM4	LTBP2	LTBP3	LTBP4	MANF
MAP1B	MAT2A	MDH1	MMP2	MMP9	MMP10	MPZL1	MTHFD1
MYH1	MYH2	MYH7	NRP1	NUCKS	NUDC	PA2G4	PABP4
PAK1	PIMT	PRSS23	PSMC3	PSMC5	PSME3	PSPC1	PTPRK
PYGM	RAP1A	RAP1B	RGMB	RHOG	RPA3	RPL19	RPL27A
RPLP0	RPS6	S100A10	SAE1	SEPTIN2	SF3B6	SMD1	SNAP23

SORT	SRSF7	STRAP	SYNC	TAGL	TEBP	TGM2	TIM13
TOR1B	TSP2	TSPAN6	TWF2	UBE2Z	UBQLN1	ULBP2	USP14
VASN	VPS37C	VTA1	ZA2G	ZC3H15	ZC3HAV1	ZNF207	

Supplementary Table S7. List of unique proteins in CTB-EVs isolated from MDA-MB-231 cells.

**List of 319 unique proteins in CTB-EV^{Ctrl}**							
AAK1	ABCC8	ABCF1	ACIN1	ADA	ADAM8	ADAR	AFG3L2
AFP	AHSG	AKAP2	ALDH2	ANG	ANK2	AP3D1	APOH
ARHGAP29	ARHGDIB	ARHGEF5	ARHGEF7	ARPC3	ARRDC1	ATG16L1	ATP1B1
ATP6V1B2	ATP6V1C1	ATP6V1D	AZGP1	BAG2	BCAR1	BIN2	BMP4
BPNT2	BRD2	BRK1	BST2	C1RL	CAPNS1	CARHSP1	CARM1
CAVIN3	CBFB	CBX5	CCBE1	CCS	CD59	CDK1	CELF1
CIAO2A	CIAO2B	CIAPIN1	CKAP4	CLIC6	CLUH	CNTNAP3	COMMD8
COPZ1	CPSF7	CPVL	CRABP2	CRAT	CSTF1	CSTF2	CTR9
CUL3	DAP	DBNL	DDX3X	DENR	DIABLO	DLG1	DNAJB4
DNAJC8	DR1	DRAP1	DUT	EFNB2	EIF2B2	EIF3J	EIF4H
EPN2	EPS8	EPS8L2	ESM1	ETFA	F10	F2	F5
FAHD2A	FAM114A2	FAM98A	FBP1	FDX2	FKBP5	FMNL1	FN3KRP
FNBP1	FUBP3	GABARAP	GABARAPL1	GALNT6	GAPVD1	GCNT2	GFPT2
GINS1	GMPPA	GMPR2	GNA11	GNG12	GNPDA2	GOLGA6L1	GRSF1
GRWD1	GSTM3	GTF2F1	HADH	HDAC1	HDHD3	HERC4	HGS
HIP1R	HK2	HLA-DRA	HLA-DRB1	HLA-DRB3	HOMER3	HS3ST1	HSBP1
HSP90B2P	HSPA4L	HYPK	ICOSLG	IPO4	IRF2BP2	ITPA	KCTD12
KITLG	LARS2	LCMT1	LEMD2	LIPH	LOXL4	LRPAP1	LRRC40
LSM8	LYPLAL1	MAD2L1	MAP1A	MAPK13	MAPKAPK2	MAPKAPK3	MDK
MIDEAS	MNAT1	MPST	MRE11	MRI1	MSRA	MTAP	MTPN
MVK	MYCBP	NAA20	NCAM1	NDNF	NECTIN3	NEK9	NEMF
NENF	NISCH	NME7	NNMT	NOP2	NOP56	NPEPL1	NPTN
NQO1	NT5DC3	NUDCD2	NUDCD3	NUDT3	NUP93	OARD1	OLFML2A
OTUB2	OTUD6B	PAPSS1	PCDHB14	PCDHB16	PDCD4	PDCL3	PDE5A
PDHX	PDP1	PHOX2B	PITHD1	PLCG1	PLG	PM20D2	PMM2
PMPCB	POLR2G	POLR3B	PPIL4	PPP1R2	PRKAR1A	PRKDC	PRMT3
PRNP	PRPSAP2	PSMC5	PSMD10	PSMD6	PSMF1	PSMG1	PSMG4
PTBP2	PTER	PTGR2	RAB34	RABGGTA	RAC2	RAD23A	RALA
RANGRF	RBBP9	RBM15	RPA2	RPAP3	RPL11	RPL24	RPL30
RPL8	RPS12	RPS4X	RPS7	RSL1D1	RTCA	S100A6	SAA1
SART3	SCAF1	SDSL	SEC23B	SECTM1	SEMA3B	SEMA5A	SETD3
SF1	SF3B2	SH3GL1	SIRT5	SLC1A4	SLC38A2	SLC39A14	SLIT2
SMARCC2	SMC1A	SMOC1	SMU1	SNRNP70	SNRPB2	SNRPGP15	SNU13
SNX5	SP100	SP8	SPRING1	SRP54	SRPK1	SRPRA	SRRM1
SRRT	SRSF7	STUB1	SYMPK	TACO1	TBC1D13	TGFBR3	TIAL1
TIMM44	TIMP3	TKFC	TMA16	TMEM106B	TOM1L1	TOMM34	TOP1
TP53BP1	TRIM25	TRIR	TRMT61A	TST	TTC9C	TYMP	UBE2S
UBQLN4	UBR4	UBXN1	UBXN7	UCHL5	UFC1	UTP25	UTRN
VPS28	VSIR	XXYLT1	ZC3H14	ZFYVE26	ZG16B	ZNRD2	
**List of 275 unique proteins in CTB-EV$^{+TGF\text{-}\beta1}$**							
2A5E	AAMP	AAR2	ABHD10	ABHD14A	ACP2	ADAM19	ADAMTS7
AF10	AFAP1L2	AGO1	AGO3	AIDA	AIMP2	AKAP12	AMPD2
ANGPTL4	ANTXR2	AP1S1	APLP1	APOB	ARFGAP1	ARLY	ARPP19
ARSK	ATF6A	ATP2B1	ATP5IF1	BABAM1	BCLAF1	BPHL	BRE1B
C19L1	CADM4	CAMKV	CDH5	CDH6	CEP170	CGB1	CHMP4B
CHST3	CKS1	CND1	CNOT1	COL3A1	COL4A1	CRELD1	CRIP2
CSF3	CSNK2A1	CTHRC1	CTNNBL1	DCBLD1	DCLK1	DCTN6	DCTPP1
DDR1	DECR1	DIP2B	DNAJB1	DNAJC10	DNAJC17	DNLI1	DTYMK
DYN2	DYNC1LI2	DYNLL1	DYNLT1	EDC4	EDN1	EFNA5	EFNB1
EIF1A	EIF2AK2	EIF2B3	EIF2B5	EIPR1	ELMO2	ENG	EPB41L2
EXOSC9	FAM234A	FAM83H	FAN	FBN2	FBXL18	FGFRL1	FHIT
FLRT2	FMOD	FNBP1L	FRAS1	FURIN	G3BP2	GABARAPL2	GALNT12
GALNT16	GALT3	GEPH	GILT	GLT8D1	GOLGA7	GOLM2	GRID2
GSK3B	GUK1	HDGFL3	HDHD1	HNRNPUL2	HOOK3	HPLN3	HS3ST3B1
IAH1	IBP2	IDH3A	IDUA	IGBP1	IGSF3	INPP1	IPO11

ISG20	ITGB5	ITIH4	ITM2B	JAG1	JAM1	KIF14	KLC1
KRT13	LAGE3	LAMTOR5	LDHC	LFG3	LFNG	LGALS8	LHFPL2
LOXL1	LRP6	LRSAM1	LSM2	LSM3	LSM7	LSR	LTBP2
LUM	LYAR	LZIC	LZTFL1	MAPK9	MATN3	MEGF8	MICB
MIPEP	MIS	MMP13	MSS4	MVP	MYADM	NAGA	NECAP2
NET1	NMI	NPTXR	NT5C	NTAN1	NUDT15	NUMB	OCRL
ODAPH	PARD3	PAWR	PCDH1	PCDHB13	PCDHGC5	PDLIM4	PGP
PLPBP	PMP2	POGLUT3	POLDIP2	POR	PRPF4B	PSG5	PSMD14
PTHR	PURA	PXDC2	PXN	QPCTL	RANBP3	RAP2B	RBP4
RELA	RFTN1	RHEB	RHG12	RHOG	RIC8A	RIDA	RIFK
RMD3	RPA34	RPL19	RPL7	RPL7A	RPN2	RPS5	RRAS
RRM2	RRP44	RS11	RTN4	S100A3	SARNP	SDC1	SEH1
SEMA4C	SEPR	SERPINA9	SF3B4	SHC1	SIR2	SIRPB1	SMAP
SMARCA1	SMARCA5	SMC3	SMC4	SMD2	SMYD5	SNAG	SNF8
SNX6	SOWAHC	SPART	SRA1	SRRM2	SSU72	ST3GAL2	STAMBPL1
STRN	STX7	SUMF2	SVIL	TAGL	TARDBP	TARSH	TATDN1
TBL1X	TCN2	TGOLN2	THSD4	TIM8A	TIM9	TNFSF9	TOR1AIP1
TPRKB	TRIO	TSPAN9	TXN2	TXNDC16	TYMS	UBE2H	UBE2R2
UBE2T	UHRF1	VEGFA	VPS4A	VPS4B	VSTM2L	WASF2	WASL
WDR13	XPO7	YTHDF2					
